# High-dimensional Parameter Transfer With Fused-Regularizer

**Published:** 2026

**Authors:** Zelin He, Ying Sun, Jingyuan Liu, Runze Li

**Affiliations:** Department of Statistics, Pennsylvania State University, University Park, PA 16802, USA; School of EECS, Pennsylvania State University, University Park, PA 16802, USA; Department of Statistics and Data Science, School of Economics, Xiamen University Xiamen, Fujian 361102, China; Department of Statistics, Pennsylvania State University, University Park, PA 16802, USA

**Keywords:** transfer learning, high-dimensional estimation, regularized M-estimator, non-asymptotic theory, sparsity

## Abstract

Parameter transfer aims to improve parameter estimation accuracy by leveraging knowledge from related sources. This paper studies the parameter transfer problem from heterogeneous sources for high-dimensional M-estimators. Specifically, we propose a novel one-step estimator with a fused-regularizer and a target-data-oriented constraint, which can robustly capture parameter knowledge from source data in the presence of different types of data distribution shifts. Nonasymptotic bound is provided for the estimation error of target parameter, showing the proposed estimator could achieve effective parameter transfer under distribution shifts, and is guaranteed to perform no worse than any estimators learned only from the target data. We further show that the proposed estimator can achieve the minimax-optimal rate under much weaker conditions than existing methods. In addition, we extend the method to a distributed setting, requiring just one round of communication with source parameter estimators, while retaining the estimation accuracy of the centralized version. Extensive simulations and real data analysis further verify the effectiveness of the method.

## 1 Introduction

High-dimensional parameter estimation methods have gained increasing attention due to their wide application in areas including bioinformatics, astronomy, and information technology (Bühlmann and Van De Geer, 2011). With the dimension of unknown parameters being much larger than the sample size, the parameter estimation becomes much more challenging due to issues of error accumulation (Fan et al., 2020). Nonetheless, existing methods primarily rely on independent and identically distributed (i.i.d.) data. In many scenarios, obtaining such high-dimensional i.i.d. data is costly or infeasible, posing challenges to accurate parameter estimation and reliable decision-making.

Transfer learning is a promising framework that can improve the parameter estimation accuracy for the above low-data setting by incorporating side information from auxiliary source samples (Torrey and Shavlik, 2010). Specifically, this paper considers a parameter transfer setting, where we are given a target sample drawn from a target distribution $P^{\left(0\right)}$ parameterized by a high-dimensional target parameter $\beta_{*}^{\left(0\right)}$, along with some auxiliary samples drawn from K different source distributions ${\left\{P^{\left(k\right)}\right\}}_{k=1}^{K}$ parameterized by ${\left\{\beta_{*}^{\left(k\right)}\right\}}_{k=1}^{K}$. The goal is to improve the estimation accuracy of the target parameter by incorporating *parameter knowledge* from other source samples (Pan and Yang, 2009). We consider a challenging scenario where the similarity between the source and target is completely captured through the closeness of $\beta_{*}^{\left(0\right)}$ and $\beta_{*}^{\left(k\right)}$, with minimal additional structural similarity assumptions among the data distributions. Such a flexible setup encapsulates various types of distribution shifts of practical interests. For example, in a regression setting, it includes shifts in the marginal covariate distributions (He et al., 2024). In high-dimensional scenarios, such a covariate shift can be more severe due to a more complex covariate correlation structure (Nagel et al., 2018; Charney et al., 2017). Besides covariate shifts, the distribution shifts may also come from the shift in the conditional distribution of the responses. For example, in genomic studies, some source data may suffer from overdispersion due to a higher-than-expected variance (Wang et al., 2019). In the presence of distribution shifts, naively incorporating source samples can lead to estimation accuracy that is worse than using the target sample alone–a phenomenon known as “negative transfer” (Zhang et al., 2023). The challenge calls for the design of a high-dimensional parameter transfer procedure that can effectively exploit parameter similarity under these distribution shifts and can always prevent negative transfer.

Beyond challenges brought by distribution shifts, another challenge that often arises in transfer learning is the distributed nature of source samples. For example, for electronic health record data analysis in multicenter research studies, source samples are collected from different institutes or organizations, where direct data sharing is often impractical due to privacy and regulation constraints or the prohibitive communication cost (Kushida et al., 2012). This necessitates the development of privacy-preserving and communication-efficient solutions that can achieve comparable estimation accuracy as their centralized counterparts.

### 1.1 Contribution of this paper

To address the above challenges, we propose a parameter transfer framework for high-dimensional M-estimators, named *TransMission*, that introduces a series of fused-regularizers into a joint learning process combined with a novel target-data-oriented constraint. Joint learning with fused regularization captures transferable parameter knowledge across sources while maintaining robustness to a wide range of distribution shifts. The target-data-oriented constraint further narrows the search space for the target parameter using the target data, so the estimator will always perform no worse than the single-task estimator learned only on the target data, preventing the issue of negative transfer.

We further support the framework with a fine-grained, non-asymptotic theoretical analysis. We consider a p-dimensional M-estimation problem for an s-sparse target parameter $\beta_{*}^{\left(0\right)}$. With a target sample of size $n_{T}$, and K source samples with each of size $n_{S}$, we show that *TransMission* yields a convergence rate of $O\left(\frac{s\;\text{log}\;p}{n_{T}+Kn_{S}}+\left(h\sqrt{\frac{\text{log}\;p}{n_{T}}}\right)\wedge \left(C_{\Sigma }^{2}h^{2}\right)\wedge \frac{s\;\text{log}\;p}{n_{T}}\right)$, where h measures difference between the source parameter $\beta_{*}^{\left(k\right)}$ and the target parameter $\beta_{*}^{\left(0\right)}$, and $C_{\Sigma }$ measures the degree of distribution shifts. The result carries several merits. First, it shows *TransMission* is always no worse than the rate of the single-task estimator, i.e., $O\left(\frac{s\;\text{log}\;p}{n_{T}}\right)$, therefore preventing negative transfer thanks to the introduction of the target-data-oriented constraint. When the source parameters are sufficiently close to the target (h is small), *TransMission* is capable of taking full advantage of the source samples and attaining a much faster rate of $O\left(\frac{s\;\text{log}\;p}{n_{T}+Kn_{S}}\right)$. In addition, it reveals *TransMission* is robust to severe distribution shifts as reflected by a large value of $C_{\Sigma }$. Overall, the estimator achieves a sharper rate than many state-of-the-art methods under various scenarios, and the rate matches the existing minimax lower bound under a much weaker condition on h. We refer the readers to Section 3.3 for a more detailed comparison.

To address practical concerns in distributed settings, we also develop its variant termed *D-TransMission* that requires only one-shot transmission of the pre-estimated parameters from source tasks. We further show that *D-TransMission* achieves a statistical rate of the same order as *TransMission*, provided the source sample size is sufficiently large.

### 1.2 Related Work

Much progress has been made in parameter transfer in recent years. However, many only focus on capturing the shared parameter information, but either overlook other distribution shifts entirely or focus on mitigating specific shifts. For example, a line of approach is based on a two-step approach, which first pools source and target data for knowledge transfer and then applies a bias correction using only the target data (Bastani (2021); Li et al. (2022); Tian and Feng (2023)). However, such an approach does not explicitly address other distribution shifts, and the learning accuracy degrades quickly if the shifts are severe. Recently there have been a few pioneering works proposed to correct covariate shifts with imputation models (Liu et al., 2023; Zhou et al., 2025; Cai et al., 2025). However, the effectiveness of these methods depends on the availability of abundant unlabeled target data, and they do not account for other types of distribution shifts beyond covariate shifts.

Achieving a robust transfer of learnable parameter information has been investigated over the years in the multi-task setting, where regularization techniques are often employed to promote the exchange of parameter knowledge across different tasks (Pan and Yang, 2009), examples including $\ell_{2}$-norm (Duan and Wang, 2023; Evgeniou et al., 2005), $\ell_{1}$-norm (Li and Sang, 2019; Zhang et al., 2024), Euclidean-norm (Chen et al., 2023) and spectral-norm (Tian et al., 2025), to name a few. Most of these works borrow the idea of fused-lasso (Tibshirani et al., 2005) to promote parameter information sharing. These multi-task learning methods typically require all the tasks to have a comparable sample size and emphasize overall task performance. Therefore, they are not directly applicable to transfer learning problems where the target task, sometimes with far fewer samples, is the primary focus. Gao and Yang (2023), Liu (2024) and Long et al. (2013) utilize a similar fused-regularization structure to promote parameter transfer, yet they either only apply in the low-dimensional setting or require the source and target to have comparable sizes. Li et al. (2024) and Bai et al. (2024) propose robust methods for high-dimensional parameter transfer. However, the proposed methods either lack guarantees for preventing negative transfer or incur non-negligible costs to achieve such robustness. See Section 3.3 for a detailed comparison.

A closely related work is TransFusion (He et al., 2024). Compared with TransFusion, this paper makes several substantial new contributions. First, TransFusion is limited to linear regression and only guarantees robustness of parameter transfer under covariate shift, whereas this paper develops a general framework for parameter transfer applicable to M-estimators and accommodates a broader class of distribution shifts. Second, this paper proposes an entirely new one-step estimator with a novel target-data-oriented constraint for general M-estimators, which guarantees the estimator always performs no worse than estimators learned only from the target data; by contrast, TransFusion relies on a two-step approach and can fail when the source tasks are not transferable. Third, this paper develops several new theoretical results for the proposed estimator, improves the convergence rate of the estimated target parameter, and establishes minimax optimality under weaker conditions than those required in TransFusion.

### 1.3 Organization and Notation

The remainder of the paper is organized as follows. Section 2 introduces the setup of parameter transfer for high-dimensional M-estimator. In Section 3, we propose the *TransMission* framework, provide a theoretical characterization of its estimation error, and demonstrate its robustness by comparing against existing methods. Section 4 extends *TransMission* to the distributed setting. Section 5 validates the theoretical results through simulations and real-world data analysis. Section 6 concludes the paper. Proofs and additional details are provided in the appendix.

Throughout the paper, we use bold uppercase letters for matrices and bold lowercase letters for vectors. For a matrix $A\in R^{m\times n}$, we denote the (i,j)-th element of A by $A_{ij}$. For a p-dimensional vector $x={\left(x_{1},\ldots ,x_{p}\right)}^{⊤}$, we denote its $\ell_{q}$ norm as $\|x{\|}_{q}={\left(\sum_{i=1}^{p}{\left|x_{i}\right|}^{q}\right)}^{1/q}$ for all $q>0,\ell_{0}$-norm as $\|x{\|}_{0}=\#\left\{j:x_{j}\neq 0\right\}$ and $\ell_{\infty }$-norm as $\|x{\|}_{\infty }={\text{max}}_{1\leq j\leq p}x_{j}$. We use ⟨⋅,⋅⟩ to denote the standard Euclidean inner product. Let a∨b denote max{a,b} and a∧b denote min{a,b}. The notation [K] represents the set {1,…,K}. We use $c,c_{0},c_{1},\ldots$ to denote generic constants. Let $a_{n}=O\left(b_{n}\right)$ and $a_{n}≲b_{n}$ denote $\left|a_{n}/b_{n}\right|\leq c$ for some constant c when n is large enough; $a_{n}≍b_{n}$ if $a_{n}=O\left(b_{n}\right)$ and $b_{n}=O\left(a_{n}\right);a_{n}=o\left(b_{n}\right)$ or $b_{n}\gg a_{n}$ if $a_{n}=O\left(c_{n}b_{n}\right)$ for some $c_{n}\to 0$. See Table A.1 for the full notation table.

## 2 Preliminaries

We consider a parameter transfer problem for M-estimation involving one target task and K source tasks. For the target task, we observe a target sample ${𝒮}^{\left(0\right)}={\left\{z_{i}^{\left(0\right)}\right\}}_{i\in \left[n_{T}\right]}$, where each data point $z_{i}^{\left(0\right)}\in 𝒵$ is i.i.d. drawn from a target distribution $P^{\left(0\right)}$. Define $\ell^{\left(0\right)}:𝒵\times R^{p}\to R$ as the target loss function with $\ell^{\left(0\right)}\left(z^{\left(0\right)};\beta \right)$ measures the cost to any parameter $\beta \in R^{p}$ given the target data point $z^{\left(0\right)}$. Our goal is to estimate the unknown target parameter $\beta_{*}^{\left(0\right)}$ defined as

(1) $$\beta_{*}^{\left(0\right)}\in \underset{\beta \in R^{p}}{\text{argmin}}\;E_{z^{\left(0\right)}\sim P^{\left(0\right)}}\left[\ell^{\left(0\right)}\left(z^{\left(0\right)};\beta \right)\right].$$

We focus on a high-dimensional setting where the dimension p is larger than the target sample size $n_{T}$, yet the ground truth $\beta_{*}^{\left(0\right)}$ is a sparse vector with $s≔{\left\|\beta_{*}^{\left(0\right)}\right\|}_{0}$ nonzero elements much smaller than p, i.e. s≪p.

In addition to the target sample, we have access to K source samples ${\left\{{𝒮}^{\left(k\right)}\right\}}_{k\in [K]}$, where each source sample ${𝒮}^{\left(k\right)}$ consists of i.i.d. data points generated from the corresponding source distribution $P^{\left(k\right)}$, which may differ among k∈[K]. Let $\ell^{\left(k\right)}$ be the loss function for each kth source task, we similarly define the source parameter as

(2) $$\beta_{*}^{\left(k\right)}\in \underset{\beta \in R^{p}}{\text{argmin}}\;E_{z^{\left(k\right)}\sim P^{\left(k\right)}}\left[\ell^{\left(k\right)}\left(z^{\left(k\right)};\beta \right)\right],\;k\in \left[K\right].$$

For simplicity, we assume all source samples have the same size $n_{S}$, which is larger than the target sample size $n_{T}$, i.e., $0<n_{T}<n_{S}$.

Our goal is to estimate $\beta_{*}^{\left(0\right)}$ using both the target and source samples in a parameter transfer setting, where the source parameter $\beta_{*}^{\left(k\right)}$ and the target parameter $\beta_{*}^{\left(0\right)}$ share the same feature space and stay in the following parameter space:

(3) $$\Theta \left(s,h\right)≔\left\{\beta_{*}^{\left(0\right)}\in R^{p},\beta_{*}^{\left(k\right)}\in R^{p},k\in \left[K\right]:{\left\|\beta_{*}^{\left(0\right)}\right\|}_{0}\leq s,\underset{k\in \left[K\right]}{\text{max}}{\left\|\beta_{*}^{\left(k\right)}-\beta_{*}^{\left(0\right)}\right\|}_{1}\leq h\right\}.$$

In (3), the sparsity level of the target parameter $\beta_{*}^{\left(0\right)}$ is upper bounded by s, and the informative level of each k-th source tasks is quantified by the $\ell_{1}$-norm of $\delta^{\left(k\right)}≔\beta_{*}^{\left(k\right)}-\beta_{*}^{\left(0\right)}$, and is upper bounded by h≥0. We refer to $\delta^{\left(k\right)}$ as “parameter contrast” or “source-specific signal”. We choose an $\ell_{1}$-sparse constraint for the high-dimensional contrast $\delta^{\left(k\right)}$, as it aligns well with practical applications where parameter shifts typically spread over multiple dimensions but their overall magnitude does not grow too fast (Li et al., 2022; Fan et al., 2023). The results in the paper can be naturally extended to a general $\ell_{q}$-sparse case for q∈[0,1]. The sources are considered informative for parameter transfer if h is relatively small. As in practice h is unknown in practice, one key challenge in parameter transfer is to capture the transferable information when h is small, whereas still being robust when h is large.

Another major challenge in parameter transfer is that though the sources may be transferable with a small h, other distribution characteristics may shift significantly in source populations compared with the target population. To be concrete, we provide some examples of such shifts in a supervised learning setting. Given data $\left(X_{i}^{\left(k\right)},y_{i}^{\left(k\right)}\right)\in 𝒵,i=0,1,\ldots ,n_{k},k=0,1,\ldots ,K$, where k=0 refers to the *target data*, there are three typical *distribution shifts*: (1) *Covariate shift*: potential differences in marginal distributions of $X_{i}^{\left(k\right)}$ among the K+1 populations; (2) *Conditional shift*: potential differences in conditional distributions of $\left(y_{i}^{\left(k\right)}|X_{i}^{\left(k\right)}\right)’\text{s}$, typically exhibited by different parametric model forms between $y_{i}^{\left(k\right)}$ and $X_{i}^{\left(k\right)}$, or different nuisance parameters in the models; (3) *Label shift*: potential differences in marginal distributions of $y_{i}^{\left(k\right)}’\text{s}$. In this paper, we refer to these differences collectively as *distribution shifts*. We want to remark that the conditional shift in our context does not include the differences in parameters of interest - they are the “parameter contrast” that we are modeling. For more intuitive illustration, refer to the following two special examples.

**Example 1 (Normal linear model.)** *Consider the simplest high-dimensional normal linear model:* $y_{i}^{\left(k\right)}=X_{i}^{⊤}\beta_{*}^{\left(k\right)}+\epsilon_{i}^{\left(k\right)}$, *where* $X_{i}^{\left(k\right)}\sim \text{N}\left(0,\Sigma^{\left(k\right)}\right)$ *and* $\epsilon_{i}^{\left(k\right)}\sim \text{N}\left(0,\sigma_{k}^{2}\right)$.

1. **Covariate shift:** *heterogeneity in the covariance matrices across sources and the target, i.e*., $\Sigma^{\left(k\right)}\neq \Sigma^{\left(l\right)}$ *for* l≠k∈{0,…,K}.
2. **Conditional shift:** *heterogeneity in the error variances, i.e*., $\sigma_{k}^{2}\neq \sigma_{l}^{2}$.

**Example 2 (Generalized linear model)** *Consider a high-dimensional generalized linear model defined for each task* k∈{0,1,…,K} *by*

$$y_{i}^{\left(k\right)}\left|X_{i}^{\left(k\right)}\sim P_{Y|X}^{\left(k\right)}\left(y_{i}|X_{i};\beta_{*}^{\left(k\right)}\right)=\rho_{k}\left(y_{i}\right)\;\text{exp}\left(\frac{y_{i}\left(X_{i}^{⊤}\beta_{*}^{\left(k\right)}\right)-\Psi_{k}\left(X_{i}^{⊤}\beta_{*}^{\left(k\right)}\right)}{\phi_{k}}\right)\right.,\;X_{i}^{\left(k\right)}\sim P_{X}^{\left(k\right)},$$

*where* $\Psi_{k}(\cdot )$ *and* $\phi_{k}$ *are univariate functions, with* $\Psi_{k}^{\prime }(\cdot )$ *being the inverse link function, and* $\phi_{k}$ *is the dispersion parameter. This family includes linear, logistic, Poisson, and multinomial regression models as special cases*.

1. ***Covariate shift:*** *heterogeneity in the marginal covariate distributions* $P_{X}^{\left(k\right)}\neq P_{X}^{\left(l\right)}$ *across tasks*.
2. ***Conditional shift:*** *heterogeneity in the conditional response models* $P_{Y|X}^{\left(k\right)}$, *arising from differences in* $\Psi_{k}(\cdot ),\rho_{k}(\cdot )$, *or the dispersion parameter* $\phi_{k}$.

Note that we only explicitly define covariate and conditional shifts in these examples, because due to the parametric model setup and the law of total probability, the **label shift** is automatically driven by covariate shift, conditional shift, or their combination.

## 3 Robust One-step Parameter Transfer

### 3.1 Transfer with Fused-regularizers and Target-data-oriented Constraint

The challenge of parameter transfer lies in tackling the distribution shifts while extracting the commonalities in parameters between source and target samples to estimate the sparse target parameter $\beta_{*}^{\left(0\right)}$. To motivate our method, we first consider a baseline, which estimates $\beta_{*}^{\left(0\right)}$ by pooling together all data and treating them as i.i.d. samples. Specifically, denote the empirical loss on the target and source tasks, respectively as ${ℒ}^{\left(0\right)}\left(\beta^{\left(0\right)}\right)={\left(2n_{T}\right)}^{-1}\sum_{i=1}^{n_{T}}\ell^{\left(0\right)}\left(z_{i}^{\left(0\right)};\beta^{\left(0\right)}\right)$ and ${ℒ}^{\left(k\right)}\left(\beta^{\left(k\right)}\right)={\left(2n_{S}\right)}^{-1}\sum_{i=1}^{n_{S}}\ell^{\left(k\right)}\left(z_{i}^{\left(k\right)};\beta^{\left(k\right)}\right)$. The pooling estimator is defined as

(4) $${\overset{ˆ}{\beta }}_{\text{Pooling}}\in \underset{\beta \in R^{p}}{\text{argmin}}\left\{\sum_{k=0}^{K}\frac{n_{k}}{N}{ℒ}^{\left(k\right)}(\beta )+\lambda \|\beta {\|}_{1}\right\},$$

where $n_{k}=n_{S}$ for $k\in [K],n_{k}=n_{T}$ for $k=0,N=n_{0}+\sum_{k=1}^{K}n_{k}$ is the total sample size and λ is a tuning parameter. Here we incorporate an additional $\ell_{1}$-regularizer to promote a sparse solution. Such an estimator benefits from using a larger sample size to identify the model parameter. However, due to the existence of source-specific signal $\delta^{\left(k\right)}$, such an estimator suffers from a non-negligible bias, and the distribution shifts across sources could further amplify such a bias. To avoid introducing such a bias, one may alternatively estimate the target parameter using solely the target data via solving:

(5) $${\overset{ˆ}{\beta }}_{T}^{\left(0\right)}\in \underset{\beta \in R^{p}}{\text{argmin}}\left\{{ℒ}^{\left(0\right)}(\beta )+\lambda \|\beta {\|}_{1}\right\}.$$

However, with such a single-task estimator, the source dataset will under no circumstance be helpful, even when the distributions are identical. It is therefore critical to find a method that can capture the transferable parameter knowledge, whereas being robust to the distribution shifts to improve the estimation of $\beta_{*}^{\left(0\right)}$. To achieve such a goal, we introduce a new transfer learning framework for M-estimation with fused regularization, abbreviated as *TransMission*, which estimates $\beta_{*}^{\left(0\right)}$ by solving the following problem:

(6a) $$\underset{\beta^{\left(0\right)}\in {𝒞}_{T},\beta^{\left(k\right)}\in R^{p},k\in [K]}{\text{min}}\left\{\sum_{k=0}^{K}\frac{n_{k}}{N}{ℒ}^{\left(k\right)}\left(\beta^{\left(k\right)}\right)+\lambda_{0}{\left\|\beta^{\left(0\right)}\right\|}_{1}+\lambda_{1}\sum_{k=1}^{K}{\left\|\beta^{\left(k\right)}-\beta^{\left(0\right)}\right\|}_{1}\right\},$$

(6b) $$\text{with}\;{𝒞}_{T}=\left\{\beta^{\left(0\right)}\in R^{p}:{\left\|\nabla {ℒ}^{\left(0\right)}\left(\beta^{\left(0\right)}\right)\right\|}_{\infty }\leq \lambda_{T},{\left\|\beta^{\left(0\right)}\right\|}_{1}\leq {\left\|{\overset{ˆ}{\beta }}_{T}^{\left(0\right)}\right\|}_{1}+\kappa_{T}\right\},$$

where $N=Kn_{S}+n_{T}$ is the total sample size, $\lambda_{0}$ and $\lambda_{1}$ are the tuning parameters, and ${𝒞}_{T}$ is a target-data oriented constraint that will be discussed shortly. In (6a), the first term measures the average fitness of the models ${\left\{\beta^{\left(k\right)}\right\}}_{k=0}^{K}$, while the regularization terms simultaneously promote the sparsity of $\beta^{\left(0\right)}$ and capture the shared knowledge between $\beta^{\left(0\right)}$ and $\beta^{\left(k\right)}$ penalizing their $\ell_{1}$ difference. The non-transferable source-specific signal $\delta^{\left(k\right)}=\beta_{*}^{\left(k\right)}-\beta_{*}^{\left(0\right)}$ is explicitly estimated and filtered out. Notice that unlike the pooling estimator ${\overset{ˆ}{\beta }}_{\text{Pooling}}$ which transfers knowledge from the loss level, the *TransMission* estimator achieves the parameter transfer from the parameter level: transferable knowledge is first encoded into the parameter $\beta^{\left(k\right)}$ and then passed to the estimation of target parameter $\beta^{\left(0\right)}$. This allows *TransMission* to be robust to a variety of distribution shifts when transferring the parameter knowledge from heterogeneous sources.

In (6b), we introduce an additional constraint ${𝒞}_{T}$ on $\beta^{\left(0\right)}$, where $\lambda_{T}$ and $\kappa_{T}$ are tuning parameters and ${\overset{ˆ}{\beta }}_{T}^{\left(0\right)}$ is the single-task estimator defined in (5). The first term ${\left\|\nabla {ℒ}^{\left(0\right)}\left(\beta^{\left(0\right)}\right)\right\|}_{\infty }\leq \lambda_{T}$ can be understood as a “confidence set” that summarizes the information on $\beta^{\left(0\right)}$ provided by the target data (Fan, 2013). The second term, ${\left\|\beta^{\left(0\right)}\right\|}_{1}\leq {\left\|{\overset{ˆ}{\beta }}_{T}^{\left(0\right)}\right\|}_{1}+\kappa_{T}$, constrains the size of the *TransMission* estimator relative to the single-task estimator. This constraint promotes sparsity within a feasible search space informed by the target data. Ideally, the sparsity level should be controlled by the true target parameter, i.e., ${\left\|\beta^{\left(0\right)}\right\|}_{1}\leq {\left\|\beta_{*}^{\left(0\right)}\right\|}_{1}$ (Fan, 2013; Hastie et al., 2015). However, since ${\left\|\beta_{*}^{\left(0\right)}\right\|}_{1}$ is unknown in practice, so we propose to approximate it with the consistent estimator ${\left\|{\overset{ˆ}{\beta }}_{T}^{\left(0\right)}\right\|}_{1}$, and incorporate an additional slack term $\kappa_{T}$ to account for the noise in the single-task estimator. Overall, the constraint ${𝒞}_{T}$ narrows down the search space for $\beta^{\left(0\right)}$ using the target samples, and the objective function further selects within these candidates a good one assisted by the side information learned from the source samples. Thus, the resulting estimator is ensured to remain within the local neighborhood of the true target parameter, eliminating the need for any further debiasing steps while performing at least as well as the estimator based solely on the target sample.

In practice, implementing TransMission requires solving a single fused regularization problem together with a target-oriented constraint. At a high level, the joint optimization in (6a) can be reformulated as a standard $\ell_{1}$-penalized regression through an appropriate reparameterization, allowing the use of off-the-shelf solvers. We then employ an adaptive tuning parameter selection strategy to ensure the solution satisfies the constraint (6b). Guided by the theoretical analysis, we further reduce the four tuning parameters $\lambda_{0},\lambda_{1},\lambda_{T}$, and $\kappa_{T}$ to two tuning parameters, both selected using cross-validation and a small grid search. A complete implementation guide is provided in Appendix A.2.

**Algorithm 1:** TransMission

**Input:** target sample ${𝒮}^{\left(0\right)}$, source samples ${\left\{{𝒮}^{\left(k\right)}\right\}}_{k=1}^{K}$, tuning parameters $\lambda_{0},\lambda_{1},\lambda_{T},\kappa_{T}$.
**Output:** the estimated target parameter ${\hat{\beta }}^{\left(0\right)}$.
1. Compute ${\hat{\beta }}_{T}^{\left(0\right)}$ by solving ${\text{min}}_{\beta \in {\mathbb{R}}^{p}}\left\{{ℒ}^{\left(0\right)}(\beta )+\lambda_{T}\|\beta {\|}_{1}\right\}$.
2. Compute (${\hat{\beta }}^{\left(0\right)},{\hat{\beta }}^{\left(1\right)},\ldots ,{\hat{\beta }}^{\left(K\right)}$) by solving $$\underset{\beta^{\left(0\right)}\in {\mathbb{R}}^{p},{\left\{\beta^{\left(k\right)}\right\}}_{k=1}^{K}\in {\left({\mathbb{R}}^{p}\right)}^{K}}{\text{min}}\sum_{k=0}^{K}\frac{n_{k}}{N}{ℒ}^{\left(k\right)}\left(\beta^{\left(k\right)}\right)+\lambda_{0}\|\beta^{\left(0\right)}{\|}_{1}+\lambda_{1}\sum_{k=1}^{K}\|\beta^{\left(k\right)}-\beta^{\left(0\right)}{\|}_{1},\;\text{with}\;{𝒞}_{T}=\left\{\beta^{\left(0\right)}\in {\mathbb{R}}^{p}:\|\nabla {ℒ}^{\left(0\right)}\left(\beta^{\left(0\right)}\right){\|}_{\infty }\leq \lambda_{T},\|\beta^{\left(0\right)}{\|}_{1}\leq \|{\hat{\beta }}_{T}^{\left(0\right)}{\|}_{1}+\kappa_{T}\right\}.$$
3. Output ${\hat{\beta }}^{\left(0\right)}$.

### 3.2 Theoretical Properties

We now demonstrate the robustness of the proposed *TransMission* estimator under distribution shifts by establishing and discussing its statistical convergence rate. To facilitate the analysis, we introduce some regularity conditions on the loss function. Since standard strong convexity and smoothness assumptions are overly restrictive in high-dimensional settings, we instead impose their relaxations that are widely adopted in the high-dimensional analysis of M-estimators (Negahban et al., 2012).

**Assumption 1 (Restricted Strong Convexity and Smoothness)** *For any* $k=0,\ldots ,K,\beta ,𝚫\in R^{p}$*, there exists constants* $\alpha_{k},\beta_{k},\tau_{k},\gamma_{k}>0$ *such that*

$$\left\langle \nabla {ℒ}^{\left(k\right)}(\beta +𝚫)-\nabla {ℒ}^{\left(k\right)}(\beta ),𝚫\right\rangle \geq \alpha_{k}\|𝚫{\|}_{2}^{2}-\tau_{k}\frac{\text{log}\;p}{n_{k}}\|𝚫{\|}_{1}^{2},$$

$$\left\langle \nabla {ℒ}^{\left(k\right)}(\beta +𝚫)-\nabla {ℒ}^{\left(k\right)}(\beta ),𝚫\right\rangle \leq \beta_{k}\|𝚫{\|}_{2}^{2}+\gamma_{k}\frac{\text{log}\;p}{n_{k}}\|𝚫{\|}_{1}^{2},$$

*with probability larger than* $1-c_{1}\;\text{exp}\left(-c_{2}n_{k}-c_{3}\;\text{log}\;p\right)$*, where* $n_{k}=n_{S}$ *for* $k\in [K],n_{k}=n_{T}$ *for* k=0, *and* $c_{1},c_{2},c_{3}$ *are generic constants*.

**Remark 1** *For linear regression models specified in Example 1, Assumption 1 holds with a broad class of anisotropic random design matrices* (Rudelson and Zhou, 2012). *For the broad class of generalized linear models specified in Example 2, Assumption 1 holds for* $\beta ,𝚫\in B_{2}(R)$ *for some large constant* R (Agarwal et al., 2010). *Here* $B_{2}(R)≔\left\{\beta \in R^{p}\right.:\left.\|\beta {\|}_{2}\leq R\right\}$ *denotes the Euclidean* $\ell_{2}$-*ball of radius* R>0. *The restriction set* $B_{2}(R)$ *is essential because for some generalized linear models, the Hessian function* $\Psi_{k}^{\prime \prime }(\cdot )$ *approaches zero as its argument diverges. For these models, our results hold with the restriction set imposed on the solution and parameter space. See a detailed discussion in* Agarwal et al. (2010); Loh and Wainwright (2011, 2013).

In the following, we define $\alpha_{\text{min}}={\text{min}}_{0\leq k\leq K}\alpha_{k},\alpha_{\text{max}}={\text{max}}_{0\leq k\leq K}\alpha_{k}$ and $\beta_{\text{max}}={\text{max}}_{0\leq k\leq K}\beta_{k}$, with $\alpha_{k}$ and $\beta_{k}$ being the constants specified in Assumption 1. Notice that in Assumption 1, we impose regularity conditions on each empirical loss function ${ℒ}^{\left(k\right)}$ individually but not any on their differences. This flexibility accounts for potential distribution shifts across tasks. Next, we state a theorem that shows with a proper choice of tuning parameters and sufficiently large sample size, the *TransMission* estimator ${\overset{ˆ}{\beta }}^{\left(0\right)}$ is guaranteed to be close to the target parameter $\beta_{*}^{\left(0\right)}$ under distribution shifts. The theorem is deterministic in nature, with corresponding probabilistic results provided in the subsequent corollaries.

**Theorem 1** *Suppose Assumption 1 holds and the true parameters are in* Θ(s,h) *defined in* (3). *For TransMission problem* (6), *consider any choice of tuning parameters such that*, $\lambda_{T}\geq 2{\left\|\nabla {ℒ}^{\left(0\right)}\left(\beta_{*}^{\left(0\right)}\right)\right\|}_{\infty },\kappa_{T}=24s\lambda_{T}/\alpha_{0}$,

$$\lambda_{0}\geq 2{\left\|\sum_{k=0}^{K}\frac{n_{k}}{N}\nabla {ℒ}^{\left(k\right)}\left(\beta_{*}^{\left(k\right)}\right)\right\|}_{\infty },\lambda_{1}\geq \frac{2}{K}\underset{k\in \left[K\right]}{\text{max}}{\left\|\nabla {ℒ}^{\left(k\right)}\left(\beta_{*}^{\left(k\right)}\right)\right\|}_{\infty }\vee \left(\frac{4\alpha_{\text{max}}}{\gamma_{0}K}\lambda_{T}\right).$$

*Suppose* $n_{S}>n_{T}>\left[\frac{C_{0}\left(2\lambda_{0}^{2}s+K\lambda_{1}h/\gamma_{0}\right)}{\left(K\lambda_{1}^{2}\right)\wedge \lambda_{0}^{2}}+C_{1}s\right]\text{log}p$, *then any solution* ${\overset{ˆ}{\beta }}^{\left(0\right)}$ *to the problem satisfies*

(7) $${\left\|{\overset{ˆ}{\beta }}^{\left(0\right)}-\beta_{*}^{\left(0\right)}\right\|}_{2}^{2}\leq \left(C_{2}\lambda_{0}^{2}s+C_{3}K\lambda_{1}h\right)\wedge \left(C_{4}\lambda_{T}^{2}s\right),$$

*where* $C_{0}=\frac{64\left(\alpha_{\text{max}}\tau_{0}+\beta_{\text{max}}\gamma_{0}\right)}{\alpha_{\text{min}}^{2}},C_{1}=\frac{64\left(\tau_{0}^{2}+\alpha_{0}\tau_{0}\right)}{\alpha_{0}^{2}},C_{2}=\frac{9\gamma_{0}^{2}}{\alpha_{\text{min}}^{4}},C_{3}=\frac{12\gamma_{0}}{\alpha_{\text{min}}^{2}}$ *and* $C_{4}=\frac{128}{\alpha_{0}^{2}}$.

The convergence rate established in (7) is given by the minimum of two terms, with the first term contributed by solving the objective (6a) and the second term coming from the constraint ${𝒞}_{T}$ in (6b). In the first term, $C_{2}\lambda_{0}^{2}s$ reflects the rate of leveraging both target and source samples for estimating the s-sparse target parameter $\beta_{*}^{\left(0\right)}$, whereas $C_{3}K\lambda_{1}h$ is the cost of estimating the source-target contrasts ${\left\{\delta^{\left(k\right)}\right\}}_{k\in [K]}$, each of which is of magnitude h. The second term, $C_{4}\lambda_{T}^{2}s$, corresponds to the rate obtained when using only the target sample for estimation. The bound shows that the *TransMission* estimator benefits from parameter transfer when the source tasks provide useful information, i.e., when h is small, and performs no worse than the single-task estimator when the source tasks are not beneficial. Further elaboration on this point will be given by Corollary 1 with a specific choice of tuning parameters. Notably, the results hold under a variety of distribution shifts, provided that the regularity conditions in Assumption 1 on each of the empirical loss functions ${ℒ}^{\left(k\right)}$ are satisfied.

To better interpret the result, we provide the probabilistic counterpart of Theorem 1 in the sequel, along with the choice of tuning parameters. We first introduce an additional assumption on the concentration property of loss functions.

**Assumption 2 (Concentration)** ${\left\{\nabla \ell^{\left(k\right)}\left(z_{i}^{\left(k\right)};\beta_{*}^{\left(k\right)}\right)\right\}}_{k=0,\ldots ,K,i\in \left[n_{k}\right]}$ *are independent random vectors and for any* j∈[p], *there are universal constants* $c_{1},c_{2}$ *such that*

$$P\left(\left|\nabla_{j}\ell^{\left(k\right)}\left(z_{i}^{\left(k\right)};\beta_{*}^{\left(k\right)}\right)\right|\geq t\right)\leq 2\;\text{exp}\left[-c_{2}t^{2}/\sigma^{2}\right],\;\text{for all}\;t>c_{1},$$

*where* σ *is a constant that is bounded above*.

Assumption 2 requires the sample gradient evaluated at the true parameter to exhibit sub-Gaussian tails, a condition satisfied by various well-known models, including the generalized linear models discussed in Example 1, when the design matrix is sub-Gaussian (Agarwal et al., 2010). It’s worth noting that Assumption 2, along with Assumption 1, is not the weakest possible for the method’s broad application. We adopt these conditions to align with the literature, facilitating a direct comparison between the theoretical results of *TransMission* and existing methods. With this additional assumption, we are ready to establish the convergence rate of ${\overset{ˆ}{\beta }}^{\left(0\right)}$ that holds with high probability.

**Corollary 1** *Suppose Assumption 1 and 2 hold and the true parameters are in* Θ(s,h) *defined in* (3). *Suppose* $n_{S}>n_{T}\gg Ks\;\text{log}\;p$, *if we choose*

$$\lambda_{0}≍\left[{\left(\frac{h^{2}\;\text{log}\;p}{s^{2}n_{T}}\right)}^{1/4}+{\left(\frac{\text{log}\;p}{N}\right)}^{1/2}\right],\;\lambda_{1}≍\frac{n_{S}}{N}{\left(\frac{\text{log}\;p}{n_{T}}\right)}^{1/2},\;\text{and}\;\lambda_{T}≍{\left(\frac{\text{log}\;p}{n_{T}}\right)}^{1/2},$$

*then the solution of the TransMission problem* (6) *satisfies*

(8) $${\left\|{\overset{ˆ}{\beta }}^{\left(0\right)}-\beta_{*}^{\left(0\right)}\right\|}_{2}^{2}≲\frac{s\;\text{log}\;p}{N}+\left(h\sqrt{\frac{\text{log}\;p}{n_{T}}}\right)\wedge \left(\frac{s\;\text{log}\;p}{n_{T}}\right),$$

*with probability larger than* $1-c_{1}\;\text{exp}\left(-c_{2}\;\text{log}\;p\right)$.

The first term slogp/N in (8) is the rate of estimating an s-sparse parameter $\beta_{*}^{\left(0\right)}$ based on N i.i.d. samples. This term reveals the benefit of using both the source and target datasets for estimating the target parameter $\beta_{*}^{\left(0\right)}$. The term $h\sqrt{\text{log}\;p/n_{T}}$ accounts for the rate of estimating $\delta^{\left(k\right)}$ unique to each source task and thus is limited by the target dataset size $n_{T}$. The term $s\;\text{log}\;p/n_{T}$ is the minimax optimal rate for estimating $\beta_{*}^{\left(0\right)}$ using only the target data when s≪p (Raskutti et al., 2011). Taken together, the upper bound reveals that *TransMission* has a sharper convergence rate than estimators using the target sample alone when $h≲s\sqrt{\text{log}\;p/n_{T}}$, and always performs no worse than these estimators when $h≳s\sqrt{\text{log}\;p/n_{T}}$. Notably, the rate depends only on the magnitude of the parameter contrast, i.e, h, and is unaffected by other forms of distribution shift. We explore this further in the next section.

### 3.3 Understanding the Robustness of the TransMission Method

In this section, we discuss the robustness of the *TransMission* method compared to the two-step method proposed in Li et al. (2022) and Tian and Feng (2023). While both methods aim to address the high-dimensional parameter transfer problem, we take a significantly different approach to achieve robustness to distribution shifts and guarantee no negative transfer. To understand this, we first demonstrate why the pooling estimator ${\overset{ˆ}{\beta }}_{\text{Pooling}}$, defined in (4), will fail under distribution shifts. Define the population counterpart of the first-step pooling estimator as $\beta_{\text{Pooling}}\in {\text{argmin}}_{\beta }\sum_{k=0}^{K}\frac{n_{k}}{N}E\left[{ℒ}^{\left(k\right)}(\beta )\right]$. By the optimality of $\beta_{\text{Pooling}}$ and the definition of $\beta_{*}^{\left(k\right)}$ and assuming that ${ℒ}^{\left(k\right)}$ is second order differentiable, we have

$$\sum_{k=0}^{K}\frac{n_{k}}{N}E\left(\nabla {ℒ}^{\left(k\right)}\left(\beta_{\text{Pooling}}\right)-\nabla {ℒ}^{\left(k\right)}\left(\beta_{*}^{\left(k\right)}\right)\right)=\sum_{k=0}^{K}\frac{n_{k}}{N}E\left(\nabla^{2}{ℒ}_{\text{Int}}^{\left(k\right)}\left(\beta_{\text{Pooling}}-\beta_{*}^{\left(k\right)}\right)\right)=0,$$

where we define $\nabla^{2}{ℒ}_{\text{Int}}^{\left(k\right)}≔\nabla^{2}{ℒ}^{\left(k\right)}\left(\beta_{\text{Int}}^{\left(k\right)}\right)$ with $\beta_{\text{Int}}^{\left(k\right)}$ being some intermediate point between $\beta_{\text{Pooling}}$ and $\beta_{*}^{\left(k\right)}$. Further assuming that the Hessian matrix is invertible, we can compute the asymptotic bias of the pooling estimator as

(9) $$\delta_{\text{Pooling}}≔\beta_{\text{Pooling}}-\beta_{*}^{\left(0\right)}={\left[\sum_{k=0}^{K}\frac{n_{k}}{N}E\left(\nabla^{2}{ℒ}_{\text{Int}}^{\left(k\right)}\right)\right]}^{-1}\sum_{k=1}^{K}\frac{n_{k}}{N}E\left(\nabla^{2}{ℒ}_{\text{Int}}^{\left(k\right)}\right)\delta^{\left(k\right)},$$

where recall $\delta^{\left(k\right)}=\beta_{*}^{\left(k\right)}-\beta_{*}^{\left(0\right)}$ is the source-specific signal.

Recall that in the parameter space Θ(s,h), we assumed that $\delta^{\left(k\right)}$ is $\ell_{1}$-sparse in the sense that ${\left\|\delta^{\left(k\right)}\right\|}_{1}\leq h$ for all k∈[K]. Therefore, in a homogeneous setting where the expected Hessian $E\left(\nabla^{2}{ℒ}_{\text{Int}}^{\left(k\right)}\right)$ are the same across sources, $\delta_{\text{Pooling}}$ is a convex combination of $\delta^{\left(k\right)}\text{s}$ with ${\left\|\delta_{\text{Pooling}}\right\|}_{1}\leq h$. However, under a distribution shift setting where the expected Hessian can vary arbitrarily across different sources, the sparsity pattern of $\delta_{\text{Pooling}}$ could be destroyed when multiplying $\delta^{\left(k\right)}\text{s}$ by the expected Hessian matrix before combining. Consequently, the pooling bias $\delta_{\text{Pooling}}$ may no longer have a bounded $\ell_{1}$ norm, leading to a deterioration in the accuracy of sparse estimation.

The two-step methods proposed in Li et al. (2022) and Tian and Feng (2023) achieve parameter transfer by correcting the bias of $\delta_{\text{Pooling}}$ via regularized regression using solely the target data. As shown in Table 1, these two-step estimator based on such a pooling estimator leads to a rate with an additional term $C_{\Sigma }$ due to the distribution shifts, where

(10) $$C_{\Sigma }≔1+\underset{j\leq p}{\text{max}\;}\underset{k}{\text{max}}{\left\|e_{j}^{⊤}\left(E\left(\nabla^{2}{ℒ}_{\text{Int}}^{\left(k\right)}\right)-E\left(\nabla^{2}{ℒ}_{\text{Int}}^{\left(0\right)}\right)\right){\left(\sum_{k=1}^{K}\frac{1}{K}E\left(\nabla^{2}{ℒ}_{\text{Int}}^{\left(k\right)}\right)\right)}^{-1}\right\|}_{1}.$$

with $e_{j}\in R^{p}$ being the jth canonical basis vector. Such a factor $C_{\Sigma }$ grows with the distribution shifts across tasks and can diverge in the rate of $O(\sqrt{p})$ even in the linear regression setting (He et al., 2024). Therefore their method suffers significantly from the high-dimensional distribution shifts, while *TransMission* is very robust to such shifts.

**Remark 2** *Here we further clarify the distribution shifts from the perspective of parametric models. In statistical estimation*, $E\left(\nabla^{2}{ℒ}_{\text{Int}}^{\left(k\right)}\right)$ *in* (10) *is closely related to the Fisher information matrix. The heterogeneity in Fisher information matrix can be understood along three shift types: (i)* covariate shift*, (ii)* conditional shift*, and (iii) the* label shift *induced by the first two. For example, in the linear model of Example 1, the Fisher information matrix reduces (up to scale) to the covariance* ${𝚺}^{\left(k\right)}=\frac{1}{n_{k}}E\left({\left(X^{\left(k\right)}\right)}^{⊤}X^{\left(k\right)}\right)$, *so heterogeneity in the Fisher information matrix directly corresponds to covariate shift, i.e*., $P_{X}^{\left(k\right)}\neq P_{X}^{\left(l\right)}$ *for* l≠k∈{0,…,K}; *in the generalized linear models of Example 2, the Fisher information matrix is proportional to* $\frac{1}{n_{k}}E\left({\left(X^{\left(k\right)}\right)}^{⊤}W_{k,\text{Int}}^{2}X^{\left(k\right)}\right)$ *with diagonal weights* ${\left(W_{k,\text{Int}}\right)}_{ii}=\phi_{k}^{-1/2}\Psi_{k}^{\prime \prime }{\left({\left(X_{i}^{\left(k\right)}\right)}^{⊤}\beta_{\text{Int}}^{\left(k\right)}\right)}^{1/2}$; *so heterogeneity in Fisher information matrix can arise from heterogeneity in the variance function* $\Psi_{k}^{\prime \prime }$ *or the dispersion* $\phi_{k}$, *which corresponds to the conditional shift* $P_{y|X}^{\left(k\right)}\neq P_{y|X}^{\left(l\right)}$. *Since the marginal label distribution depends on both* $P_{X}^{\left(k\right)}$ *and* $P_{y|X}^{\left(k\right)}$, *label shift follows whenever either of these distributions changes. The factor* $C_{\Sigma }$ *in* (10) *quantifies discrepancies induces from the above shifts*.

**Remark 3** *We would like to clarify that our goal with the proposed TransMission estimator is not to explicitly tackle each type of distribution shifts individually, but to preserve effective and robust parameter transfer in the presence of distribution shifts. Concretely, of true interest is capturing the “parameter contrast”* $\delta^{\left(k\right)}=\beta^{\left(k\right)}-\beta^{\left(0\right)}$, *while covariate and conditional distributions are allowed to vary across tasks. Such a robustness follows from the joint training with fused-regularization design in* (6a), *which achieves parameter transfer from the parameter level rather than loss level. As the loss functions are jointly considered in the objective function, with their respective parameters, and thus the losses (and hence the entire parametric models) are allowed to be different*.

Another advantage of our method is about preventing negative transfer, i.e., ensuring that incorporating source data does not degrade the estimation accuracy of the target parameter when h is large. As shown in Table 1, Li et al. (2022) proposes to perform an additional source detection step to address this negative transfer issue. However, such an additional detection step comes with a cost of order $O\left(\text{log}\;K/n_{T}\right)$, therefore the overall convergence rate is always no faster than $O\left(\text{log}\;K/n_{T}\right)$ even if h is small. In contrast, *TransMission* prevents negative transfer through a target data-oriented constraint during parameter transfer, without sacrificing the statistical convergence rate in the transferable regime, although it may incur additional computational cost due to an extra pilot estimator construction and hyperparameter tuning step.

**Remark 4** *Several concurrent works, including TransHDGLM (*Li et al., 2024*), STRIFLE (*Cai et al., 2025*), TransFusion (*He et al., 2024*), and TransQR (*Bai et al., 2024*) have also demonstrated robustness in high-dimensional parameter transfer. However, they either fail to account for negative transfer or incur non-negligible costs, typically of order* $O\left(1/n_{T}\right)$*, to prevent the negative transfer*.

### 3.4 Sharper Convergence Rate and Minimax Optimality

We now show that with a more careful choice of tuning parameters, the *TransMission* estimator could achieve a faster convergence rate, and can match the existing lower bound, showing the advantage of the proposed method and the tightness of our bound.

**Corollary 2** *Suppose Assumption 1, 2 hold and the true parameters are in* Θ(s,h) *defined in* (3). *If* $n_{S}>n_{T}\gg Ks\;\text{log}\;p$, *and the tuning parameters are chosen as outlined in* Appendix A.3, *then the solution of the TransMission problem* (6) *satisfies*

$${\left\|{\overset{ˆ}{\beta }}^{\left(0\right)}-\beta_{*}^{\left(0\right)}\right\|}_{2}^{2}≲\frac{s\;\text{log}\;p}{N}+\left(\sqrt{\frac{\text{log}\;p}{n_{T}}}h\right)\wedge \left(C_{\Sigma }^{2}h^{2}\right)\wedge \frac{s\;\text{log}\;p}{n_{T}},$$

*with probability larger than* $1-c_{1}\;\text{exp}\left(-c_{2}\;\text{log}\;p\right)$.

Compared to Corollary 1, Corollary 2 establishes a sharper convergence rate by taking minimum over an additional term, $C_{\Sigma }^{2}h^{2}$, in the second part of the bound. Recall that $C_{\Sigma }$, defined in (10), measures the degree of distribution shifts. This term enables a faster rate of convergence when $h≲\left(C_{\Sigma }^{-2}\sqrt{\text{log}\;p/n_{T}}\right)\wedge \left(C_{\Sigma }^{-1}\sqrt{s\;\text{log}\;p/n_{T}}\right)$, i.e., when the source and target distributions are highly similar. Appendix A.3 provides a detailed discussion of how such a tighter bound is achieved via a more careful choice of tuning parameters.

The upper bound established in Corollary 2 recovers the existing minimax lower bounds in the homogeneous setting. Specifically, prior works (Li et al., 2022; Tian and Feng, 2023) have shown that in linear regression and generalized linear models with a homogeneous design ($C_{\Sigma }=1$), the $\ell_{2}$-estimation error for $\beta_{*}^{\left(0\right)}$ has the lower bound

$$\underset{{\overset{ˇ}{\beta }}^{\left(0\right)}}{\text{inf}}\underset{\beta_{*}^{\left(0\right)}\in \Theta (s,h)}{\text{sup}}{\left\|{\overset{ˇ}{\beta }}^{\left(0\right)}-\beta_{*}^{\left(0\right)}\right\|}_{2}^{2}≳\frac{s\;\text{log}\;p}{N}+\left(h\sqrt{\frac{\text{log}\;p}{n_{T}}}\right)\wedge h^{2}\wedge \frac{s\;\text{log}\;p}{n_{T}},$$

with probability at least 1/2. In this setting, one can verify that $C_{\Sigma }=1$, and our upper bound in Corollary 2 matches the minimax lower bound, showing the minimax optimality of *TransMission* under the conditions outlined in Corollary 2. Notably, even in this homogeneous setting ($C_{\Sigma }=1$), our method achieves the minimax lower bound under significantly weaker conditions on h compared to existing works (Li et al., 2022; Tian and Feng, 2023; Li et al., 2024). For example, TransGLM requires $h≲s\sqrt{\text{log}\;p/n_{T}}$ and $h\ll \sqrt{n_{T}/\text{log}\;p}$ to attain the minimax rate, which requires h to be sufficiently small and the target sample $n_{T}$ to be neither too large nor too small. On the other hand, we only assume $h\ll \sqrt{n_{T}/\left(K^{2}\;\text{log}\;p\right)}$. Given that the high-dimensional analysis typically requires $n_{T}\gg s\;\text{log}\;p$, our assumption is much less restrictive on h compared to existing work.

Moreover, beyond the homogeneous case, our method remains robust to a broad class of distribution shifts, achieving a faster convergence in heterogeneous settings.

## 4 Distributed Parameter Transfer With Source Estimators

### 4.1 Distributed TransMission

In this section, we consider a distributed setting where the target and K source datasets are stored across different institutes or organizations, and direct data sharing is forbidden due to privacy and proprietary concerns. In such a setting, raw data cannot be pooled and multi-round communication-based distributed learning is often impractical due to the high dimensionality of model parameters. This motivates the development of a distributed version of *TransMission*, termed *D-TransMission*, which estimates the target parameter $\beta_{*}^{\left(0\right)}$ using a *single round* of communication based on pre-fitted source parameters.

Our method integrates the idea of divide-and-conquer into the *TransMission* method described in Section 3, aiming to achieve a comparable estimation error as ${\overset{ˆ}{\beta }}^{\left(0\right)}$ using only one-shot communication. Note that although divide-and-conquer has been well-studied for i.i.d. data (Lee et al., 2017), its application and analysis for high-dimensional parameter transfer is largely unexplored. Specifically, in *D-TransMission*, we let each source node k compute an estimator ${\tilde{\beta }}^{\left(k\right)}\in R^{p}$ locally based on source dataset ($X^{\left(k\right)},y^{\left(k\right)}$) and send it to the target node. The target node then aggregates them with its own dataset ($X^{\left(0\right)},y^{\left(0\right)}$) via solving the following joint learning problem:

(11a) $$\underset{\beta^{\left(0\right)}\in {𝒞}_{T},\beta^{\left(k\right)}\in R^{p},k\in [K]}{\text{min}}\left\{\frac{n_{T}}{N}{ℒ}^{\left(0\right)}\left(\beta^{\left(0\right)}\right)+\frac{n_{S}^{2}}{2N}\sum_{k=1}^{K}{\left\|{\tilde{\beta }}^{\left(k\right)}-\beta^{\left(k\right)}\right\|}_{2}^{2}+\lambda_{0}{\left\|\beta^{\left(0\right)}\right\|}_{1}+\sum_{k=1}^{K}\lambda_{1}{\left\|\beta^{\left(k\right)}-\beta^{\left(0\right)}\right\|}_{1}\right\},$$

(11b) $$\text{with}\;{𝒞}_{T}=\left\{\beta^{\left(0\right)}\in R^{p}:{\left\|\nabla {ℒ}^{\left(0\right)}\left(\beta^{\left(0\right)}\right)\right\|}_{\infty }\leq \lambda_{T},{\left\|\beta^{\left(0\right)}\right\|}_{1}\leq {\left\|{\overset{ˆ}{\beta }}_{T}^{\left(0\right)}\right\|}_{1}+\kappa_{T}\right\},$$

Compared to its centralized counterpart, *D-TransMission* replaces the source empirical loss ${ℒ}^{\left(k\right)}(\cdot )$ with the mean squared error from the source parameter estimator ${\tilde{\beta }}^{\left(k\right)}$ as a measure of model fitness. Such a modification makes *D-TransMission* no longer require transmitting the raw source data for transfer learning, reducing the communication cost from $O\left(n_{S}Kp\right)$ to O(Kp), which is a substantial improvement when either $n_{S}$ or p is large. Algorithm 2 provides a pseudocode of the *D-TransMission* method.

**Algorithm 2:** D-TransMission

**Input:** target sample ${𝒮}^{\left(0\right)}$, source parameter estimators ${\left\{{\tilde{\beta }}^{\left(k\right)}\right\}}_{k=1}^{K}$, tuning parameters $\lambda_{0},\lambda_{1},\lambda_{T},\kappa_{T}$.
**Output:** the estimated target parameter ${\hat{\beta }}_{D}^{\left(0\right)}$.
1. Compute ${\hat{\beta }}_{T}^{\left(0\right)}$ by solving ${\text{min}}_{\beta \in {\mathbb{R}}^{p}}\left\{{ℒ}^{\left(0\right)}(\beta )+\lambda_{T}\|\beta {\|}_{1}\right\}$.
2. Compute (${\hat{\beta }}_{D}^{\left(0\right)},{\hat{\beta }}_{D}^{\left(1\right)},\ldots ,{\hat{\beta }}_{D}^{\left(K\right)}$) by solving $$\underset{\beta^{\left(0\right)}\in {\mathbb{R}}^{p},{\left\{\beta^{\left(k\right)}\right\}}_{k=1}^{K}\in {\left({\mathbb{R}}^{p}\right)}^{K}}{\text{min}}\frac{n_{T}}{N}{ℒ}^{\left(0\right)}\left(\beta^{\left(0\right)}\right)+\frac{n_{S}^{2}}{2N}\sum_{k=1}^{K}\|{\tilde{\beta }}^{\left(k\right)}-\beta^{\left(k\right)}{\|}_{2}^{2}+\lambda_{0}\|\beta^{\left(0\right)}{\|}_{1}+\lambda_{1}\sum_{k=1}^{K}\|\beta^{\left(k\right)}-\beta^{\left(0\right)}{\|}_{1},\;\text{with}\;{𝒞}_{T}=\left\{\beta^{\left(0\right)}\in {\mathbb{R}}^{p}:\|\nabla {ℒ}^{\left(0\right)}\left(\beta^{\left(0\right)}\right){\|}_{\infty }\leq \lambda_{T},\|\beta^{\left(0\right)}{\|}_{1}\leq \|{\hat{\beta }}_{T}^{\left(0\right)}{\|}_{1}+\kappa_{T}\right\}.$$
3. Output ${\hat{\beta }}_{D}^{\left(0\right)}$.

Next, we specify how to choose ${\tilde{\beta }}^{\left(k\right)}$ to preserve the convergence rate of *TransMission*. Under the parameter space Θ(s,h), the $\beta_{*}^{\left(k\right)}’\text{s}$ are also sparse. Therefore, a natural choice of ${\tilde{\beta }}^{\left(k\right)}$ is the $\ell_{1}$-regularized estimator computed purely based on source dataset given by:

(12) $${\overset{ˆ}{\beta }}_{L}^{\left(k\right)}\in \underset{\beta }{\text{argmin}}\left\{{ℒ}^{\left(k\right)}(\beta )+{\tilde{\lambda }}_{k}\|\beta {\|}_{1}\right\}.$$

However, it is well known that the regularization term introduces a bias in ${\overset{ˆ}{\beta }}_{L}^{\left(k\right)}$, and aggregating the local ${\overset{ˆ}{\beta }}_{L}^{\left(k\right)}\text{s}$ can only reduce the variance and has almost no effects on such bias (Mcdonald et al., 2009). Resolving the issue necessarily requires us to first “correct” the bias at the level of each local source node before transmitting it to the target node for information aggregation.

Various approaches have been proposed to achieve such a bias correction (Javanmard and Montanari, 2014; van de Geer et al., 2014; Ning and Liu, 2017), many of which can be expressed in the form

(13) $${\tilde{\beta }}^{\left(k\right)}={\overset{ˆ}{\beta }}_{L}^{\left(k\right)}-{\overset{ˆ}{𝚯}}^{\left(k\right)}\nabla {ℒ}^{\left(k\right)}\left({\overset{ˆ}{\beta }}_{L}^{\left(k\right)}\right),$$

where ${\overset{ˆ}{𝚯}}^{\left(k\right)}$ will be specified later. To understand how such an estimator can achieve a smaller bias than ${\overset{ˆ}{\beta }}_{L}^{\left(k\right)}$, we may rewrite (13) by subtracting $\beta_{*}^{\left(k\right)}$ from both sides and applying the mean value theorem to obtain

(14) $${\tilde{\beta }}^{\left(k\right)}-\beta_{*}^{\left(k\right)}=-{\overset{ˆ}{𝚯}}^{\left(k\right)}\nabla {ℒ}^{\left(k\right)}\left(\beta_{*}^{\left(k\right)}\right)+\left(I-{\overset{ˆ}{𝚯}}^{\left(k\right)}\nabla^{2}{ℒ}^{\left(k\right)}\left({\overset{ˆ}{\beta }}_{Int}^{\left(k\right)}\right)\right)\left({\overset{ˆ}{\beta }}_{L}^{\left(k\right)}-\beta_{*}^{\left(k\right)}\right).$$

where ${\overset{ˆ}{\beta }}_{\text{Int}}^{\left(k\right)}$ is a vector intermediating ${\overset{ˆ}{\beta }}_{L}^{\left(k\right)}$ and $\beta_{*}^{\left(k\right)}$. On the right-hand side of (14), the first term, ${\overset{ˆ}{𝚯}}^{\left(k\right)}\nabla {ℒ}^{\left(k\right)}\left(\beta_{*}^{\left(k\right)}\right)$, is expected to exhibit a similar concentration property as $\nabla {ℒ}^{\left(k\right)}\left(\beta_{*}^{\left(k\right)}\right)$ thus contributes to the variance of ${\tilde{\beta }}^{\left(k\right)}$; the second term is a product of the error of ${\overset{ˆ}{\beta }}_{L}^{\left(k\right)}$ and $I-{\overset{ˆ}{𝚯}}^{\left(k\right)}\nabla^{2}{ℒ}^{\left(k\right)}\left({\overset{ˆ}{\beta }}_{\text{Int}}^{\left(k\right)}\right)$, which is affected by the bias of ${\overset{ˆ}{\beta }}_{L}^{\left(k\right)}$. Therefore, to minimize the effect of bias in the subsequent knowledge transfer step, one should choose a ${\overset{ˆ}{𝚯}}^{\left(k\right)}$ that is a good approximation of ${\left[\nabla^{2}{ℒ}^{\left(k\right)}\left({\overset{ˆ}{\beta }}_{\text{Int}}^{\left(k\right)}\right)\right]}^{-1}$. With a careful choice of $\tilde{\beta }$, the *D-TransMission* framework could greatly reduce communication overhead while maintaining minimal performance loss compared to its centralized counterpart. In this paper, we follow the work of van de Geer et al. (2014) to construct ${\overset{ˆ}{𝚯}}^{\left(k\right)}$, with details specified in Appendix A.4. Under mild conditions, it can be shown that the chosen ${\overset{ˆ}{𝚯}}^{\left(k\right)}$ satisfies ${\text{max}}_{j\in p}\|e_{j}-{\overset{ˆ}{𝚯}}_{j}^{\left(k\right)}\nabla^{2}{ℒ}^{\left(k\right)}\left({\overset{ˆ}{\beta }}_{\text{Int}}^{\left(k\right)}{\|}_{\infty }\leq \lambda_{j}\right.$, with ${\overset{ˆ}{𝚯}}_{j}^{\left(k\right)}$ being the jth row of ${\overset{ˆ}{𝚯}}^{\left(k\right)}$. In this way, the bias term in (14) can be controlled by $\lambda_{j}$, achieving the goal of bias reduction.

**Remark 5** We note that while constructing the estimator in (13) typically involves solving p different regularized M-estimation problems, with computational complexity $O\left(p^{2}\right)$ *per node, they can be performed locally and offline. These pre-computed estimators can then be stored as pre-trained models and transmitted to the central server only when a target task arises. Moreover, the computation involving source data is decoupled from the transfer step involving the target, which automatically avoids redoing all the computations whenever the target task changes. In fact, we found that in practice* ${\tilde{\beta }}^{\left(k\right)}$ *can also be chosen to be asymptotically unbiased estimators, such as the SCAD estimator, which achieves similar performance but is much more computationally efficient. In this paper, we stick to the proposed approach as it is better suited for establishing a non-asymptotic statistical convergence guarantee. We would like to also note that the primary objective of D-TransMission is to achieve a comparable statistical rate as the centralized estimator under the communication constraint, rather than to distribute the total computational workload across nodes. Designing computationally lighter alternatives with comparable theoretical guarantees is an important direction for future research*.

### 4.2 Theoretical Guarantee

We next demonstrate that, given a sufficiently large source sample size, the *D-TransMission* estimator ${\overset{ˆ}{\beta }}_{D}^{\left(0\right)}$ can achieve the same statistical convergence rate as the *TransMission* estimator ${\overset{ˆ}{\beta }}^{\left(0\right)}$ with just one-shot communication based on pre-estimated source parameters.

We first introduce a set of regularity assumptions listed in Appendix A.4, where conditions (D1), (D3), and (D4) are standard in high-dimensional sparse regression (Lee et al., 2017). Condition (D2) assumes row-wise sparsity on $E{\left[\left(\nabla^{2}{ℒ}^{\left(k\right)}\left(\beta_{*}^{\left(k\right)}\right)\right)\right]}^{-1}$, which is also commonly imposed in related literature (van de Geer et al., 2014; Zhang and Zhang, 2014). With these additional regularity conditions, we now establish the statistical convergence rate of the *D-TransMission* estimator.

**Theorem 2** *Under Assumption 1, 2 and 3 in* Appendix A.4*, assume* $n_{T}\gg K^{2}s\;\text{log}\;p$ *and* $n_{S}\gg K^{2}s^{2}\;\text{log}\;p$. *If we construct* ${\left\{{\tilde{\beta }}^{\left(k\right)}\right\}}_{k\in [K]}$ *through* (13) *and (A.5) with parameters* ${\tilde{\lambda }}_{k}=\mu_{jk}≍\sqrt{\text{log}\;p/n_{S}}$, *then the solution of the D-TransMission problem* (11) *satisfies*

(15) $${\left\|{\overset{ˆ}{\beta }}_{D}^{\left(0\right)}-\beta_{*}^{\left(0\right)}\right\|}_{2}^{2}≲\frac{s\;\text{log}\;p}{N}+\left(\sqrt{\frac{\text{log}\;p}{n_{T}}}h\right)\wedge \frac{s\;\text{log}\;p}{n_{T}},$$

*with probability at least* $1-c_{1}\;\text{exp}\left(-c_{2}\;\text{log}\;p\right)$.

Theorem 2 demonstrates with a sufficiently large $n_{S},{\overset{ˆ}{\beta }}_{D}^{\left(0\right)}$ achieves a statistical rate of the same order as the centralized counterpart established in Corollary 1, while requiring only a single round of communication. Notice that the theorem imposes a more restrictive growth condition on the source sample size $n_{S}$ with the number of source tasks K, a requirement common to divide-and-conquer type methods. Using arguments similar to Section 3.4, one can further establish the minimax optimality of the *D-TransMission* estimator under similar conditions.

## 5 Simulation and Real Data Analysis

### 5.1 Simulation with Synthetic Data

To showcase the effectiveness of *TransMission* method and support our theoretical results, we conduct simulations under different forms of distribution shifts. We compare the empirical performance of *TransMission* and *D-TransMission* against several established baselines. Specifically, we include *Single-Lasso*, which applies $\ell_{1}$-regularized regression solely on the target task, and *Agg-Lasso*, which pools all source and target samples and applies $\ell_{1}$-regularized regression on the combined dataset. Additionally, we compare with *TransGLM* (Tian and Feng, 2023), which performs an additional de-bias step on the pooling estimator, and *TransHDGLM* (Li et al., 2024), an iterative algorithm that alternates between updating the target parameter and estimating the source-target contrasts.

We follow a similar experimental setup as in Li et al. (2022, 2024); He et al. (2024) by considering a high-dimensional regression problem with p=500 and sparsity level s=10. The true target parameter is set as ${\left(\beta_{*}^{\left(0\right)}\right)}_{j}=0.3\cdot 1_{\left\{1\leq j\leq s\right\}}$. We then generate $n_{T}=150$ independent target samples ${\left\{\left(X_{i}^{\left(0\right)},y_{i}^{\left(0\right)}\right)\right\}}_{i\in \left[n_{T}\right]}$ with

$$X_{i}^{\left(0\right)}\sim \text{N}(0,I),y_{i}^{\left(0\right)}\sim \left\{\begin{array}{l} \text{N}\left({\left(X_{i}^{\left(0\right)}\right)}^{⊤}\beta_{*}^{\left(0\right)},1\right)\;\text{for linear regression},\\ \text{Ber}\left({\left(1+\text{exp}\left(-{\left(X_{i}^{\left(0\right)}\right)}^{⊤}\beta_{*}^{\left(0\right)}\right)\right)}^{-1}\right)\;\text{for logistic regression}, \end{array}\right.$$

where N(μ,𝚺) denotes a multivariate normal distribution with mean vector μ and covariance matrix 𝚺,Ber(p) represents a Bernoulli distribution with success probability p and I stands for the identity matrix. For the source sample, we generate the true source parameters $\beta_{*}^{\left(k\right)}=\beta_{*}^{\left(0\right)}+\delta^{\left(k\right)}$ with $\delta_{j}^{\left(k\right)}\sim N\left(0,(h/50{)}^{2}\right)$ for 1≤j≤50 and $\delta_{j}^{\left(k\right)}=0$ otherwise. Unless otherwise specified, we choose the source sample size $n_{S}=200$. When generating the source samples ${\left\{\left(X_{i}^{\left(k\right)},y_{i}^{\left(k\right)}\right)\right\}}_{i\in \left[n_{S}\right]}$, we simulate two types of distribution shifts-covariate shift and parametric model shift:

#### Covariate Shift.

To assess robustness against covariate shifts, we generate independent covariates $X_{i}^{\left(k\right)}$ under two settings.

- *Homogeneous design*. Each $X_{i}^{\left(k\right)}\sim \text{N}(0,I)$.
- *Heterogeneous design*. Each $X_{i}^{\left(k\right)}\sim \text{N}\left(0,{𝚺}^{\left(k\right)}\right)$ with ${𝚺}^{\left(k\right)}={\left(A^{\left(k\right)}\right)}^{⊤}\left(A^{\left(k\right)}\right)+I$. Here $A^{\left(k\right)}$ is a random matrix with each entry equals ρ with probability ρ and equals 0 with probability 1-ρ. A larger ρ implies a more severe covariate shift.

#### Conditional Shift.

To investigate the robustness against parametric model shifts, we generate independent response $y_{i}^{\left(k\right)}$ under two settings.

- *Homogeneous design*. Each $y_{i}^{\left(k\right)}$ is generated with $\beta_{*}^{\left(k\right)}$ and $X_{i}^{\left(k\right)}$ from the same parametric model as the target task.
- *Heterogeneous design*. For logistic regression, we set $p_{ki}$ to follow a Beta distribution with the same mean as the target task but varying variance across tasks. The binary response $y_{i}^{\left(k\right)}$ is then drawn from $\text{Ber}\left(p_{ki}\right)$, introducing task-specific overdispersion (See Appendix A.5 for details).

For linear regression, we evaluate performance using prediction error on a test target dataset, while for logistic regression classification accuracy is used. Table 2 and Table 3 compare the performance of different methods on the linear and logistic regression tasks, respectively. The results lead to the following observations.

In Table 2(a), when both h and ρ are small, indicating a weak parameter contrast and covariate shift (a setting favorable to *Agg-Lasso* and *TransGLM*), *TransMission* achieves comparable prediction error to other methods. As the covariate shift level increases, i.e., under more severe distribution shifts, the prediction error of other methods increases rapidly, whereas *TransMission* exhibits only a slight degradation in performance. This effect is even more pronounced as h grows, as both *Agg-Lasso* and *TransGLM* eventually underperform the single-task Lasso baseline, while *TransMission* consistently outperforms all other methods. A similar trend is observed in the logistic regression setting (Table 3). Under homogeneous conditions, *TransMission* performs on par with existing methods, yet it demonstrates a clear advantage when covariate and parametric model shifts are present. These findings align with the theoretical results in Section 3, confirming that *TransMission* maintains comparable performance in homogeneous settings while achieving superior robustness under distribution shifts.

Table 2(b) evaluates *D-TransMission*, the communication-efficient variant of *TransMission*, in comparison to its centralized counterpart *TransMission*. As the source sample size $n_{S}$ increases, the performance of *D-TransMission* progressively approaches that of the centralized *TransMission*. This aligns with the theoretical result that with sufficiently large source data, the distributed method can achieve comparable accuracy using only a single round of communication with source parameter estimators.

We have included several additional experiments to further examine the robustness and computational behavior of the proposed method. Specifically, we evaluate performance under heavy-tailed error distributions, where the noise follows a Student-t distribution with varying degrees of freedom. We also evaluate performance under varying feature dimensions p, to assess scalability in high-dimensional regimes. The corresponding results and detailed discussions are provided in Appendix C.

### 5.2 Real-Data Studies

We evaluate the proposed TransMission method on two real-data applications: a high-dimensional gene expression prediction task under a linear regression model and a handwritten-digit classification task under a logistic regression model. Together, these studies assess the method in both biological and image-based settings with heterogeneous source-target relationships.

#### Gene expression analysis.

We study a real-world high-dimensional gene expression prediction problem based on the GTEx Portal dataset. Following established practice in the genomics literature (e.g., Li et al., 2022), we investigate how genes associated with the central nervous system (CNS) influence the expression of a protein-coding gene across multiple tissues. After preprocessing and filtering, we obtain approximately p≈1,600 covariates. We treat the brain tissues as source tasks and each non-brain tissue as a target task, resulting in heterogeneous sample sizes across targets. We evaluate prediction performance using five-fold cross-validation on each target tissue, with mean squared error (MSE) computed on held-out samples and averaged across folds.

As reported in Table 4, *TransMission* achieves the best average MSE among all competing methods, improving upon the second-best method by a substantial margin. Moreover, *TransMission* attains the best performance in 32 out of 36 target tissues and ranks second in the remaining 4 tissues, demonstrating consistently strong performance across a wide range of tissue-specific tasks. These results confirm the effectiveness of *TransMission* in high-dimensional biological settings.

#### Handwritten-digit classification.

We next evaluate the proposed TransMission method on a handwritten-digit classification task using the MNIST dataset and a corrupted variant from MNIST-C, where images are affected by impulse noise (Mu and Gilmer, 2019). This type of corruption randomly perturbs pixel intensities, introducing structured distribution shifts that test the robustness of different transfer learning methods under a logistic regression model.

To construct the multi-source binary classification task, we define four different source datasets (K=4) and one target dataset. The target dataset consists of clean images from MNIST, while the source datasets are drawn from impulse noise-corrupted MNIST-C images. Each source dataset consists of 100 training samples ($n_{S}=100$), while the target dataset contains 50 training samples ($n_{T}=50$). All images are represented as flattened pixel vectors, resulting in a 784-dimensional feature space (p=784). For each digit, we create a binary classification problem where that digit serves as the positive class, and the negative class is sampled from other digits. To introduce the parameter contrast $\delta^{\left(k\right)}=\beta^{\left(k\right)}-\beta^{\left(0\right)}$ across sources, each source dataset is assigned a different negative-class digit. For example, if the positive class is digit “3”, one source dataset may use digit “5” as the negative class, while another source may use digit “7”. In addition, the impulse noise applied to source datasets introduces further distribution shifts, allowing us to evaluate how each method captures transferable parameter information under heterogeneous conditions.

**Figure 1:**
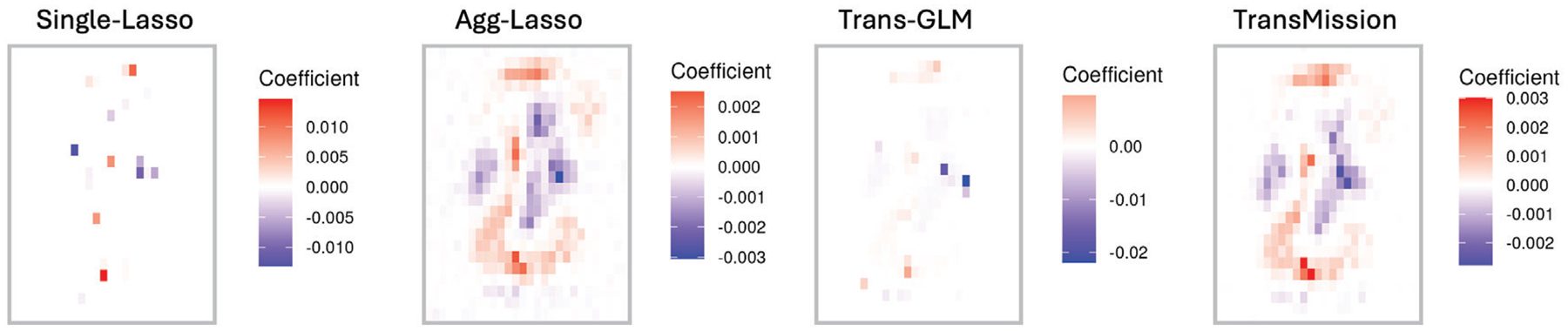
Heatmaps of learned coefficients for classifying digit 8 (positive class) vs. digit 9 (negative class) across different methods. Red shows pixels that strongly contribute to predicting an 8, while blue shows pixels that discourage classification as an 8.

We evaluate performance using classification accuracy on an independent set of 2000 target samples. The results, averaged over 100 independent replications, are reported in Table 5, which presents test classification accuracy for the 10 binary classification tasks. TransMission achieves the highest accuracy in 8 out of 10 cases, demonstrating its effectiveness in leveraging transferable information while mitigating distribution shifts. Notably, its improvement is most noticeable in classifying digit 8. To further investigate this, we analyze the heatmaps of learned parameters for each method in Figure 1, where red indicates positive pixel importance and blue represents negative pixel importance. *Single-Lasso*, constrained by the limited target sample size, focuses on a sparse set of pixels. Agg-Lasso captures more informative patterns but fails to adapt to distribution shifts, as shown by the background blue pixels indicating overfitting to injected noise. *Trans-GLM* primarily emphasizes pixels from the target dataset, neglecting transferable patterns from sources. In contrast, *TransMission* effectively extracts useful features from source data while filtering out irrelevant background noise, resulting in a more robust and generalizable model.

## 6 Conclusion and Future Work

We propose *TransMission*, a parameter-transfer framework for high-dimensional M-estimation. The joint regularized training process ensures effective knowledge transfer while remaining resilient to distribution shifts. A novel target-data-oriented constraint is introduced to fully exploit the target data to identify the target parameter, as well as prevent negative transfer issues. Our theoretical analysis establishes minimax-optimal statistical rates in the absence of shifts and demonstrates robustness when shifts are present. In addition, we introduce D-*TransMission*, a communication-efficient variant using only pre-estimated source parameters. Extensive experiments validate our theoretical findings.

A number of research questions can be further studied from the framework and methods developed here. First, our analysis is built upon an M-estimation setting, an important future direction is to extend the proposed framework to more general nonlinear structures, such as nonparametric models. Second, while the current theoretical results rely on standard regularity assumptions such as Restricted Strong Convexity/Smoothness (cf. Assumption 1) and the sparsity of the inverse Fisher information matrix (cf. Assumption 3), it is interesting to explore ways to relax these conditions.

## Supplementary Material

Supplement

## Figures and Tables

**Table 1:** Comparison of different parameter transfer methods. LM and GLM denote linear and generalized linear models, respectively. Source detection indicates whether an additional transferable source detection step is performed. $C_{\Sigma }$, defined in (10), quantifies the level of distribution shift.

Method	Setting	Source Detection	Statistical Rate
TransLasso (Li et al., 2022)	LM	No	$\frac{s\;\text{log}\;p}{N}+\left(C_{\Sigma }h\sqrt{\frac{\text{log}\;p}{n_{T}}}\right)\wedge \left(C_{\Sigma }^{2}h^{2}\right)$
		Yes	$\frac{s\;\text{log}\;p}{N}+\left(C_{\Sigma }h\sqrt{\frac{\text{log}\;p}{n_{T}}}\right)\wedge \left(C_{\Sigma }^{2}h^{2}\right)\wedge \left(\frac{s\;\text{log}\;p}{n_{T}}\right)+\frac{\text{log}\;K}{n_{T}}$
TransGLM (Tian and Feng, 2023)	GLM	-	$\frac{s\;\text{log}\;p}{N}+\left(C_{\Sigma }h\sqrt{\frac{\text{log}\;p}{n_{T}}}\right)\wedge \left(C_{\Sigma }^{2}h^{2}\right)$
TransMission (Corollary 2)	M-estimator	-	$\frac{s\;\text{log}\;p}{N}+\left(h\sqrt{\frac{\text{log}\;p}{n_{T}}}\right)\wedge \left(C_{\Sigma }^{2}h^{2}\right)\wedge \left(\frac{s\;\text{log}\;p}{n_{T}}\right)$

**Table 2:** Prediction error comparison in the linear regression case. (a) comparison of different methods across varying levels of covariate shift (ρ) and parameter contrast (h). The best performance for each setting is highlighted in bold. (b) comparison of *TransMission* and *D-TransMission* errors across different values of ρ and source sample sizes $\left(n_{S}\right)$ with h=20.

h	ρ	Agg-Lasso	Single-Lasso	Trans-GLM	TransHDGLM	TransMission	ρ	$n_{S}$	TransMission	D-TransMission
5	0.1	0.132	0.894	0.171	**0.123**	0.134	0.0	50	0.698	1.054
5	0.2	0.141	0.891	0.162	**0.127**	0.136	0.0	100	0.537	0.937
5	0.4	0.172	0.896	0.194	**0.158**	0.160	0.0	150	0.357	0.617
10	0.1	0.320	0.895	0.329	0.239	**0.197**	0.0	200	0.297	0.438
10	0.2	0.372	0.894	0.308	0.281	**0.206**	0.0	250	0.258	0.310
10	0.4	0.433	0.893	0.374	0.335	**0.254**	0.0	300	0.224	0.260
15	0.1	0.651	0.894	0.534	0.440	**0.251**	0.3	50	0.665	1.021
15	0.2	0.782	0.894	0.540	0.497	**0.274**	0.3	100	0.515	0.845
15	0.4	0.884	0.885	0.615	0.586	**0.324**	0.3	150	0.414	0.610
20	0.1	1.095	0.894	0.874	0.663	**0.301**	0.3	200	0.348	0.440
20	0.2	1.339	0.887	0.882	0.704	**0.326**	0.3	250	0.326	0.355
20	0.4	1.439	0.886	0.927	0.805	**0.404**	0.3	300	0.300	0.303
(a)							(b)			

**Table 3:** Classification accuracy comparison in the logistic regression case. Comparison of different methods across different heterogeneity levels (h) and shift types (no shifts, covariate shifts, both covariate and parametric model shifts). The best performance for each setting is highlighted in bold.

h	Shift Type	Agg-Lasso	Single-Lasso	Trans-GLM	TransHDGLM	TransMission
5	none	**0.662**	0.534	0.644	0.655	0.647
5	covariate	0.675	0.537	0.657	0.671	**0.676**
5	both	0.664	0.524	0.650	0.653	**0.664**
15	none	**0.622**	0.540	0.606	0.618	0.621
15	covariate	0.603	0.538	0.591	0.575	**0.621**
15	both	0.610	0.524	0.591	0.560	**0.627**
25	none	0.572	0.533	0.561	0.560	**0.586**
25	covariate	0.559	0.533	0.559	0.533	**0.579**
25	both	0.564	0.519	0.554	0.524	**0.588**

**Table 4:** Performance comparison for different target tissues. The best performance for each setting is highlighted in bold. The second best performance is underlined.

Target Tissue	Agg-Lasso	Single-Lasso	Trans-GLM	TransHDGLM	TransMission
adipose subcutaneous	158.82	181.86	105.12	185.44	**70.81**
adipose visceral omentum	138.60	173.07	88.30	178.34	**76.24**
adrenal gland	54.45	68.79	57.60	57.04	**46.29**
artery aorta	19.85	24.95	**17.33**	25.20	18.78
artery coronary	82.81	74.45	**66.63**	72.52	70.87
artery tibial	20.96	32.78	18.71	31.95	**11.16**
bladder	63.28	68.29	58.47	70.13	**46.61**
breast mammary tissue	194.30	249.33	212.79	250.59	**67.86**
cells cultured fibroblasts	17.80	14.67	20.23	16.33	**4.02**
cells ebv-transformed lymphocytes	7.52	11.96	12.10	12.56	**4.33**
colon sigmoid	107.23	157.50	82.94	163.35	**40.24**
colon transverse	79.76	98.50	83.36	100.55	**29.66**
esophagus gastroesophageal junction	98.29	131.02	72.41	135.69	**53.46**
esophagus mucosa	29.51	29.53	27.56	29.66	**6.34**
esophagus muscularis	197.70	211.08	177.15	208.13	**78.73**
heart atrial appendage	11.77	11.40	11.22	11.05	**9.49**
heart left ventricle	10.85	14.99	11.28	12.04	**8.21**
kidney cortex	39.70	69.01	38.85	54.62	**41.63**
liver	22.08	21.90	22.28	22.78	**1.96**
lung	278.73	281.14	**156.71**	285.86	247.45
minor salivary gland	22.73	29.19	27.31	32.99	**15.90**
muscle skeletal	3.05	8.73	2.97	8.80	**1.58**
nerve tibial	68.51	76.58	34.13	76.99	**29.91**
ovary	72.68	76.15	73.69	77.18	**64.16**
pancreas	9.90	9.95	10.07	9.99	**1.74**
pituitary	121.04	179.89	110.82	206.03	**62.01**
prostate	75.00	78.77	72.15	86.13	**43.24**
skin not sun exposed suprapubic	12.67	20.88	13.25	21.04	**5.86**
skin sun exposed lower leg	11.99	20.00	11.74	19.74	**6.29**
small intestine terminal ileum	63.02	88.94	63.49	85.56	**55.84**
spleen	2.78	8.42	2.21	8.27	**1.34**
stomach	36.18	53.47	37.45	55.98	**16.61**
testis	95.37	95.18	57.98	97.10	**12.39**
thyroid	53.40	64.49	53.84	66.30	**36.70**
uterus	205.43	296.47	**128.50**	293.40	138.60
vagina	88.14	127.75	89.96	139.63	**82.97**
Average	71.55	87.81	59.18	89.14	**41.92**

**Table 5:** Classification accuracy (%) for different digits using various methods. The best performance for each digit is highlighted in bold.

Method	Digit									
	0	1	2	3	4	5	6	7	8	9
Single-lasso	97.72	95.89	91.09	92.63	89.82	86.46	96.97	89.82	86.93	86.80
Agg-Lasso	97.88	97.20	93.99	90.84	82.47	94.65	98.49	88.50	92.72	86.14
Trans-GLM	97.79	**97.57**	94.45	92.76	**90.08**	91.61	98.47	89.88	90.28	89.75
TransHDGLM	96.37	94.95	90.85	87.39	86.06	84.28	93.96	84.00	85.01	85.40
TransMission	**97.94**	97.38	**95.24**	**93.35**	87.87	**94.85**	**98.73**	**90.51**	**94.81**	**90.12**
