# Supplementary material for: High-dimensional Parameter Transfer With Fused-Regularizer
Source: J Mach Learn Res. Author manuscript; Available in PMC 2026 Aug 12. (PMC13459905)
Supplement: Supplementary file 1 [file NIHMS2177573-supplement-Supplement.pdf]

Appendix A. Additional Methodology Details

A.1 Notations

Table A.1: Summary of notation used throughout the paper.

Notation	Description
$\mathbf{x}, \mathbf{x}_i$	Vector in $\mathbb{R}^p$ and its ith entry.
$\boldsymbol{A}, \boldsymbol{A}_i, \boldsymbol{A}_{ij}$	Matrix in $\in \mathbb{R}^{m\times n}$, its ith row, and its (i,j)th entry.
$\langle\cdot,\cdot\rangle$	Euclidean inner product.
$\\|\mathbf{x}\\|_q$	ℓ_q-norm: $\\|\mathbf{x}\\|_q = (\sum_{i=1}^p \lvert\mathbf{x}_i\rvert^q)^{1/q}$, for $q > 0$.
$\\|\mathbf{x}\\|_0$	ℓ_0-norm: $\\|\mathbf{x}\\|_0 = \#\{j : \mathbf{x}_j \neq 0\}$.
$\\|\mathbf{x}\\|_\infty$	ℓ_∞-norm: $\\|\mathbf{x}\\|_\infty = \max_{1\le j\le p} \lvert\mathbf{x}_j\rvert$.
$a \vee b$, $a \wedge b$	$\max\{a, b\}$ and $\min\{a, b\}$, respectively.
$[K]$	Index set $\{1, \dots, K\}$.
$c, c_0, c_1, \dots$	Generic positive constants.
$a_n = O(b_n)$, $a_n \lesssim b_n$	$\lvert a_n/b_n\rvert \le c$ for some constant c (for large n).
$a_n \asymp b_n$	$a_n = O(b_n)$ and $b_n = O(a_n)$.
$a_n = o(b_n)$, $b_n \gg a_n$	$a_n/(b_n) \to 0$ (equivalently, b_n dominates a_n).
p	Feature dimension.
n_T, n_S	Target and per-source sample sizes.
K	Number of source tasks.
$z_i^{(k)}$	A single data point from the k-th task (e.g., $z_i^{(k)} = (\mathbf{X}_i^{(k)}, \mathbf{y}_i^{(k)})$).
$\mathcal{S}^{(0)}$	Target sample, i.i.d. from the target distribution: $\mathcal{S}^{(0)} := \{z_i^{(0)}\}_{i\in[n_T]}$.
$\mathcal{S}^{(k)}$	k-th source sample, i.i.d. from the source distribution: $\mathcal{S}^{(k)} := \{z_i^{(k)}\}_{i\in[n_S]}$
$\ell^{(0)}(z^{(0)}; \boldsymbol{\beta})$	Target loss evaluated at parameter $\boldsymbol{\beta} \in \mathbb{R}^p$.
$\ell^{(k)}(z^{(k)}; \boldsymbol{\beta})$	k-th source loss evaluated at parameter $\boldsymbol{\beta} \in \mathbb{R}^p$.
$\mathcal{L}^{(0)}(\boldsymbol{\beta}^{(0)})$	Empirical loss for the target task: $\mathcal{L}^{(0)}(\boldsymbol{\beta}^{(0)}) = (2n_T)^{-1}\sum_{i=1}^{n_T} \ell^{(0)}(z_i^{(0)}; \boldsymbol{\beta}^{(0)})$.
$\mathcal{L}^{(k)}(\boldsymbol{\beta}^{(k)})$	Empirical loss for the k-th source task: $\mathcal{L}^{(k)}(\boldsymbol{\beta}^{(k)}) = (2n_S)^{-1}\sum_{i=1}^{n_S} \ell^{(k)}(z_i^{(k)}; \boldsymbol{\beta}^{(k)})$.
$\boldsymbol{\beta}_*^{(0)}$	Target parameter, $\boldsymbol{\beta}_*^{(0)} \in \arg\min_{\boldsymbol{\beta}}\ \mathbb{E}_{z^{(0)}\sim\mathbb{P}^{(0)}}[\ell^{(0)}(z^{(0)}; \boldsymbol{\beta})]$.
$\boldsymbol{\beta}_*^{(k)}$	k-th source parameter, $\boldsymbol{\beta}_*^{(k)} \in \arg\min_{\boldsymbol{\beta}}\ \mathbb{E}_{z^{(k)}\sim\mathbb{P}^{(k)}}[\ell^{(k)}(z^{(k)}; \boldsymbol{\beta})]$.
s	Sparsity level of the target parameter: $s := \\|\boldsymbol{\beta}_*^{(0)}\\|_0$
$\boldsymbol{\delta}^{(k)}$	Parameter contrast (source-specific signal) for task k: $\boldsymbol{\delta}^{(k)} := \boldsymbol{\beta}_*^{(k)} - \boldsymbol{\beta}_*^{(0)}$.
h	Upper bound on informativeness level: $\\|\boldsymbol{\delta}^{(k)}\\|_1 \le h$.
$\mathbf{X}_i^{(k)}$, $\mathbf{y}_i^{(k)}$	Covariate/response for the i-th sample in source task k.
$\boldsymbol{\Sigma}^{(k)}$	Covariance matrix of $\mathbf{X}_i^{(k)}$ in source task k.
$\boldsymbol{\epsilon}_i^{(k)}$	Noise term in linear models for task k.
$\mathbb{P}_X^{(k)}$, $\mathbb{P}_{y\mid X}^{(k)}$	Marginal covariate and conditional response distributions for task k.
$\Psi_k(\cdot)$, ϕ_k	Cumulant generating function function and dispersion parameter for task k.
$\nabla^l g(\boldsymbol{\beta})$	l-th order derivative of a scalar function $g(\cdot)$ at $\boldsymbol{\beta}$.
$\nabla_j^l g(\boldsymbol{\beta})$	Univariate l-th order partial derivative w.r.t. coordinate j.
$\boldsymbol{e}_j$	The j-th canonical basis vector, whose j-th entry is 1 and all others are 0.

A.2 Practical Implementation and Tuning Parameter Selection

This subsection is dedicated to the practical implementation of *TransMission*, with a detailed guide for tuning parameter selection. For illustration purposes, we take the generalized linear model with a known and shared dispersion parameter ϕ and a log-partition function $\Phi(\cdot)$. Extensions to settings with task-specific ϕ_k and $\Phi_k(\cdot)$ follow analogously by replacing the shared quantities with their task-specific counterparts. The optimization problem consists of two components: the objective function (6a) and the constraint (6b).

For solving objective (6a), based on the one-to-one transformation $\boldsymbol{\theta} := ((\boldsymbol{\beta}^{(1)} - \boldsymbol{\beta}^{(0)})^\top, (\boldsymbol{\beta}^{(2)} - \boldsymbol{\beta}^{(0)})^\top, \ldots, (\boldsymbol{\beta}^{(K)} - \boldsymbol{\beta}^{(0)})^\top, \boldsymbol{\beta}^{(0)})$, we can reformulate the problem into the following standard regularized regression problem:

$$\hat{\boldsymbol{\theta}} \in \underset{\boldsymbol{\theta}}{\operatorname{argmin}} \left\{ \frac{1}{N\phi} \left(-\mathbf{y}^T \mathbf{X}\boldsymbol{\theta} + \sum_{i=1}^{N} \Phi\left(\mathbf{X}_i^\top \boldsymbol{\theta} \right) \right) + \lambda_0 \sum_{k=0}^{K} a_k \|\boldsymbol{\theta}^{(k)}\|_1 \right\}, \tag{A.1}$$

where $a_0 = 1$ and $a_1 = \frac{\lambda_1}{\lambda_0}$ for $k \in [K]$, and $\mathbf{y}$ and $\mathbf{X}$ are defined as

$$\mathbf{y} := \begin{pmatrix} \mathbf{y}^{(1)} \\ \mathbf{y}^{(2)} \\ \vdots \\ \mathbf{y}^{(K)} \\ \mathbf{y}^{(0)} \end{pmatrix} \quad \mathbf{X} := \begin{pmatrix} \mathbf{X}^{(1)} & 0 & \cdots & 0 & \mathbf{X}^{(1)} \\ 0 & \mathbf{X}^{(2)} & \cdots & 0 & \mathbf{X}^{(2)} \\ \vdots & \vdots & \vdots & \vdots & \vdots \\ 0 & 0 & \cdots & \mathbf{X}^{(K)} & \mathbf{X}^{(K)} \\ 0 & 0 & \cdots & 0 & \mathbf{X}^{(0)} \end{pmatrix}.$$

Such an objective falls into the class of ℓ_1-penalized regression problems and can therefore be efficiently solved via the standard `glmnet` library (Friedman et al., 2010). In this formulation, the tuning parameter λ_0 is selected through a three-fold cross-validation of the target training dataset, while a_k is fixed according to the theoretical ratio established in Corollary 1 with $h = 0$, namely $a_k = \frac{n_S}{\sqrt{N n_T}}$, where n_S and n_T denote the sample sizes of the source and target data, respectively. This theoretically informed procedure has shown consistently strong performance across a wide range of settings.

We next discuss the practical handling of constraint (6b). Rather than enforcing the constraint during optimization, which would make the problem substantially harder to solve, we adopt a verification-based approach.

We consider a geometric grid of λ_T values. For each λ_T, we compute a single-task reference estimator

$$\hat{\boldsymbol{\beta}}_T^{(0)}(\lambda_T) \in \underset{\boldsymbol{\beta} \in \mathbb{R}^p}{\operatorname{argmin}} \left\{ \mathcal{L}^{(0)}(\boldsymbol{\beta}) + \lambda_T \|\boldsymbol{\beta}\|_1 \right\}, \tag{A.2}$$

which yields the reference ℓ_1 norm $\|\hat{\boldsymbol{\beta}}_T^{(0)}\|_1$ and sparsity estimate $\hat{s} = \|\hat{\boldsymbol{\beta}}_T^{(0)}\|_0$. Following Theorem 1, which prescribes $\kappa_T \propto s\lambda_T$, we set $\kappa_T = \hat{s}\lambda_T$ as a practical plug-in, replacing the unknown true sparsity s with the estimated $\hat{s}$ and absorbing the proportionality constant. Together, λ_T and κ_T define the constraint set $\mathcal{C}_T(\lambda_T)$.

For each λ_T, we solve the unconstrained weighted-Lasso problem (A.1) over a grid of λ_0 values via three-fold cross-validation on the target data, obtaining candidate estimators

$\{\hat{\boldsymbol{\beta}}(\lambda_0)\}$ together with their validation losses. For each pair (λ_0, λ_T), we verify whether $\hat{\boldsymbol{\beta}}(\lambda_0) \in \mathcal{C}_T(\lambda_T)$, i.e., whether

$$\|\nabla \mathcal{L}_0(\hat{\boldsymbol{\beta}}(\lambda_0))\|_\infty \le \lambda_T \quad \text{and} \quad \|\hat{\boldsymbol{\beta}}(\lambda_0)\|_1 \le \|\hat{\boldsymbol{\beta}}_T^{(0)}\|_1 + \hat{s}\lambda_T.$$

Among all feasible pairs, we select the one with the lowest target validation loss. If no feasible pair exists, we fall back to the single-task estimator.

In summary, *TransMission* involves two tuning parameters, λ_0 and λ_T, jointly selected by TransMission validation performance subject to the solution lying in $\mathcal{C}_T(\lambda_T)$. This procedure consistently delivers strong performance across a wide range of settings.

A.3 Choice of Tuning Parameters in Corollary 2

Recall that we define a pooling estimator $\hat{\boldsymbol{\beta}}_{\text{Pooling}}$ in (4) and its population counterpart as $\boldsymbol{\beta}_{\text{Pooling}}$ in (9). We also define a factor C_Σ in (10) that measures the distribution shifts across sources. To establish a faster convergence rate compared with (8), we adopt the following choice of tuning parameters:

$$\lambda_0 \asymp \left[\left(\frac{h^2 \log p}{s^2 n_T}\right)^{1/4} + \left(\frac{\log p}{N}\right)^{1/2}\right], \quad \lambda_1 \asymp \frac{n_S}{N}\left(\frac{\log p}{n_T}\right)^{1/2}, \quad \text{and} \quad \lambda_T \asymp \left(\frac{\log p}{n_T}\right)^{1/2}, \tag{A.3}$$

if $h \le (C_\Sigma^{-2}\sqrt{\log p/n_T}) \wedge (C_\Sigma^{-1}\sqrt{s\log p/n_T})$ and

$$\lambda_0 \asymp \sqrt{\frac{\log p}{N}}, \lambda_1 \gtrsim \left\|\frac{n_S}{N}\nabla \mathcal{L}^{(k)}(\hat{\boldsymbol{\beta}}_{\text{Pooling}})\right\|_\infty, \tag{A.4}$$

otherwise. To understand the rationale behind this parameter selection, note that under (A.4), λ_1 is chosen sufficiently large to shrink all $\hat{\boldsymbol{\beta}}^{(k)}$ toward $\hat{\boldsymbol{\beta}}^{(0)}$. As a result, the TransMission estimator $\hat{\boldsymbol{\beta}}^{(0)}$ effectively reduces to the pooled estimator $\hat{\boldsymbol{\beta}}_{\text{Pooling}}$, which is optimal in homogeneous settings where source and target distributions are nearly identical. In other cases, the parameters follow the original choices in Corollary 1, as specified in (A.3).

As the condition (A.4) depends on the estimator $\hat{\boldsymbol{\beta}}_{\text{Pooling}}$, in the following, we further establish a sufficient condition for (A.4) to hold. The result requires an additional ℓ_∞ curvature assumption, which is similar to the RSM condition but is measured in terms of ℓ_∞ norm. Such an assumption also holds for common regression models (Wainwright, 2019).

Lemma 1 *Under the assumption of Corollary 2, if we in addition assume that for any $k \in [K]$, $\boldsymbol{\beta}, \boldsymbol{\Delta} \in \mathbb{R}^p$, there exists constants $\eta_k, \zeta_k \ge 0$ such that*

$$\|\nabla \mathcal{L}^{(k)}(\boldsymbol{\beta} + \boldsymbol{\Delta}) - \nabla \mathcal{L}^{(k)}(\boldsymbol{\beta})\|_\infty \le \eta_k \|\boldsymbol{\Delta}\|_\infty + \zeta_k \sqrt{\frac{\log p}{n_S}}\|\boldsymbol{\Delta}\|_1,$$

with probability larger than $1 - c_1\exp(-c_2\log p)$. Then a sufficient condition for $\lambda_1 \gtrsim \|\frac{n_S}{N}\nabla \mathcal{L}^{(k)}(\hat{\boldsymbol{\beta}}_{Pooling})\|_\infty$ is

$$\lambda_1 \gtrsim \frac{n_S}{N}\left[K_P + \sqrt{\frac{\log p}{N}} + \sqrt{\frac{\log p}{n_S}}\left(s\sqrt{\frac{\log p}{N}} + C_\Sigma h\right)\right],$$

where $K_P = \max_{k\in[K]}\|\boldsymbol{\beta}_{Pooling} - \boldsymbol{\beta}_*^{(k)}\|_\infty$ is the maximum elementwise difference between $\boldsymbol{\beta}_{Pooling}$ and the true source parameter $\boldsymbol{\beta}_*^{(k)}$.

A.4 Additional Details On D-TransMission Method

In this paper, we construct $\hat{\mathbf{\Theta}}^{(k)}$ to be the de-sparsified lasso proposed in van de Geer et al. (2014). Following their framework, we limit our discussion to a regression framework with covariables $\mathbf{X}_i^{(k)} \in \mathcal{X}^{(k)} \subseteq \mathbb{R}^p$ and univariate responses $\mathbf{y}_i^{(k)} \in \mathcal{Y}^{(k)} \subseteq \mathbb{R}$ for $i = 1, \ldots, n_S$, and the source task loss function has the form

$$\ell^{(k)}(\boldsymbol{\beta}; z_i^{(k)}) = \rho^{(k)}((\mathbf{X}_i^{(k)})^\top \boldsymbol{\beta}, \mathbf{y}_i^{(k)}).$$

We assume ρ is locally Lipschitz with well-defined second-order derivative $\ddot{\rho}^{(k)}(a,b) = \frac{d^2}{da^2}\rho(a,b)$. Many commonly used regression models, such as the generalized linear regression discussed in Example 2, fit within this framework. Within this setup, the problem could be formulated into a linear regression problem with the weighted design matrix $\mathbf{X}_{\hat{\boldsymbol{\beta}}_L^{(k)}} := \boldsymbol{W}_{\hat{\boldsymbol{\beta}}_L^{(k)}} \mathbf{X}^{(k)}$, where $\boldsymbol{W}_{\hat{\boldsymbol{\beta}}_L^{(k)}}$ is diagonal and its diagonal entries defined as $(\boldsymbol{W}_{\hat{\boldsymbol{\beta}}_L^{(k)}})_{i,i} := \ddot{\rho}^{(k)}((\mathbf{X}_i^{(k)})^\top \hat{\boldsymbol{\beta}}_L^{(k)}, \mathbf{y}_i^{(k)})^{\frac{1}{2}}$. van de Geer et al. (2014) proposes to estimate $\hat{\mathbf{\Theta}}^{(k)}$ by constructing its j-th row as the solution to the following nodewise-regression problem:

$$\hat{\mathbf{\Theta}}_{jk}^{(k)} = -\hat{\tau}_j^{-2}\hat{\gamma}_{jk}, \hat{\mathbf{\Theta}}_{jj}^{(k)} = 1 \text{ with } \hat{\gamma}_j^{(k)} := \operatorname*{argmin}_{\boldsymbol{\gamma}\in\mathbb{R}^{p-1}} \frac{1}{2n_S}\left\|\mathbf{X}^{(k)}_{\hat{\boldsymbol{\beta}}_L^{(k)},j} - \mathbf{X}^{(k)}_{\hat{\boldsymbol{\beta}}_L^{(k)},-j}\gamma\right\|_2^2 + \mu_{jk}\|\boldsymbol{\gamma}\|_1,$$

$$\text{and } \hat{\tau}_j := \left(\frac{1}{2n_S}\left\|\mathbf{X}^{(k)}_{\hat{\boldsymbol{\beta}}_L^{(k)},j} - \mathbf{X}^{(k)}_{\hat{\boldsymbol{\beta}}_L^{(k)},-j}\hat{\gamma}_j^{(k)}\right\|_2^2 + \mu_{jk}\|\hat{\gamma}_j^{(k)}\|_1\right)^{\frac{1}{2}}. \tag{A.5}$$

with μ_{jk} being the tuning parameters for $j \in [p]$.

To establish the theoretical results, we introduce the following regularity conditions.

Assumption 3 *(D1) The pairs of random variables* $\left\{\left(\mathbf{y}_i^{(k)}, \mathbf{X}_i^{(k)}\right)\right\}_{i\in[n_S]}$ *are i.i.d. and* $\|\mathbf{X}^{(k)}\|_\infty = \max_{i,j}\left|\mathbf{X}_{i,j}^{(k)}\right| = \mathcal{O}(1)$ *and* $\left\|\mathbf{X}^{(k)}_{\boldsymbol{\beta}_*^{(k)},-j}\gamma^{(k)}_{\boldsymbol{\beta}_*^{(k)},j}\right\|_\infty = \mathcal{O}(1)$, *where*

$$\boldsymbol{\gamma}_{\boldsymbol{\beta}_*^{(k)},j} := \operatorname*{arg\,min}_{\boldsymbol{\gamma}\in\mathbb{R}^{p-1}} \mathbb{E}\left[\left\|\mathbf{X}_{\boldsymbol{\beta}_*^{(k)},j} - \mathbf{X}_{\boldsymbol{\beta}_*^{(k)},-j}\gamma\right\|_2^2\right].$$

(D2) Denote $s_j^{(k)} = \|\mathbb{E}[(\nabla^2\mathcal{L}^{(k)}(\boldsymbol{\beta}_*^{(k)}))_j^{-1}]\|_0$. *It holds that* $s_j^{(k)}\sqrt{\log p/n_S} = o(1)$.

(D3) Define $\mathbf{\Sigma}^{(k)}_{\boldsymbol{\beta}_*^{(k)}} := \frac{1}{n_S}\mathbb{E}[(\mathbf{X}^{(k)}_{\boldsymbol{\beta}_*^{(k)}})^\top \mathbf{X}^{(k)}_{\boldsymbol{\beta}_*^{(k)}}]$. *The smallest and largest eigenvalue of* $\mathbf{\Sigma}^{(k)}_{\boldsymbol{\beta}_*^{(k)}}$ *is bounded uniformly over* $k \in [K]$.

(D4) For some $\boldsymbol{\delta} > 0$ *and all* $\left\|\boldsymbol{\beta} - \boldsymbol{\beta}_*^{(k)}\right\|_1 \le \boldsymbol{\delta}$, *it holds that* $\boldsymbol{W}_{\boldsymbol{\beta}}$ *stays away from zero and that* $\|\boldsymbol{W}_{\boldsymbol{\beta}}\|_\infty = \mathcal{O}(1)$. *We further require that for all such* $\boldsymbol{\beta}$ *and all* $\mathbf{x}$ *and* $\mathbf{y}$,

$$\left|\nabla^2\rho^{(k)}(\mathbf{x}\boldsymbol{\beta}, \mathbf{y}) - \nabla^2\rho^{(k)}(\mathbf{x}\boldsymbol{\beta}_*^{(k)}, \mathbf{y})\right| \le \left|\mathbf{x}^\top\left(\boldsymbol{\beta}_*^{(k)} - \boldsymbol{\beta}\right)\right|.$$

A.5 Heterogeneous Binomial Regression: Overdispersion Setup

To introduce overdispersion in the binomial regression tasks, we model each source task using a Beta-Binomial framework. Instead of using a fixed probability parameter p_k determined

directly by the logistic function, we assume that p_k follows a Beta distribution with task-specific shape parameters, introducing additional variability across tasks. For the k-th source task, we first compute a baseline linear predictor η_{ki} using the standard logistic regression model with $\eta_{ki} = (\mathbf{X}_i^{(k)})^\top \boldsymbol{\beta}^{(k)}$. In the following for notational simplicity we drop the subscript i. Then, instead of defining the probability parameter as $p_k = (1 + e^{-\eta_k})^{-1}$, we generate p_k from a Beta distribution with parameters α_k and β_k, ensuring that the mean of p_k aligns with the logistic function while its variance varies across tasks. Specifically, we define $\alpha_k = \alpha_0$ and $\beta_k \sim U(\beta_{\min}, \beta_{\max})$. The shape parameters of the Beta distribution are then computed as:

$$\alpha_{\text{source},k} = \alpha_k \cdot \frac{e^{\eta_k}}{1 + e^{\eta_k}}, \quad \beta_{\text{source},k} = \beta_k \cdot \left(1 - \frac{e^{\eta_k}}{1 + e^{\eta_k}}\right).$$

Given the Beta distribution parameters, the probability p_k for each sample in the k-th source task is drawn as:

$$p_k \sim \text{Beta}(\alpha_{\text{source},k}, \beta_{\text{source},k}).$$

Finally, the binary response y_k is sampled from a Bernoulli distribution with success probability p_k:

$$y_k \sim \text{Bernoulli}(p_k).$$

This formulation allows the mean of p_k to remain close to the target probability, while the variance of p_k changes across tasks.

Appendix B. Proof of Theorems and Propositions

In this section, we provide proof of all the theorems and propositions. Throughout the section, we adopt the following notations to analyze the solution of the problem (6):

$$\boldsymbol{\theta}_* = \begin{pmatrix} \boldsymbol{\theta}_*^{(1)} \\ \boldsymbol{\theta}_*^{(2)} \\ \vdots \\ \boldsymbol{\theta}_*^{(K)} \\ \boldsymbol{\theta}_*^{(0)} \end{pmatrix} := \begin{pmatrix} \boldsymbol{\beta}_*^{(1)} - \boldsymbol{\beta}_*^{(0)} \\ \boldsymbol{\beta}_*^{(2)} - \boldsymbol{\beta}_*^{(0)} \\ \vdots \\ \boldsymbol{\beta}_*^{(K)} - \boldsymbol{\beta}_*^{(0)} \\ \boldsymbol{\beta}_*^{(0)} \end{pmatrix}, \tag{A.6}$$

where $\boldsymbol{\beta}_*^{(k)}$s are the true source parameters defined in (2) and $\boldsymbol{\beta}_*^{(0)}$ is the true target parameter defined in (1). Under this transformation, solving problem (6) is equivalent as solving

$$\hat{\boldsymbol{\theta}} \in \operatorname*{argmin}_{\boldsymbol{\theta}} \{\mathcal{L}(\boldsymbol{\theta}) + \lambda_0 \mathcal{R}(\boldsymbol{\theta})\}, \quad \text{subject to} \quad \boldsymbol{\theta}^{(0)} \in \mathcal{C}_T. \tag{A.7}$$

where we define the objective function as $\mathcal{L}(\boldsymbol{\theta}) = \frac{n_T}{N}\mathcal{L}^{(0)}(\boldsymbol{\theta}^{(0)}) + \sum_{k=1}^K \frac{n_S}{N}\mathcal{L}^{(k)}(\boldsymbol{\theta}^{(0)} + \boldsymbol{\theta}^{(k)})$, with the corresponding regularization term $\mathcal{R}(\boldsymbol{\theta}) = \|\boldsymbol{\theta}^{(0)}\|_1 + \sum_{k=1}^K \frac{\lambda_1}{\lambda_0}\|\boldsymbol{\theta}^{(k)}\|_1$. Since there exists a one-to-one mapping between $\boldsymbol{\theta}$ and $\boldsymbol{\beta}$, the solution to the regularized M-estimation problem in (A.7), $\hat{\boldsymbol{\theta}}$, can be transformed into the solution for the original problem (6) without ambiguity. In the subsequent analysis, we first establish the theoretical properties of $\hat{\boldsymbol{\theta}}$ and then use this mapping to derive corresponding results for $\hat{\boldsymbol{\beta}}$.

B.1 Proof of Theorem 1

We begin by introducing some notations. Let S denote the support set of the target coefficient $\boldsymbol{\beta}_*^{(0)}$, and let S^c be its complement. With a slight abuse of notation, we define $\boldsymbol{\theta}_S^{(k)}$ as the sub-vector of $\boldsymbol{\theta}_*^{(k)}$ indexed by S. Throughout the proof, we assume $n_k = n_S$ for $k = 1, \ldots, K$ and $n_k = n_T$ for $k = 0$. Let $\hat{\boldsymbol{\Delta}} := \hat{\boldsymbol{\theta}} - \boldsymbol{\theta}_*$ be the estimation error vector, with its k-th block denoted as $\hat{\boldsymbol{\Delta}}^{(k)} := \hat{\boldsymbol{\theta}}^{(k)} - \boldsymbol{\theta}_*^{(k)}$. Our goal is to establish an upper bound for $\|\hat{\boldsymbol{\Delta}}^{(0)}\|_2^2$, which corresponds to the ℓ_2 estimation error of the target parameter $\boldsymbol{\theta}_*^{(0)} = \boldsymbol{\beta}_*^{(0)}$.

The proof of Theorem 1 relies on three key technical lemmas, whose proofs are provided in Appendix B.5. The first lemma establishes an upper bound for the first-order term of the Taylor series expansion of $\mathcal{L}(\boldsymbol{\theta}_*)$.

Lemma 2 *Under Assumption 1 we choose λ_0 and λ_1 such that $\lambda_0 \geq 2 \left\| \sum_{k=0}^K \frac{n_k}{N} \nabla \mathcal{L}^{(k)}(\boldsymbol{\theta}_*^{(k)}) \right\|_\infty$ and $\lambda_1 \geq 2 \max_{k \in [K]} \left\| \frac{n_S}{N} \nabla \mathcal{L}^{(k)}(\boldsymbol{\theta}_*^{(k)}) \right\|_\infty$, we then have for any*

$$\boldsymbol{\Delta} = \left(\left(\boldsymbol{\Delta}^{(1)}\right)^\top, \ldots, \left(\boldsymbol{\Delta}^{(K)}\right)^\top, \left(\boldsymbol{\Delta}^{(0)}\right)^\top \right)^\top \in \mathbb{R}^{(K+1)p},$$

we have

$$|\langle \nabla \mathcal{L}\left(\boldsymbol{\theta}_*\right), \boldsymbol{\Delta} \rangle| \leq \sum_{k=1}^K \frac{\lambda_1}{2} \left\| \boldsymbol{\Delta}^{(k)} \right\|_1 + \frac{\lambda_0}{2} \left\| \boldsymbol{\Delta}^{(0)} \right\|_1 .$$

The next lemma establishes a restricted set of directions in which $\hat{\boldsymbol{\Delta}}$ lies and the feasibility of the solution $\boldsymbol{\theta}_*$.

Lemma 3 *Under the conditions of Lemma 2 and assume $n_T > 64\left(\tau_0/\alpha_0\right)^2 s \log p$. If we choose $\lambda_T \geq 2\|\nabla \mathcal{L}^{(0)}(\boldsymbol{\theta}_*^{(0)})\|_\infty$, $\kappa_T = 24 s \lambda_T/\alpha_0$, then $\boldsymbol{\theta}_*$ is feasible to problem (6) and*

$$\sum_{k=1}^K \lambda_1 \left\| \hat{\boldsymbol{\Delta}}^{(k)} \right\|_1 + \lambda_0 \left\| \hat{\boldsymbol{\Delta}}^{(0)} \right\|_1 \leq 4\lambda_0 \left\| \hat{\boldsymbol{\Delta}}_S^{(0)} \right\|_1 + 4 \sum_{k=1}^K \lambda_1 h. \tag{A.8}$$

The following lemma ensures a property analogous to restricted strong convexity for $\hat{\boldsymbol{\Delta}}$.

Lemma 4 *Under the conditions of Lemma 3, if $n_S > n_T$, the estimation error $\hat{\boldsymbol{\Delta}}$ satisfies*

$$\begin{aligned} &\mathcal{L}\left(\boldsymbol{\theta}_* + \hat{\boldsymbol{\Delta}}\right) - \mathcal{L}\left(\boldsymbol{\theta}_*\right) - \left\langle \nabla \mathcal{L}\left(\boldsymbol{\theta}_*\right), \hat{\boldsymbol{\Delta}} \right\rangle \\ &\geq \left(\frac{\alpha_{\min}^2}{\gamma_0} - u_n \right) \left\| \hat{\boldsymbol{\Delta}}^{(0)} \right\|_2^2 + \frac{\alpha_{\min}^2}{\gamma_0} \sum_{k=1}^K \frac{n_k}{N} \left\| \hat{\boldsymbol{\Delta}}^{(k)} \right\|_2^2 - \frac{2\alpha_{\max}}{\gamma_0} \sum_{k=1}^K \frac{n_k}{N} \lambda_T \left\| \hat{\boldsymbol{\Delta}}^{(k)} \right\|_1 - v_n \sum_{k=1}^K \lambda_1 h, \end{aligned} \tag{A.9}$$

where

$$\begin{aligned} u_n &= \frac{64(\alpha_{\max}\tau_0 + \beta_{\max}\gamma_0)}{\gamma_0} \frac{n_S}{n_T} \frac{\log p}{N} \cdot \frac{\lambda_0^2 s}{\lambda_1^2 \wedge [(n_S/N)\lambda_0^2]}, \\ v_n &= \frac{64(\alpha_{\max}\tau_0 + \beta_{\max}\gamma_0)}{\gamma_0} \frac{n_S}{n_T} \frac{\log p}{N} \cdot \frac{\sum_{k=1}^K \lambda_1 h}{\lambda_1^2 \wedge [(n_S/N)\lambda_0^2]}, \\ \alpha_{\min} &= \min_{0 \leq k \leq K} \alpha_k, \quad \alpha_{\max} = \max_{0 \leq k \leq K} \alpha_k \text{ and } \quad \beta_{\max} = \max_{0 \leq k \leq K} \beta_k, \end{aligned}$$

with RSC constants (α_k, τ_k) and RSM constants (β_k, γ_k) defined in Assumption 1.

We now proceed to the proof of the theorem. For notational convenience, we first define a optimal value gap function function F as follows:

$$F(\boldsymbol{\Delta}) = \mathcal{L}\left(\boldsymbol{\theta}_* + \boldsymbol{\Delta}\right) - \mathcal{L}\left(\boldsymbol{\theta}_*\right) + \lambda_0 \mathcal{R}\left(\boldsymbol{\theta}_* + \boldsymbol{\Delta}\right) - \lambda_0 \mathcal{R}\left(\boldsymbol{\theta}_*\right),$$

where $\boldsymbol{\theta}_*$ is the transformed model parameter defined in (A.6), and

$$\boldsymbol{\Delta} = \left(\left(\boldsymbol{\Delta}^{(1)}\right)^\top, \ldots, \left(\boldsymbol{\Delta}^{(K)}\right)^\top, \left(\boldsymbol{\Delta}^{(0)}\right)^\top\right)^\top \in \mathbb{R}^{(K+1)p}.$$

By Hölder's inequality and mean value theorem, we have

$$\begin{aligned}
F(\hat{\boldsymbol{\Delta}}) =& \mathcal{L}\left(\boldsymbol{\theta}_* + \hat{\boldsymbol{\Delta}}\right) - \mathcal{L}\left(\boldsymbol{\theta}_*\right) + \lambda_0 \mathcal{R}\left(\boldsymbol{\theta}_* + \hat{\boldsymbol{\Delta}}\right) - \lambda_0 \mathcal{R}\left(\boldsymbol{\theta}_*\right) \\
&\geq - \left\|\nabla \mathcal{L}\left(\boldsymbol{\theta}_*\right)\right\|_\infty \|\hat{\boldsymbol{\Delta}}\|_1 + \hat{\boldsymbol{\Delta}}^\top \nabla^2 \mathcal{L}\left(\boldsymbol{\theta}_* + \phi\hat{\boldsymbol{\Delta}}\right)\hat{\boldsymbol{\Delta}} \quad (\phi \in (0,1)) \\
&\quad + \sum_{k=1}^K \lambda_1 \left(\left\|\boldsymbol{\theta}^{(k)} + \hat{\boldsymbol{\Delta}}^{(k)}\right\|_1 - \left\|\boldsymbol{\theta}^{(k)}\right\|_1\right) + \lambda_0 \left(\left\|\boldsymbol{\theta}_*^{(0)} + \hat{\boldsymbol{\Delta}}^{(0)}\right\|_1 - \left\|\boldsymbol{\theta}_*^{(0)}\right\|_1\right).
\end{aligned}$$

Notice that here we implicitly assume the existence of second-order derivatives; however, the proof remains valid without this assumption, albeit requiring lengthier arguments. Applying Lemma 2, the triangle inequality, and the fact that $\|\boldsymbol{\theta}_{S^c}^{(0)}\|_1 = 0$, $\|\boldsymbol{\theta}_*^{(k)}\|_1 = \|\boldsymbol{\beta}_*^{(k)} - \boldsymbol{\beta}_*^{(0)}\|_1 \leq h$ for $1 \leq k \leq K$, we have for some $\phi \in [0,1]$,

$$\begin{aligned}
F(\hat{\boldsymbol{\Delta}}) \geq& - \sum_{k=1}^K \frac{\lambda_1}{2}\left\|\hat{\boldsymbol{\Delta}}^{(k)}\right\|_1 - \frac{\lambda_0}{2}\left\|\hat{\boldsymbol{\Delta}}^{(0)}\right\|_1 + \hat{\boldsymbol{\Delta}}^\top \nabla^2 \mathcal{L}\left(\boldsymbol{\theta}_* + \phi\hat{\boldsymbol{\Delta}}\right)\hat{\boldsymbol{\Delta}} \\
&+ \sum_{k=1}^K \lambda_1 \left(\left\|\hat{\boldsymbol{\Delta}}^{(k)}\right\|_1 - 2\left\|\boldsymbol{\theta}_*^{(k)}\right\|_1\right) \\
&+ \lambda_0 \left(\left\|\boldsymbol{\theta}_S^{(0)}\right\|_1 - \left\|\hat{\boldsymbol{\Delta}}_S^{(0)}\right\|_1 + \left\|\hat{\boldsymbol{\Delta}}_{S^c}^{(0)}\right\|_1 - \left\|\boldsymbol{\theta}_{S^c}^{(0)}\right\|_1 - \left\|\boldsymbol{\theta}_S^{(0)}\right\|_1 - \left\|\boldsymbol{\theta}_{S^c}^{(0)}\right\|_1\right) \\
\geq& \hat{\boldsymbol{\Delta}}^\top \nabla^2 \mathcal{L}\left(\boldsymbol{\theta}_* + \phi\hat{\boldsymbol{\Delta}}\right)\hat{\boldsymbol{\Delta}} + \frac{\lambda_0}{2}\left(\left\|\hat{\boldsymbol{\Delta}}_{S^c}^{(0)}\right\|_1 - 3\left\|\hat{\boldsymbol{\Delta}}_S^{(0)}\right\|_1\right) + \sum_{k=1}^K \frac{\lambda_1}{2}\left\|\hat{\boldsymbol{\Delta}}^{(k)}\right\|_1 - 2\sum_{k=1}^K \lambda_1 h.
\end{aligned}$$

With Lemma 4, we can obtain a lower bound of the quadratic term $\hat{\boldsymbol{\Delta}}^\top \nabla^2 \mathcal{L}\left(\boldsymbol{\theta}_* + \phi\hat{\boldsymbol{\Delta}}\right)\hat{\boldsymbol{\Delta}}$. Plug in the lower bound, we then obtain

$$\begin{aligned}
F(\hat{\boldsymbol{\Delta}}) \geq& \left(\frac{\alpha_{\min}^2}{\gamma_0} - u_n\right)\left\|\hat{\boldsymbol{\Delta}}^{(0)}\right\|_2^2 + \frac{\alpha_{\min}^2}{\gamma_0}\sum_{k=1}^K \frac{n_S}{N}\left\|\hat{\boldsymbol{\Delta}}^{(k)}\right\|_2^2 - \frac{2\alpha_{\max}}{\gamma_0}\sum_{k=1}^K \frac{n_S}{N}\lambda_T\left\|\hat{\boldsymbol{\Delta}}^{(k)}\right\|_1 - v_n\sum_{k=1}^K \lambda_1 h \\
&+ \frac{\lambda_0}{2}\left(\left\|\hat{\boldsymbol{\Delta}}_{S^c}^{(0)}\right\|_1 - 3\left\|\hat{\boldsymbol{\Delta}}_S^{(0)}\right\|_1\right) + \sum_{k=1}^K \frac{\lambda_1}{2}\left\|\hat{\boldsymbol{\Delta}}^{(k)}\right\|_1 - 2\sum_{k=1}^K \lambda_1 h \\
\geq& \left(\frac{\alpha_{\min}^2}{\gamma_0} - u_n\right)\left\|\hat{\boldsymbol{\Delta}}^{(0)}\right\|_2^2 - \frac{3\sqrt{s}\lambda_0}{2}\left\|\hat{\boldsymbol{\Delta}}^{(0)}\right\|_2 \\
&+ \left(\sum_{k=1}^K \frac{\lambda_1}{2} - \frac{2\alpha_{\max}}{\gamma_0}\sum_{k=1}^K \frac{n_S}{N}\lambda_T\right)\left\|\hat{\boldsymbol{\Delta}}^{(k)}\right\|_1 - (2 + v_n)\sum_{k=1}^K \lambda_1 h. \qquad \text{(A.10)}
\end{aligned}$$

Recall that we choose $\lambda_1 \geq 4\frac{\alpha_{\max}}{K\gamma_0}\lambda_T \geq 4\frac{\alpha_{\max}}{\gamma_0}\frac{n_S}{N}\lambda_T$. From Lemma 3, we have $\boldsymbol{\theta}_*$ is feasible to problem (6a). Since $\hat{\boldsymbol{\theta}}$ is the solution to the problem (A.7), then we have $F(\hat{\boldsymbol{\Delta}}) \leq F(\mathbf{0}) = 0$. These results, combining with (A.10), lead to

$$0 \geq \left(\frac{\alpha_{\min}^2}{\gamma_0} - u_n\right) \left\|\hat{\boldsymbol{\Delta}}^{(0)}\right\|_2^2 - \frac{3\sqrt{s}\lambda_0}{2} \left\|\hat{\boldsymbol{\Delta}}^{(0)}\right\|_2 - (2 + v_n)\sum_{k=1}^{K} \lambda_1 h, \tag{A.11}$$

which is an inequality quadratic in $\|\hat{\boldsymbol{\Delta}}^{(0)}\|_2$, we can subsequently establish the convergence rate as

$$\|\hat{\boldsymbol{\Delta}}^{(0)}\|_2^2 \leq \frac{1}{(\alpha_{\min}^2/\gamma_0 - u_n)^2}\left(\frac{9}{4}s\lambda_0^2 + 2(\alpha_{\min}^2/\gamma_0 - u_n)(2 + v_n)\sum_{k=1}^{K}\lambda_1 h\right). \tag{A.12}$$

Recall the definition of u_n and v_n in Lemma 4. Under the condition

$$n_S > n_T > \left[\frac{C_0(2\lambda_0^2 s + K\lambda_1 h/\gamma_0)}{(K\lambda_1^2) \wedge \lambda_0^2} + \frac{64\tau_0^2 s}{\alpha_0^2}\right]\log p,$$

imposed in Theorem 1, we have $u_n \leq \alpha_{\min}^2/(2\gamma_0)$ and $v_n \leq 1$. Therefore bound (A.12) leads to the first term of the bound in (7).

To finish the proof of Theorem 1, it remains to show the second term in the bound (7). Recall that we impose the constraint

$$\mathcal{C}_T = \{\boldsymbol{\beta}^{(0)} \in \mathbb{R}^p : \|\nabla\mathcal{L}^{(0)}(\boldsymbol{\beta}^{(0)})\|_\infty \leq \lambda_T, \|\boldsymbol{\beta}^{(0)}\|_1 \leq \|\hat{\boldsymbol{\beta}}_T^{(0)}\|_1 + \kappa_T\}, \tag{A.13}$$

on the solution of problem (6) with

$$\hat{\boldsymbol{\beta}}_T^{(0)} = \operatorname*{argmin}_{\boldsymbol{\beta}\in\mathbb{R}^p}\left\{\mathcal{L}^{(0)}(\boldsymbol{\beta}) + \lambda_T\|\boldsymbol{\beta}\|_1\right\}.$$

Notice that $\boldsymbol{\beta}_{S^c}^{(0)} = \mathbf{0}$ and $\|\boldsymbol{\beta}_*^{(0)}\|_1 = \|\boldsymbol{\beta}_S^{(0)}\|_1$ (for simplicity, we omit the subscript $*$ in $\boldsymbol{\beta}_S^{(0)}$). From the optimality condition of (6), we have $\hat{\boldsymbol{\beta}}^{(0)} \in \mathcal{C}_T$ and thus $\|\hat{\boldsymbol{\beta}}^{(0)}\|_1 \leq \|\hat{\boldsymbol{\beta}}_T^{(0)}\|_1 + \kappa_T$. Then with triangle inequality we can show that

$$\begin{aligned}
\|\boldsymbol{\beta}_*^{(0)}\|_1 &\geq \|\hat{\boldsymbol{\beta}}_T^{(0)}\|_1 - \|\boldsymbol{\beta}_*^{(0)} - \hat{\boldsymbol{\beta}}_T^{(0)}\|_1 \geq \|\hat{\boldsymbol{\beta}}^{(0)}\|_1 - \|\boldsymbol{\beta}_*^{(0)} - \hat{\boldsymbol{\beta}}_T^{(0)}\|_1 - \kappa_T \\
&\geq \left(\|\hat{\boldsymbol{\beta}}_S^{(0)}\|_1 + \|\hat{\boldsymbol{\beta}}_{S^c}^{(0)} - \boldsymbol{\beta}_{S^c}^{(0)}\|_1\right) - \|\boldsymbol{\beta}_*^{(k)} - \hat{\boldsymbol{\beta}}_T^{(0)}\|_1 - \kappa_T \\
&\geq \left[\left(\|\boldsymbol{\beta}_*^{(0)}\|_1 - \|\hat{\boldsymbol{\beta}}_S^{(0)} - \boldsymbol{\beta}_S^{(0)}\|_1\right) + \|\hat{\boldsymbol{\beta}}_{S^c}^{(0)} - \boldsymbol{\beta}_{S^c}^{(0)}\|_1\right] - \|\boldsymbol{\beta}_*^{(0)} - \hat{\boldsymbol{\beta}}_T^{(0)}\|_1 - \kappa_T \\
&= \left[\left(\|\boldsymbol{\beta}_*^{(0)}\|_1 - \|\hat{\boldsymbol{\Delta}}_S^{(0)}\|_1\right) + \|\hat{\boldsymbol{\Delta}}_{S^c}^{(0)}\|_1\right] - \|\boldsymbol{\beta}_*^{(0)} - \hat{\boldsymbol{\beta}}_T^{(0)}\|_1 - \kappa_T,
\end{aligned}$$

where recall that we denote $\hat{\boldsymbol{\Delta}}^{(0)} = \hat{\boldsymbol{\theta}}^{(0)} - \boldsymbol{\theta}_*^{(0)} = \hat{\boldsymbol{\beta}}^{(0)} - \boldsymbol{\beta}_*^{(0)}$.

According to (A.27) in the proof of Lemma 3, we have $\|\boldsymbol{\beta}_*^{(0)} - \hat{\boldsymbol{\beta}}_T^{(0)}\|_1 \leq \kappa_T$. Reorganizing the terms and applying Cauchy–Schwarz inequality yields

$$\begin{aligned}
\|\hat{\boldsymbol{\Delta}}^{(0)}\|_1 &= \|\hat{\boldsymbol{\Delta}}_S^{(0)}\|_1 + \|\hat{\boldsymbol{\Delta}}_{S^c}^{(0)}\|_1 \\
&\leq 2\|\hat{\boldsymbol{\Delta}}_S^{(0)}\|_1 + \|\boldsymbol{\beta}_*^{(0)} - \hat{\boldsymbol{\beta}}_T^{(0)}\|_1 + \kappa_T \\
&\leq 2\sqrt{s}\|\hat{\boldsymbol{\Delta}}_S^{(0)}\|_2 + 2\kappa_T \\
&\leq 2\sqrt{s}\|\hat{\boldsymbol{\Delta}}^{(0)}\|_2 + 2\kappa_T, \qquad \text{(A.14)}
\end{aligned}$$

where recall $s := |S|$ is the cardinality of set S.

Recall that we choose $\lambda_T \geq 2\|\nabla\mathcal{L}^{(0)}(\boldsymbol{\beta}_*^{(0)})\|_\infty$ and have shown that $\boldsymbol{\beta}_*^{(0)} \in \mathcal{C}_T$. Hence, we have

$$\|\nabla\mathcal{L}^{(0)}(\hat{\boldsymbol{\beta}}^{(0)}) - \nabla\mathcal{L}^{(0)}(\boldsymbol{\beta}_*^{(0)})\|_\infty \leq \|\langle\nabla\mathcal{L}^{(0)}(\hat{\boldsymbol{\beta}}^{(0)})\|_\infty + \|\langle\nabla\mathcal{L}^{(0)}(\boldsymbol{\beta}_*^{(0)})\|_\infty \leq 2\lambda_T,$$

which together with Holder's inequality implies

$$\langle\nabla\mathcal{L}^{(0)}(\hat{\boldsymbol{\beta}}^{(0)}) - \nabla\mathcal{L}^{(0)}(\boldsymbol{\beta}_*^{(0)}), \hat{\boldsymbol{\Delta}}^{(0)}\rangle \leq 2\lambda_T\|\hat{\boldsymbol{\Delta}}^{(0)}\|_1.$$

Applying the RSC condition in Assumption 1 to the left-hand side of the inequality leads to

$$\alpha_0\|\hat{\boldsymbol{\Delta}}^{(0)}\|_2^2 - \tau_0\frac{\log p}{n_T}\|\hat{\boldsymbol{\Delta}}^{(0)}\|_1^2 \leq 2\lambda_T\|\hat{\boldsymbol{\Delta}}^{(0)}\|_1.$$

Now plugging in the results in (A.14), the choice of κ_T and applying the ℓ_1 error bound in (A.26), we can show that

$$\left(\alpha_0 - u_n'\right)\|\hat{\boldsymbol{\Delta}}^{(0)}\|_2^2 - 4\sqrt{s}\lambda_T\|\hat{\boldsymbol{\Delta}}^{(0)}\|_2 - 4\left(\frac{24}{\alpha_0} + v_n'\right)s\lambda_T^2, \qquad \text{(A.15)}$$

with

$$u_n' = 8\tau_0 s\frac{\log p}{n_T} \text{ and } v_n' = \frac{24^2\tau_0}{\alpha_0^2}\frac{s\log p}{n_T}. \qquad \text{(A.16)}$$

Under the choice of n_T specified in Theorem 1, we have $u_n' \leq \alpha_0/2$ and $v_n' \leq 24/\alpha_0$. Therefore, (A.15) as a quadratic inequality in $\|\hat{\boldsymbol{\Delta}}^{(0)}\|_2$ and solving for its upper bound yields the second term in (7). The proof is then finished.

B.2 Proof of Corollary 1

Applying the Hoeffding-type inequality (cf. Proposition 5.1 in Vershynin (2012)) with Assumption 2 and $n_S > n_T \gtrsim \log p$, we have $\|\nabla\mathcal{L}^{(0)}(\boldsymbol{\beta}_*^{(0)})\|_\infty \lesssim \sqrt{\log p/n_T}$,

$$\Big\|\frac{n_T}{N}\nabla\mathcal{L}^{(0)}(\boldsymbol{\beta}_*^{(0)}) + \sum_{k=1}^K\frac{n_k}{N}\nabla\mathcal{L}^{(k)}(\boldsymbol{\beta}_*^{(k)})\Big\|_\infty \lesssim \sqrt{\frac{\log p}{N}}, \text{ and } \Big\|\nabla\mathcal{L}^{(k)}(\boldsymbol{\beta}_*^{(k)})\Big\|_\infty \lesssim \sqrt{\frac{\log p}{n_S}},$$

with probability larger than $1 - c_1\exp(-c_2\log p)$. Therefore, the choice of tuning parameters in Corollary 1 satisfies the requirement stated in Theorem 1, thereby we can directly plug

the choice of tuning parameters into the bound (7) to obtain the probabilistic bound. It remains to verify that the conditions on n_T and n_S satisfy the requirements of Theorem 1. To establish the first term of the bound in (7), it suffices to show that u_n and v_n from (A.12) and u'_n and v'_n from (A.16) are all of order $o(1)$ when $h\sqrt{\frac{\log p}{n_T}} \lesssim \frac{s\log p}{n_T}$.

Under the assumption $n_T \gg s\log p$, we immediately obtain $u'_n = o(1)$ and $v'_n = o(1)$. It remains to establish the same for u_n and v_n. We proceed by discussing different cases based on the order of λ_0. Recall the tuning parameter choices given in the theorem:

$$\lambda_0 = c_0\left[\Big(\frac{h^2\log p}{s^2 n_T}\Big)^{1/4} + \Big(\frac{\log p}{N}\Big)^{1/2}\right], \quad \lambda_1 = c_0\frac{n_S}{N}\Big(\frac{\log p}{n_T}\Big)^{1/2}.$$

Case 1: If $\frac{s\log p}{N} \lesssim h\sqrt{\frac{\log p}{n_T}} \lesssim \frac{s\log p}{n_T}$, then we have $\lambda_0 \asymp \left(\frac{h^2\log p}{s^2 n_T}\right)^{1/4}$ and $\lambda_1 \asymp \frac{n_S}{N}\sqrt{\frac{\log p}{n_T}}$. Recalling that we set $n_k = n_S$ for $k \in [K]$, we consider the following two cases:

Case 1.1: If $\lambda_1^2 \lesssim \frac{n_S}{N}\lambda_0^2$, by the assumption that $Ks\frac{\log p}{n_T} = o(1)$, we have

$$u_n \lesssim \frac{1}{K}\frac{\log p}{n_T}\frac{\lambda_0^2 s}{\lambda_1^2} \lesssim Kh\sqrt{\frac{\log p}{n_T}} \lesssim Ks\frac{\log p}{n_T} = o(1),$$

where in the first inequality we use the fact that $n_S/N \geq 1/K$ and for the last inequality we use the fact that $h \lesssim s\sqrt{\frac{\log p}{n_T}}$. Similarly, we can establish that

$$v_n \lesssim \frac{1}{K}\frac{\log p}{n_T}\frac{\left(\sum_{k=1}^K \lambda_1 h\right)}{\lambda_1^2} \lesssim \sqrt{\frac{\log p}{n_T}}\sum_{k=1}^K h \lesssim Ks\frac{\log p}{n_T} = o(1).$$

Case 1.2: If $\lambda_1^2 \gtrsim \frac{n_S}{N}\lambda_0^2$, we can similarly establish that $u_n \lesssim \frac{s\log p}{n_T} = o(1)$, which follows from the assumptions that $\frac{s\log p}{n_T} = o(1)$. In this case, noting that $\sum_{k=1}^K \lambda_1 h \lesssim Kh\sqrt{\frac{\log p}{n_T}}$, thereby we have

$$v_n \lesssim \frac{1}{K}\frac{\log p}{n_T}\frac{\sum_{k=1}^K \lambda_1 h}{\frac{n_S}{N}\lambda_0^2} \lesssim \frac{s\log p}{n_T} = o(1).$$

Case 2: If $\frac{s\log p}{N} \gtrsim h\sqrt{\frac{\log p}{n_T}}$, then we have $\lambda_0 \asymp \sqrt{\frac{\log p}{N}}$ and $\lambda_1 \asymp \frac{n_S}{N}\sqrt{\frac{\log p}{n_T}}$. In this case, we have $\lambda_1^2 \gtrsim (n_S/N)\lambda_0^2$ as $n_S > n_T$. So following the discussion in the first case, we have

$$u_n \lesssim \frac{1}{K}\frac{\log p}{n_T}sK = \frac{s\log p}{n_T} = o(1).$$

Further notice that in this case, it holds that $\sum_{k=1}^K \lambda_1 h \lesssim h\sqrt{\frac{\log p}{n_T}} \lesssim \frac{s\log p}{N}$. Therefore, we further obtain

$$v_n \lesssim \frac{1}{K}\frac{\log p}{n_T}\frac{\sum_{k=1}^K \lambda_1 h}{(n_S/N)\lambda_0^2} \lesssim \frac{1}{K}\frac{\log p}{n_T}sK \lesssim \frac{s\log p}{n_T} = o(1).$$

The proof is then finished.

B.3 Proof of Corollary 2

When the tuning parameters are chosen as in (A.3), Corollary 1 already yields the desired rate. Thus, it suffices to consider the remaining case (A.4), where we show that $\hat{\boldsymbol{\beta}}^{(0)} = \hat{\boldsymbol{\beta}}_{\text{Pooling}}$. Then an application of Lemma 1 in Li et al. (2022) and Theorem 1 in Negahban et al. (2012) yield

$$\|\hat{\boldsymbol{\beta}}^{(0)} - \boldsymbol{\beta}_*^{(0)}\|_2^2 = \|\hat{\boldsymbol{\beta}}_{\text{Pooling}} - \boldsymbol{\beta}_*^{(0)}\|_2^2 \lesssim \frac{s\log p}{N} + C_\Sigma^2 h^2.$$

Notice that for problem (6), the solution for source parameter, $\hat{\boldsymbol{\beta}}^{(k)}$, satisfies

$$\hat{\boldsymbol{\beta}}^{(k)} \in \operatorname*{argmin}_{\boldsymbol{\beta}\in\mathbb{R}^p} \left\{ \frac{n_S}{N}\mathcal{L}^{(k)}(\boldsymbol{\beta}) + \lambda_1\|\boldsymbol{\beta} - \hat{\boldsymbol{\beta}}^{(0)}\|_1 \right\}. \tag{A.17}$$

For any $\boldsymbol{\beta} \neq \hat{\boldsymbol{\beta}}^{(0)}$, by the convexity of $\mathcal{L}^{(k)}(\cdot)$ and Hölder's inequality, we have

$$\begin{aligned}
\frac{n_S}{N}\mathcal{L}^{(k)}(\boldsymbol{\beta}) + \lambda_1\left\|\hat{\boldsymbol{\beta}}^{(0)} - \boldsymbol{\beta}\right\|_1 \geq & \frac{n_S}{N}\mathcal{L}^{(k)}(\hat{\boldsymbol{\beta}}^{(0)}) + \left\langle \frac{n_S}{N}\nabla\mathcal{L}^{(k)}(\hat{\boldsymbol{\beta}}^{(0)}), \boldsymbol{\beta} - \hat{\boldsymbol{\beta}}^{(0)} \right\rangle + \lambda_1\left\|\hat{\boldsymbol{\beta}}^{(0)} - \boldsymbol{\beta}\right\|_1 \\
\geq & \frac{n_S}{N}\mathcal{L}^{(k)}(\hat{\boldsymbol{\beta}}^{(0)}) - \left\|\frac{n_S}{N}\nabla\mathcal{L}^{(k)}(\hat{\boldsymbol{\beta}}^{(0)})\right\|_\infty \left\|\hat{\boldsymbol{\beta}}^{(0)} - \boldsymbol{\beta}\right\|_1 + \lambda_1\left\|\hat{\boldsymbol{\beta}}^{(0)} - \boldsymbol{\beta}\right\|_1 \\
= & \frac{n_S}{N}\mathcal{L}^{(k)}(\hat{\boldsymbol{\beta}}^{(0)}) + \left(\lambda_1 - \left\|\frac{n_S}{N}\nabla\mathcal{L}^{(k)}(\hat{\boldsymbol{\beta}}^{(0)})\right\|_\infty\right)\left\|\hat{\boldsymbol{\beta}}^{(0)} - \boldsymbol{\beta}\right\|_1.
\end{aligned}$$

Therefore, if $\|\frac{n_S}{N}\nabla\mathcal{L}^{(k)}(\hat{\boldsymbol{\beta}}^{(0)})\|_\infty < \lambda_1$, then $\hat{\boldsymbol{\beta}}^{(0)}$ is the unique minimizer of problem (A.17), which further implies that $\hat{\boldsymbol{\beta}}^{(k)} = \hat{\boldsymbol{\beta}}^{(0)} = \hat{\boldsymbol{\beta}}_{\text{Pooling}}$ for all $k \in [K]$. The proof is then finished.

B.4 Proof of Theorem 2

Recall that we have the decomposition

$$\tilde{\boldsymbol{\beta}}^{(k)} - \boldsymbol{\beta}_*^{(k)} = -\hat{\boldsymbol{\Theta}}^{(k)}\nabla\mathcal{L}^{(k)}(\boldsymbol{\beta}_*^{(k)}) + \underbrace{\left(\boldsymbol{I} - \hat{\boldsymbol{\Theta}}^{(k)}\nabla^2\mathcal{L}^{(k)}(\hat{\boldsymbol{\beta}}_{\text{Int}}^{(k)})\right)\left(\hat{\boldsymbol{\beta}}_L^{(k)} - \boldsymbol{\beta}_*^{(k)}\right)}_{\boldsymbol{b}^{(k)}}.$$

We first show a Lemma discussing the bias and variance components in (14), whose proof is based on van de Geer et al. (2014) but taking into account that $\boldsymbol{\beta}_*^{(k)}$ is not exactly s-sparse (due to the difference term $\boldsymbol{\delta}^{(k)}$).

Lemma 5 *Under Assumption 1 and 2 and* $\frac{s\log p}{n_S} + h\sqrt{\frac{\log p}{n_S}} = o(1)$, *if we construct* $\{\hat{\boldsymbol{\beta}}_L^{(k)}\}_{k\in[K]}$ *as the local lasso estimators defined in Section 4 and* $\{\hat{\boldsymbol{\Theta}}^{(k)}\}_{k\in[K]}$ *using (A.5), with parameters* $\tilde{\lambda}_k = \mu_{jk} \asymp \sqrt{\frac{\log p}{n_S}}$, *then we have that for* $k = 1, \ldots, K$,

$$\left\|\hat{\boldsymbol{\Theta}}^{(k)}\nabla\mathcal{L}^{(k)}(\boldsymbol{\beta}_*^{(k)})\right\|_\infty \lesssim \sqrt{\frac{\log p}{n_S}} + h\sqrt{\frac{\log p}{n_S}}, \tag{A.18}$$

and

$$\left\|\boldsymbol{b}^{(k)}\right\|_\infty \lesssim \frac{s\log p}{n_S} + h\sqrt{\frac{\log p}{n_S}}, \tag{A.19}$$

with probability larger than $1 - c_1\exp(-c_2\log p)$.

Now we proceed to the proof of the theorem. We first show that by reparametrization, problem (11) is essentially a special case of problem (6). Then we apply techniques similar to those used in Theorem 1 to prove the results.

Following the arguments in the proof of Theorem 1, we again adopt the reparametrization in (A.6) and reformulate problem (11) as

$$\hat{\boldsymbol{\theta}}_D \in \underset{\boldsymbol{\theta}}{\operatorname{argmin}}\{\tilde{\mathcal{L}}(\boldsymbol{\theta}) + \lambda_0\mathcal{R}(\boldsymbol{\theta})\}, \quad \text{subject to} \quad \boldsymbol{\theta}^{(0)} \in \mathcal{C}_T. \tag{A.20}$$

where we define $\tilde{\mathcal{L}}(\boldsymbol{\theta}) := \frac{n_T}{N}\mathcal{L}^{(0)}(\boldsymbol{\theta}^{(0)}) + \sum_{k=1}^K \frac{n_S}{N}\tilde{\mathcal{L}}^{(k)}(\boldsymbol{\theta}^{(0)} + \boldsymbol{\theta}^{(k)})$ and $\mathcal{R}(\boldsymbol{\theta}) := \left\|\boldsymbol{\theta}^{(0)}\right\|_1 + \sum_{k=1}^K \frac{\lambda_1}{\lambda_0}\left\|\boldsymbol{\theta}^{(k)}\right\|_1$, with $\tilde{\mathcal{L}}^{(k)}(\boldsymbol{\beta}) := \frac{n_S}{2}\|\tilde{\boldsymbol{\beta}}^{(k)} - \boldsymbol{\beta}\|_2^2$.

Following the aforementioned reformulation, we can employ an approach similar to that used in Lemma 2 to establish the result about $\left\langle\nabla\tilde{\mathcal{L}}(\boldsymbol{\theta}_*), \boldsymbol{\Delta}\right\rangle$. Define $\delta_k = \frac{s\log p}{N} + \frac{n_S}{N}\sqrt{\frac{\log p}{n_S}}h$ and $\delta_0 = \frac{Ks\log p}{N} + \sum_{k=1}^K \frac{n_S}{N}\sqrt{\frac{\log p}{n_S}}h$, then the result is stated as follows:

Lemma 6 *Under the conditions in Lemma 5, if we choose* $\lambda_1 = c_k\left(\sqrt{\frac{n_S}{N}\frac{\log p}{N}} + \delta_k\right)$ *and* $\lambda_0 = c_0\left(\sqrt{\frac{\log p}{N}} + \delta_0\right)$ *for some appropriate constants* $c_1, \ldots, c_K$, *then we have for any* $\boldsymbol{\Delta} = \left(\left(\boldsymbol{\Delta}^{(0)}\right)^\top, \left(\boldsymbol{\Delta}^{(1)}\right)^\top, \ldots, \left(\boldsymbol{\Delta}^{(K)}\right)^\top\right)^\top \in \mathbb{R}^{(K+1)p}$,

$$\left|\left\langle\nabla\tilde{\mathcal{L}}(\boldsymbol{\theta}_*), \boldsymbol{\Delta}\right\rangle\right| \le \sum_{k=1}^K \frac{\lambda_1}{2}\left\|\boldsymbol{\Delta}^{(k)}\right\|_1 + \frac{\lambda_0}{2}\left\|\boldsymbol{\Delta}^{(0)}\right\|_1.$$

with probability larger than $1 - c_1\exp(-c_2\log p)$.

One may notice that the only difference between Lemma 6 and Lemma 2 is the choice of λ_0 and λ_1. With this new choice of parameters, it is easy to verify that $\lambda_1 \gtrsim \frac{n_S}{N}\lambda_T$ still holds and the arguments used in the proof of Lemma 3, Lemma 4 and Theorem 1 remain applicable for the new problem (11).

Hence, following similar procedures, we obtain

$$\|\hat{\boldsymbol{\Delta}}^{(0)}\|_2^2 \le \frac{1}{(\alpha_{\min}^2/\gamma_0 - u_n)^2}\left(\frac{9}{4}s\lambda_0^2 + 2(\alpha_{\min}^2/\gamma_0 - u_n)(2 + v_n)\sum_{k=1}^K \lambda_1 h\right)$$

To prove the theorem, it remains to show that $u_n = o(1)$ and $v_n = o(1)$ under the new set of choice of tuning parameters. Notice that, similar to the proof of Theorem 1, we only

need to prove the result under the regime when $h\sqrt{\frac{\log p}{n_T}} \lesssim \frac{s\log p}{n_T}$. Recall that the tuning parameter choices

$$\lambda_0 \asymp \Big(\frac{h^2\log p}{s^2 n_T}\Big)^{1/4} + \Big(\frac{\log p}{N}\Big)^{1/2} + \frac{Ks\log p}{N} + \sum_{k=1}^{K}\frac{n_S}{N}\sqrt{\frac{\log p}{n_S}}h,$$
$$\lambda_1 \asymp \frac{n_S}{N}\Big(\frac{\log p}{n_T}\Big)^{1/2} + \frac{s\log p}{N} + \frac{n_S}{N}\sqrt{\frac{\log p}{n_S}}h.$$

We consider the following two cases:

Case 1: If $\lambda_1^2 \lesssim \frac{n_S}{N}\lambda_0^2$, then we have

$$\begin{aligned} u_n &\lesssim \frac{1}{K}\frac{\log p}{n_T}\frac{\lambda_0^2 s}{\lambda_1^2} \lesssim \frac{1}{K}\frac{s\log p}{n_T}\frac{\frac{h\sqrt{\log p}}{s\sqrt{n_T}} + \frac{\log p}{N} + \Big(\frac{Ks\log p}{N}\Big)^2 + \frac{\log p}{n_S}h^2}{\frac{1}{K^2}\frac{\log p}{n_T} + \Big(\frac{s\log p}{N}\Big)^2 + \frac{1}{K^2}\frac{\log p}{n_S}h^2} \\ &\lesssim \frac{1}{K}\frac{s\log p}{n_T}\frac{\frac{h\sqrt{\log p}}{s\sqrt{n_T}} + \frac{\log p}{N} + \Big(\frac{Ks\log p}{N}\Big)^2 + \frac{\log p}{n_S}h^2}{\frac{1}{K^2}\frac{\log p}{n_T}} \\ &\lesssim Kh\sqrt{\frac{\log p}{n_T}} + \frac{s\log p}{n_S} + \frac{Ks^2\log^2 p}{n_S^2} + \frac{Ks\log p}{n_S}h^2 = o(1) \end{aligned} \tag{A.21}$$

Here, for the last inequality we use the assumption that $n_T \gg \big((K^2h^2)\vee s\big)\log p$ and $n_S \gg (K^2h^4 + K^2h^2 + Kh^2 s + Khs + Ks + sh^2 + s^{3/2})\log p$, which are obtained from the assumption $n_T \gg K^2 s\log p$ and $n_S \gg K^2 s^2\log p$ in Theorem 2 and the fact that $h \lesssim s\sqrt{\frac{\log p}{n_T}}$ under this regime. Similarly, we can establish that

$$v_n \lesssim \frac{1}{K}\frac{\log p}{n_T}\frac{\sqrt{\frac{\log p}{n_T}}h + \frac{s\log p}{n_S}h + \sqrt{\frac{\log p}{n_S}}h^2}{\frac{1}{K^2}\frac{\log p}{n_T}} \lesssim K\sqrt{\frac{\log p}{n_T}}h + \frac{Ks\log p}{n_S}h + K\sqrt{\frac{\log p}{n_S}}h^2 = o(1).$$

Case 2: If $\lambda_1^2 \gtrsim \frac{n_S}{N}\lambda_0^2$, we can similarly establish that $u_n \lesssim \frac{s\log p}{n_T} = o(1)$. For v_n, one can show that

$$\begin{aligned} v_n &\lesssim \frac{1}{K}\frac{\log p}{n_T}\frac{\sum_{k=1}^K \lambda_1 h}{\frac{n_S}{N}\lambda_0^2} \lesssim \frac{Kh\log p}{n_T}\frac{\lambda_1}{\lambda_0^2} \lesssim \frac{Kh\log p}{n_T}\frac{\frac{n_S}{N}\Big(\frac{\log p}{n_T}\Big)^{1/2} + \frac{s\log p}{N} + \frac{n_S}{N}\sqrt{\frac{\log p}{n_S}}h}{\frac{h\sqrt{\log p}}{s\sqrt{n_T}}} \\ &\lesssim \frac{s\log p}{n_T} + \frac{s^{3/2}\log p}{n_S}\sqrt{\frac{s\log p}{n_T}} + \sqrt{\frac{s\log p}{n_T}}\sqrt{\frac{s\log p}{n_S}}h = o(1). \end{aligned} \tag{A.22}$$

The proof is then finished.

B.5 Proof of Lemmas

Proof of Lemma 1 Notice that by triangle inequality, we obtain

$$\Big\|\frac{n_S}{N}\nabla\mathcal{L}^{(k)}(\hat{\boldsymbol\beta}_{\text{Pooling}})\Big\|_\infty \le \Big\|\frac{n_S}{N}\nabla\mathcal{L}^{(k)}(\boldsymbol\beta_*^{(k)})\Big\|_\infty + \Big\|\frac{n_S}{N}\Big(\nabla\mathcal{L}^{(k)}(\hat{\boldsymbol\beta}_{\text{Pooling}}) - \nabla\mathcal{L}^{(k)}(\boldsymbol\beta_*^{(k)})\Big)\Big\|_\infty.$$

Since we assume $n_S \gtrsim \log p$, by Assumption 2 and the Hoeffding-type inequality (cf. Proposition 5.1 in Vershynin (2012)) we have $\|\frac{n_S}{N}\nabla\mathcal{L}^{(k)}(\boldsymbol{\beta}_*^{(k)})\|_\infty \lesssim \frac{n_S}{N}\sqrt{\frac{\log p}{n_S}}$ with probability larger than $1-c_1\exp(-c_2\log p)$. By the ℓ_∞ curvature assumption imposed in the Lemma and triangle inequality, we have

$$\left\|\nabla\mathcal{L}^{(k)}(\hat{\boldsymbol{\beta}}_{\text{Pooling}})-\nabla\mathcal{L}^{(k)}(\boldsymbol{\beta}_*^{(k)})\right\|_\infty \leq \eta_k\left\|\hat{\boldsymbol{\beta}}_{\text{Pooling}}-\boldsymbol{\beta}_*^{(k)}\right\|_\infty+\zeta_k\sqrt{\frac{\log p}{n_S}}\left\|\hat{\boldsymbol{\beta}}_{\text{Pooling}}-\boldsymbol{\beta}_*^{(k)}\right\|_1, \tag{A.23}$$

with probability larger than $1-c_1\exp(-c_2\log p)$.

Another application of Lemma 1 in Li et al. (2022) and Theorem 1 in Negahban et al. (2012) leads to

$$\|\hat{\boldsymbol{\beta}}_{\text{Pooling}}-\boldsymbol{\beta}_*^{(k)}\|_\infty \lesssim \sqrt{\frac{\log p}{N}}+\|\boldsymbol{\beta}_{\text{Pooling}}-\boldsymbol{\beta}_*^{(k)}\|_\infty, \text{ and } \|\hat{\boldsymbol{\beta}}_{\text{Pooling}}-\boldsymbol{\beta}_*^{(k)}\|_1 \lesssim s\sqrt{\frac{\log p}{N}}+C_\Sigma h. \tag{A.24}$$

Recall that we define $K_P=\max_{k\in[K]}\|\boldsymbol{\beta}_{\text{Pooling}}-\boldsymbol{\beta}_*^{(k)}\|_\infty$. Plug the results in (A.24) back into (A.23) finishes the proof.

Proof of Lemma 2 By definition, we have

$$\nabla\mathcal{L}(\boldsymbol{\theta}_*)=\left(\frac{n_S}{N}\nabla\mathcal{L}^{(1)}(\boldsymbol{\beta}_*^{(1)}),\ldots,\frac{n_S}{N}\nabla\mathcal{L}^{(K)}(\boldsymbol{\beta}_*^{(K)}),\frac{n_T}{N}\nabla\mathcal{L}^{(0)}(\boldsymbol{\beta}_*^{(0)})+\sum_{k=1}^K\frac{n_S}{N}\nabla\mathcal{L}^{(k)}(\boldsymbol{\beta}_*^{(k)})\right)^\top$$

Therefore, by Hölder's inequality, we have

$$\begin{aligned}|\langle\nabla\mathcal{L}(\boldsymbol{\theta}_*),\boldsymbol{\Delta}\rangle| &= \sum_{k=1}^K\left|\left\langle\frac{n_S}{N}\nabla\mathcal{L}^{(k)}(\boldsymbol{\beta}_*^{(k)}),\boldsymbol{\Delta}^{(k)}\right\rangle\right|+\left|\left\langle\sum_{k=0}^K\frac{n_k}{N}\nabla\mathcal{L}^{(k)}(\boldsymbol{\beta}_*^{(k)}),\boldsymbol{\Delta}^{(0)}\right\rangle\right|\\ &\leq \sum_{k=1}^K\left\|\frac{n_S}{N}\nabla\mathcal{L}^{(k)}(\boldsymbol{\beta}_*^{(k)})\right\|_\infty\left\|\boldsymbol{\Delta}^{(k)}\right\|_1+\left\|\sum_{k=0}^K\frac{n_k}{N}\nabla\mathcal{L}^{(k)}(\boldsymbol{\beta}_*^{(k)})\right\|_\infty\left\|\boldsymbol{\Delta}^{(0)}\right\|_1.\end{aligned}$$

Therefore, as long as we choose λ_0 and λ_1 such that $\lambda_0 \geq 2\left\|\sum_{k=0}^K\frac{n_k}{N}\nabla\mathcal{L}^{(k)}(\boldsymbol{\beta}_*^{(k)})\right\|_\infty$ and $\lambda_1\geq 2\max_{k\in[K]}\left\|\frac{n_S}{N}\nabla\mathcal{L}^{(k)}(\boldsymbol{\beta}_*^{(k)})\right\|_\infty$, we then have

$$|\langle\nabla\mathcal{L}(\boldsymbol{\theta}_*),\boldsymbol{\Delta}\rangle| \leq \sum_{k=1}^K\frac{\lambda_1}{2}\left\|\boldsymbol{\Delta}^{(k)}\right\|_1+\frac{\lambda_0}{2}\left\|\boldsymbol{\Delta}^{(0)}\right\|_1,$$

as claimed.

B.6 Proof of Lemma 3

We first establish the feasibility of $\boldsymbol{\theta}_*$ for the problem (A.7). Recall that we impose the constraint

$$\mathcal{C}_T=\{\boldsymbol{\beta}^{(0)}\in\mathbb{R}^p:\|\nabla\mathcal{L}^{(0)}(\boldsymbol{\beta}^{(0)})\|_\infty\leq\lambda_T,\|\boldsymbol{\beta}^{(0)}\|_1\leq\|\hat{\boldsymbol{\beta}}_T^{(0)}\|_1+\kappa_T\}, \tag{A.25}$$

with

$$\hat{\boldsymbol{\beta}}_T^{(0)} = \underset{\boldsymbol{\beta}\in\mathbb{R}^p}{\operatorname{argmin}} \left\{ \mathcal{L}^{(0)}(\boldsymbol{\beta}) + \lambda_T \|\boldsymbol{\beta}\|_1 \right\}.$$

Since we choose $\lambda_T \geq 2\|\nabla\mathcal{L}^{(0)}(\boldsymbol{\beta}_*^{(0)})\|_\infty$, the first constraint in (A.25) holds. Furthermore, recall that we denote S as the support of $\boldsymbol{\beta}_*^{(0)}$. By Corollary 1 in Negahban et al. (2012) with $\mathcal{M}(S) := \left\{\boldsymbol{\beta}^{(0)} \in \mathbb{R}^p \mid \boldsymbol{\beta}_j^{(0)} = 0 \text{ for all } j \notin S\right\}$, under Assumption 1 and the condition $n_T > 64\left(\tau_0/\alpha_0\right)^2 s\log p$, we have

$$\|\hat{\boldsymbol{\beta}}_T^{(0)} - \boldsymbol{\beta}_*^{(0)}\|_2^2 \leq 18s\lambda_T^2/\alpha_0^2 \text{ and } \|\hat{\boldsymbol{\beta}}_T^{(0)} - \boldsymbol{\beta}_*^{(0)}\|_1 \leq 24s\lambda_T/\alpha_0, \tag{A.26}$$

which together with the choice that $\kappa_T = 24s\lambda_T/\alpha_0$ implies that

$$\|\boldsymbol{\beta}_*^{(0)}\|_1 - \|\hat{\boldsymbol{\beta}}_T^{(0)}\|_1 \leq \|\hat{\boldsymbol{\beta}}_T^{(0)} - \boldsymbol{\beta}_*^{(0)}\|_1 \leq \kappa_T. \tag{A.27}$$

therefore the second constraint in (A.25) is also satisfied. Hence, we have $\boldsymbol{\theta}^{(0)} = \boldsymbol{\beta}_*^{(0)} \in \mathcal{C}_T$, which implies $\boldsymbol{\theta}_*$ is feasible.

To prove the lemma, it remains to show the result in (A.8). We first define the optimal value gap function $F : \mathbb{R}^{(K+1)p} \to \mathbb{R}$ as

$$F(\boldsymbol{\Delta}) = \mathcal{L}\left(\boldsymbol{\theta}_* + \boldsymbol{\Delta}\right) - \mathcal{L}\left(\boldsymbol{\theta}_*\right) + \lambda_0\mathcal{R}\left(\boldsymbol{\theta}_* + \boldsymbol{\Delta}\right) - \lambda_0\mathcal{R}\left(\boldsymbol{\theta}_*\right), \tag{A.28}$$

and $\hat{\boldsymbol{\theta}}$ as the solution to the problem (A.7). We then have $\hat{\boldsymbol{\Delta}} = \hat{\boldsymbol{\theta}} - \boldsymbol{\theta}_* = \underset{\boldsymbol{\Delta}}{\operatorname{argmin}} F(\boldsymbol{\Delta})$ and $F(\mathbf{0}) = 0$. Since both $\hat{\boldsymbol{\theta}}$ and $\boldsymbol{\theta}_*$ are feasible solutions to problem (A.7), we conclude that $F(\hat{\boldsymbol{\Delta}}) \leq 0$.

Since $\mathcal{L}(\cdot)$ is a convex function, by Lemma 2, we can choose λ_0 and λ_1 such that $\lambda_0 \geq 2\left\|\sum_{k=0}^K \frac{n_k}{N}\nabla\mathcal{L}^{(k)}(\boldsymbol{\theta}_*^{(k)})\right\|_\infty$ and $\lambda_1 \geq 2\max_{k\in[K]}\left\|\frac{n_S}{N}\nabla\mathcal{L}^{(k)}(\boldsymbol{\theta}_*^{(k)})\right\|_\infty$ so that

$$\mathcal{L}\left(\boldsymbol{\theta}_* + \hat{\boldsymbol{\Delta}}\right) - \mathcal{L}\left(\boldsymbol{\theta}_*\right) \geq \left\langle \nabla\mathcal{L}\left(\boldsymbol{\theta}_*\right), \hat{\boldsymbol{\Delta}}\right\rangle \geq -\sum_{k=1}^K \frac{\lambda_1}{2}\left\|\hat{\boldsymbol{\Delta}}^{(k)}\right\|_1 - \frac{\lambda_0}{2}\left\|\hat{\boldsymbol{\Delta}}^{(0)}\right\|_1. \tag{A.29}$$

Since the ℓ_1-norm function is decomposable and $\|\boldsymbol{\theta}_{S^c}^{(0)}\|_1 = 0$, by triangle inequality we have

$$\begin{aligned}
&\lambda_0\mathcal{R}\left(\boldsymbol{\theta}_* + \hat{\boldsymbol{\Delta}}\right) - \lambda_0\mathcal{R}\left(\boldsymbol{\theta}_*\right) \\
=&\sum_{k=1}^K \lambda_1\left(\left\|\boldsymbol{\theta}_*^{(k)} + \hat{\boldsymbol{\Delta}}^{(k)}\right\|_1 - \left\|\boldsymbol{\theta}_*^{(k)}\right\|_1\right) + \lambda_0\left(\left\|\boldsymbol{\theta}_*^{(0)} + \hat{\boldsymbol{\Delta}}^{(0)}\right\|_1 - \left\|\boldsymbol{\theta}_*^{(0)}\right\|_1\right) \\
\geq&\sum_{k=1}^K \lambda_1\left(\left\|\hat{\boldsymbol{\Delta}}^{(k)}\right\|_1 - 2\left\|\boldsymbol{\theta}_*^{(k)}\right\|_1\right) + \lambda_0\left(\left\|\hat{\boldsymbol{\Delta}}_{S^c}^{(0)}\right\|_1 - \left\|\hat{\boldsymbol{\Delta}}_S^{(0)}\right\|_1 - 2\left\|\boldsymbol{\theta}_{S^c}^{(0)}\right\|_1\right) \\
\geq&\sum_{k=1}^K \lambda_1\left\|\hat{\boldsymbol{\Delta}}^{(k)}\right\|_1 - 2\sum_{k=1}^K \lambda_1 h + \lambda_0\left(\left\|\hat{\boldsymbol{\Delta}}_{S^c}^{(0)}\right\|_1 - \left\|\hat{\boldsymbol{\Delta}}_S^{(0)}\right\|_1\right).
\end{aligned} \tag{A.30}$$

where we use the property that $\left\|\boldsymbol{\theta}_*^{(k)}\right\|_1 \leq h$ from the parameter space $\Theta(s,h)$.

Combining (A.29) with (A.30) yields

$$0 \geq F(\hat{\boldsymbol{\Delta}}) \geq \sum_{k=1}^{K} \frac{\lambda_1}{2} \left\|\hat{\boldsymbol{\Delta}}^{(k)}\right\|_1 - 2\sum_{k=1}^{K} \lambda_1 h + \frac{\lambda_0}{2} \left(\left\|\hat{\boldsymbol{\Delta}}_{S^c}^{(0)}\right\|_1 - 3\left\|\hat{\boldsymbol{\Delta}}_{S}^{(0)}\right\|_1 \right), \tag{A.31}$$

which leads to the following inequality:

$$\sum_{k=1}^{K} \lambda_1 \left\|\hat{\boldsymbol{\Delta}}^{(k)}\right\|_1 + \lambda_0 \left\|\hat{\boldsymbol{\Delta}}^{(0)}\right\|_1 \leq 4\lambda_0 \left\|\hat{\boldsymbol{\Delta}}_{S}^{(0)}\right\|_1 + 4\sum_{k=1}^{K} \lambda_1 h,$$

as claimed.

B.7 Proof of Lemma 4

Applying the mean value theorem for the loss function $\mathcal{L}$ gives

$$\begin{aligned}
&\mathcal{L}\left(\boldsymbol{\theta}_* + \hat{\boldsymbol{\Delta}}\right) - \mathcal{L}\left(\boldsymbol{\theta}_*\right) - \left\langle \nabla \mathcal{L}\left(\boldsymbol{\theta}_*\right), \hat{\boldsymbol{\Delta}} \right\rangle \\
=&\hat{\boldsymbol{\Delta}}^\top \nabla^2 \mathcal{L}\left(\boldsymbol{\theta}_* + \phi\hat{\boldsymbol{\Delta}}\right) \hat{\boldsymbol{\Delta}} \;(\phi \in (0,1)) \\
=&\sum_{k=1}^{K} \frac{n_S}{N} \left(\hat{\boldsymbol{\Delta}}^{(k)} + \hat{\boldsymbol{\Delta}}^{(0)}\right)^\top \nabla^2 \mathcal{L}^{(k)} \left(\boldsymbol{\theta}^{(k)} + \phi\hat{\boldsymbol{\Delta}}^{(k)}\right) \left(\hat{\boldsymbol{\Delta}}^{(k)} + \hat{\boldsymbol{\Delta}}^{(0)}\right) \\
&+ \frac{n_T}{N} \left(\hat{\boldsymbol{\Delta}}^{(0)}\right)^\top \nabla^2 \mathcal{L}^{(0)}(\boldsymbol{\theta}_*^{(0)} + \phi\hat{\boldsymbol{\Delta}}^{(0)})\hat{\boldsymbol{\Delta}}^{(0)}.
\end{aligned}$$

An application of both RSC and RSM property stated in Assumption 1 leads to

$$\begin{aligned}
&\mathcal{L}\left(\boldsymbol{\theta}_* + \hat{\boldsymbol{\Delta}}\right) - \mathcal{L}\left(\boldsymbol{\theta}_*\right) - \left\langle \nabla \mathcal{L}\left(\boldsymbol{\theta}_*\right), \hat{\boldsymbol{\Delta}} \right\rangle \\
\geq&\sum_{k=1}^{K} \frac{n_S \alpha_k}{N} \left\|\hat{\boldsymbol{\Delta}}^{(k)} + \hat{\boldsymbol{\Delta}}^{(0)}\right\|_2^2 + \frac{n_T \alpha_0}{N} \left\|\hat{\boldsymbol{\Delta}}^{(0)}\right\|_2^2 - R_1(\hat{\boldsymbol{\Delta}}) \\
\geq&\sum_{k=1}^{K} \frac{n_S \alpha_k}{N} \cdot \frac{1}{\gamma_0} \left(\hat{\boldsymbol{\Delta}}^{(k)} + \hat{\boldsymbol{\Delta}}^{(0)}\right)^\top \nabla^2 \mathcal{L}^{(0)}(\boldsymbol{\theta}_*^{(0)} + \phi\hat{\boldsymbol{\Delta}}^{(0)}) \left(\hat{\boldsymbol{\Delta}}^{(k)} + \hat{\boldsymbol{\Delta}}^{(0)}\right) - R_1(\hat{\boldsymbol{\Delta}}) \\
&+ \frac{n_T \alpha_0}{N} \left\|\hat{\boldsymbol{\Delta}}^{(0)}\right\|_2^2 - R_2(\hat{\boldsymbol{\Delta}}),
\end{aligned} \tag{A.32}$$

where

$$\begin{aligned}
R_1(\hat{\boldsymbol{\Delta}}) &:= \sum_{k=1}^{K} \frac{\beta_k \log p}{N} \left\|\hat{\boldsymbol{\Delta}}^{(k)} + \hat{\boldsymbol{\Delta}}^{(0)}\right\|_1^2 + \frac{\beta_0 \log p}{N} \left\|\hat{\boldsymbol{\Delta}}^{(0)}\right\|_1^2 \\
R_2(\hat{\boldsymbol{\Delta}}) &:= \sum_{k=1}^{K} \frac{n_S \alpha_k}{N} \frac{\tau_0}{\gamma_0} \frac{\log p}{n_T} \left\|\hat{\boldsymbol{\Delta}}^{(k)} + \hat{\boldsymbol{\Delta}}^{(0)}\right\|_1^2.
\end{aligned} \tag{A.33}$$

In addition, noting that $\hat{\boldsymbol{\theta}}^{(0)}$ satisfies the constraint outlined in problem (A.7). Again by the mean value theorem and the assumption that $\lambda_T \geq 2\|\mathcal{L}^{(0)}(\boldsymbol{\theta}_*^{(0)})\|_\infty$, we have

$$\begin{aligned}
\left\|\nabla^2\mathcal{L}^{(0)}(\boldsymbol{\theta}_*^{(0)} + \phi\hat{\boldsymbol{\Delta}}^{(0)})\hat{\boldsymbol{\Delta}}^{(0)}\right\|_\infty &= \left\|\nabla\mathcal{L}^{(0)}(\hat{\boldsymbol{\theta}}^{(0)}) - \nabla\mathcal{L}^{(0)}(\boldsymbol{\theta}_*^{(0)})\right\|_\infty \\
&\leq \left\|\nabla\mathcal{L}^{(0)}(\hat{\boldsymbol{\theta}}^{(0)})\right\|_\infty + \left\|\nabla\mathcal{L}^{(0)}(\boldsymbol{\theta}_*^{(0)})\right\|_\infty \\
&\leq 2\lambda_T.
\end{aligned}$$

Hence, a direct application of the Hölder's inequality leads to

$$\left(\hat{\boldsymbol{\Delta}}^{(k)}\right)^\top \nabla^2\mathcal{L}^{(0)}(\boldsymbol{\theta}_*^{(0)} + \phi\hat{\boldsymbol{\Delta}}^{(0)})\hat{\boldsymbol{\Delta}}^{(0)} \leq 2\lambda_T \left\|\hat{\boldsymbol{\Delta}}^{(k)}\right\|_1. \tag{A.34}$$

Recall that we define $n_k = n_S$ for $k = 1, \ldots, K$ and $n_k = n_T$ for $k = 0$. Combining (A.34) with (A.32) and applying the RSC property again, we have

$$\begin{aligned}
&\mathcal{L}\left(\boldsymbol{\theta}_* + \hat{\boldsymbol{\Delta}}\right) - \mathcal{L}\left(\boldsymbol{\theta}_*\right) - \left\langle \nabla\mathcal{L}\left(\boldsymbol{\theta}_*\right), \hat{\boldsymbol{\Delta}}\right\rangle \\
\geq &\sum_{k=1}^{K} \frac{n_S\alpha_k}{N} \cdot \frac{1}{\gamma_0}\left[\alpha_0\left\|\hat{\boldsymbol{\Delta}}^{(k)}\right\|_2^2 + \alpha_0\left\|\hat{\boldsymbol{\Delta}}^{(0)}\right\|_2^2 - 2\lambda_T\left\|\hat{\boldsymbol{\Delta}}^{(k)}\right\|_1\right] \\
&+ \frac{n_T\alpha_0}{N}\left\|\hat{\boldsymbol{\Delta}}^{(0)}\right\|_2^2 - R_1(\hat{\boldsymbol{\Delta}}) - R_2(\hat{\boldsymbol{\Delta}}) - R_3(\hat{\boldsymbol{\Delta}}) \\
\geq &\frac{\alpha_{\min}^2}{\gamma_0}\left[\sum_{k=0}^{K}\frac{n_k}{N}\left\|\hat{\boldsymbol{\Delta}}^{(0)}\right\|_2^2 + \sum_{k=1}^{K}\frac{n_S}{N}\left\|\hat{\boldsymbol{\Delta}}^{(k)}\right\|_2^2\right] - \frac{2\alpha_{\max}}{\gamma_0}\sum_{k=1}^{K}\frac{n_S}{N}\lambda_T\left\|\hat{\boldsymbol{\Delta}}^{(k)}\right\|_1 - \sum_{t=1}^{3} R_t(\hat{\boldsymbol{\Delta}}),
\end{aligned}$$

where R_1, R_2 are defined in (A.33) and

$$R_3(\hat{\boldsymbol{\Delta}}) := \sum_{k=1}^{K}\frac{n_S\alpha_k}{N}\frac{\beta_0}{\gamma_0}\frac{\log p}{n_T}\left(\left\|\hat{\boldsymbol{\Delta}}^{(k)}\right\|_1^2 + \left\|\hat{\boldsymbol{\Delta}}^{(0)}\right\|_1^2\right).$$

Notice that

$$\begin{aligned}
\sum_{t=1}^{3} R_t(\hat{\boldsymbol{\Delta}}) = &\sum_{k=1}^{K} \frac{\beta_k \log p}{N} \left\|\hat{\boldsymbol{\Delta}}^{(k)} + \hat{\boldsymbol{\Delta}}^{(0)}\right\|_1^2 + \frac{\beta_0 \log p}{N} \left\|\hat{\boldsymbol{\Delta}}^{(0)}\right\|_1^2 + \sum_{k=1}^{K} \frac{n_S \alpha_k}{N} \frac{\tau_0}{\gamma_0} \frac{\log p}{n_T} \left\|\hat{\boldsymbol{\Delta}}^{(k)} + \hat{\boldsymbol{\Delta}}^{(0)}\right\|_1^2 \\
&+ \sum_{k=1}^{K} \frac{n_S \alpha_k}{N} \frac{\beta_0}{\gamma_0} \frac{\log p}{n_T} \left(\left\|\hat{\boldsymbol{\Delta}}^{(k)}\right\|_1^2 + \left\|\hat{\boldsymbol{\Delta}}^{(0)}\right\|_1^2 \right) \\
\leq &\left(\frac{\beta_0 \log p}{N} + 2\sum_{k=1}^{K} \frac{\beta_k \log p}{N} + 2\sum_{k=1}^{K} \frac{n_S \alpha_k}{N} \frac{\tau_0}{\gamma_0} \frac{\log p}{n_T} \right) \left\|\hat{\boldsymbol{\Delta}}^{(0)}\right\|_1^2 \\
&+ \sum_{k=1}^{K} \left(\frac{2\beta_k \log p}{N} + \frac{2 n_S \alpha_k}{N} \frac{\tau_0}{\gamma_0} \frac{\log p}{n_T} \right) \left\|\hat{\boldsymbol{\Delta}}^{(k)}\right\|_1^2 \\
\leq &\left(2\sum_{k=0}^{K} \frac{\beta_k \log p}{N} + 2\sum_{k=1}^{K} \frac{n_S \alpha_k}{N} \frac{\tau_0}{\gamma_0} \frac{\log p}{n_T} \right) \left\|\hat{\boldsymbol{\Delta}}^{(0)}\right\|_1^2 \\
&+ \sum_{k=1}^{K} \left(\frac{2\beta_k \log p}{N} + \frac{2 n_S \alpha_k}{N} \frac{\tau_0}{\gamma_0} \frac{\log p}{n_T} \right) \left\|\hat{\boldsymbol{\Delta}}^{(k)}\right\|_1^2 \\
\leq &\frac{2(\alpha_{\max}\tau_0 + \beta_{\max}\gamma_0)}{\gamma_0} \left[\left(\frac{\log p}{N} + \sum_{k=1}^{K} \frac{n_S}{n_T} \frac{\log p}{N} \right) \left\|\hat{\boldsymbol{\Delta}}^{(0)}\right\|_1^2 + \sum_{k=1}^{K} \frac{n_S}{n_T} \frac{\log p}{N} \left\|\hat{\boldsymbol{\Delta}}^{(k)}\right\|_1^2 \right].
\end{aligned}$$

Therefore, Lemma 3 together with the triangle inequality yields

$$\begin{aligned}
&\sum_{t=1}^{3} R_t(\widehat{\boldsymbol{\Delta}}) \cdot \left(\frac{2(\alpha_{\max}\tau_0 + \beta_{\max}\gamma_0)}{\gamma_0} \right)^{-1} \\
\leq &\sum_{k=1}^{K} \frac{n_S}{n_T} \frac{\log p}{N} \left\|\hat{\boldsymbol{\Delta}}^{(k)}\right\|_1^2 + \left(1 + \sum_{k=1}^{K} \frac{n_S}{n_T} \right) \frac{\log p}{N} \left\|\hat{\boldsymbol{\Delta}}^{(0)}\right\|_1^2 \\
= &\frac{n_S}{n_T} \frac{\log p}{N} \sum_{k=1}^{K} \frac{\lambda_1^2}{\lambda_1^2} \left\|\hat{\boldsymbol{\Delta}}^{(k)}\right\|_1^2 + \frac{\log p}{N} \frac{1}{n_T} \left(n_T + \sum_{k=1}^{K} n_S \right) \left\|\hat{\boldsymbol{\Delta}}^{(0)}\right\|_1^2 \\
= &\frac{n_S}{n_T} \frac{\log p}{N} \left(\sum_{k=1}^{K} \frac{\lambda_1^2}{\lambda_1^2} \left\|\hat{\boldsymbol{\Delta}}^{(k)}\right\|_1^2 + \frac{\lambda_0^2}{\lambda_0^2/(N/n_S)} \left\|\hat{\boldsymbol{\Delta}}^{(0)}\right\|_1^2 \right) \\
\leq &\frac{n_S}{n_T} \frac{\log p}{N} \cdot \frac{1}{\lambda_1^2 \wedge [(n_S/N)\lambda_0^2]} \left(\sum_{k=1}^{K} \lambda_1 \left\|\hat{\boldsymbol{\Delta}}^{(k)}\right\|_1 + \lambda_0 \left\|\hat{\boldsymbol{\Delta}}^{(0)}\right\|_1 \right)^2 \\
\leq &\frac{n_S}{n_T} \frac{\log p}{N} \cdot \frac{1}{\lambda_1^2 \wedge [(n_S/N)\lambda_0^2]} \left[32\lambda_0^2 s \left\|\hat{\boldsymbol{\Delta}}^{(0)}\right\|_2^2 + 32 \left(\sum_{k=1}^{K} \lambda_1 h \right)^2 \right],
\end{aligned}$$

Recall that we introduce the shorthand

$$u_n = \frac{64(\alpha_{\max}\tau_0 + \beta_{\max}\gamma_0)}{\gamma_0}\frac{n_S}{n_T}\frac{\log p}{N} \cdot \frac{\lambda_0^2 s}{\lambda_1^2 \wedge [(n_S/N)\lambda_0^2]}$$
$$v_n = \frac{64(\alpha_{\max}\tau_0 + \beta_{\max}\gamma_0)}{\gamma_0}\frac{n_S}{n_T}\frac{\log p}{N} \cdot \frac{\sum_{k=1}^K \lambda_1 h}{\lambda_1^2 \wedge [(n_S/N)\lambda_0^2]}.$$

Therefore, by collecting all the pieces together, we have

$$\mathcal{L}\left(\boldsymbol{\theta}_* + \hat{\boldsymbol{\Delta}}\right) - \mathcal{L}\left(\boldsymbol{\theta}_*\right) - \left\langle \nabla\mathcal{L}\left(\boldsymbol{\theta}_*\right), \hat{\boldsymbol{\Delta}}\right\rangle$$
$$\geq \left(\frac{\alpha_{\min}^2}{\gamma_0} - u_n\right)\left\|\hat{\boldsymbol{\Delta}}^{(0)}\right\|_2^2 + \frac{\alpha_{\min}^2}{\gamma_0}\sum_{k=1}^K \frac{n_k}{N}\left\|\hat{\boldsymbol{\Delta}}^{(k)}\right\|_2^2 - \frac{2\alpha_{\max}}{\gamma_0}\sum_{k=1}^K \frac{n_k}{N}\lambda_T\left\|\hat{\boldsymbol{\Delta}}^{(k)}\right\|_1 - v_n\sum_{k=1}^K \lambda_1 h,$$

which finishes the proof.

B.8 Proof of Lemma 5

Recall that we have the decomposition

$$\tilde{\boldsymbol{\beta}}^{(k)} - \boldsymbol{\beta}_*^{(k)} = -\hat{\boldsymbol{\Theta}}^{(k)}\nabla\mathcal{L}^{(k)}(\boldsymbol{\beta}_*^{(k)}) + \underbrace{\left(\boldsymbol{I} - \hat{\boldsymbol{\Theta}}^{(k)}\nabla^2\mathcal{L}^{(k)}(\hat{\boldsymbol{\beta}}_{\text{Int}}^{(k)})\right)\left(\hat{\boldsymbol{\beta}}_L^{(k)} - \boldsymbol{\beta}_*^{(k)}\right)}_{\boldsymbol{b}^{(k)}}.$$

where $\hat{\boldsymbol{\beta}}_{\text{Int}}^{(k)}$ is a vector intermediating $\hat{\boldsymbol{\beta}}_L^{(k)}$ and $\boldsymbol{\beta}_*^{(k)}$. In the following we define $\boldsymbol{\Sigma}_{\boldsymbol{\beta}}^{(k)} := \mathbb{E}\left[(\mathbf{X}_{\boldsymbol{\beta}}^{(k)})^\top(\mathbf{X}_{\boldsymbol{\beta}}^{(k)})/n_S\right]$, and $\boldsymbol{\Theta}^{(k)} = (\boldsymbol{\Sigma}_{\boldsymbol{\beta}}^{(k)})^{-1}$.

The proof of the lemma relies on two additional lemmas. Recall that $\left\|\boldsymbol{\beta}_*^{(0)}\right\|_0 = s$ and $\left\|\boldsymbol{\beta}_*^{(0)} - \boldsymbol{\beta}_*^{(k)}\right\|_1 \leq h$. The following lemma is a direct consequence of Theorem 1 and Corollary 1 in Negahban et al. (2012).

Lemma 7 *Under the condition of Lemma 5, we have*

$$\left\|\hat{\boldsymbol{\beta}}_L^{(k)} - \boldsymbol{\beta}_*^{(k)}\right\|_1 \lesssim s\sqrt{\frac{\log p}{n_S}} + h, \ \left\|\hat{\boldsymbol{\beta}}_L^{(k)} - \boldsymbol{\beta}_*^{(k)}\right\|_2^2 \lesssim s\frac{\log p}{n_S} + h\sqrt{\frac{\log p}{n_S}}.$$

with probability at least $1 - c_1\exp(-c_2\log p)$.

The next lemma comes from an application of Theorem 3.2 in van de Geer et al. (2014), with condition (D5) in van de Geer et al. (2014) replaced with the result in Lemma 7.

Lemma 8 *Under Assumption 3 and the condition of Lemma 5. Define* $\tau_j^2 := \boldsymbol{\Theta}_{jj}^{(k)}$*. For* $\hat{\tau}$ *and* $\hat{\boldsymbol{\Theta}}^{(k)}$ *obtained from solving problem (A.5), we have*

$$|\hat{\tau}_j^2 - \tau_j^2| \lesssim \left(\frac{s_j\log p}{n_S}\right)^{1/2} \vee \left(\frac{s\log p}{n_S} + h\left(\frac{\log p}{n_S}\right)^{1/2}\right)^{1/2}$$

and $\left|\hat{\boldsymbol{\Theta}}_j^{(k)}\boldsymbol{\Sigma}_{\boldsymbol{\beta}}^{(k)}\left(\hat{\boldsymbol{\Theta}}_j^{(k)}\right)^\top - \tau_j^2\right| \quad \lesssim P_{1n} \wedge P_{2n} + \left|\hat{\tau}_j^2 - \tau_j^2\right|$, *where*

$$P_{1n} = \left\|\boldsymbol{\Sigma}_{\boldsymbol{\beta}}^{(k)}\right\|_\infty \left(\frac{(s_j^2 \vee s^2)\log p}{n_S} + h^2\right), P_{2n} = \Lambda_{\max}^2\left(\frac{(s_j \vee s)\log p}{n_S} + h\left(\frac{\log p}{n_S}\right)^{1/2}\right),$$

with $\Lambda_{\max}^2$ *being the largest eigenvalue of* $\boldsymbol{\Sigma}_{\boldsymbol{\beta}}^{(k)}$.

We start with the first term, $\hat{\boldsymbol{\Theta}}^{(k)}\nabla\mathcal{L}^{(k)}(\boldsymbol{\beta}_*^{(k)})$. Let $\left(\boldsymbol{a}_j^{(k)}\right)^\top := \boldsymbol{e}_j^\top\hat{\boldsymbol{\Theta}}^{(k)}$. Plugging in the definition of $\mathcal{L}^{(k)}(\boldsymbol{\beta}_*^{(k)})$ and applying Assumption 2, we have for some universal constant $c > 0$,

$$P\left(\max_{1\le j\le p}\left|\frac{1}{2n_S}\sum_{i=1}^{n_S}\left(\boldsymbol{a}_j^{(k)}\right)^\top\nabla\ell^{(k)}(\boldsymbol{\beta}_*^{(k)}; z_i^{(k)})\right| > t\;\middle|\;\hat{\boldsymbol{\Theta}}^{(k)}\right) \le p\exp\left(-\frac{cn_S^2t^2}{\sigma^2 c_\Omega}\right)$$

where

$$c_\Omega := \max_{1\le j\le p}\left(\hat{\boldsymbol{\Theta}}^{(k)}\mathbb{E}\left(\nabla\mathcal{L}^{(k)}(\boldsymbol{\beta}_*^{(k)})[\nabla\mathcal{L}^{(k)}(\boldsymbol{\beta}_*^{(k)})]^\top\right)\left(\hat{\boldsymbol{\Theta}}^{(k)}\right)^\top\right)_{j,j} = \max_{1\le j\le p}\left(\hat{\boldsymbol{\Theta}}^{(k)}\boldsymbol{\Sigma}_{\boldsymbol{\beta}}^{(k)}\left(\hat{\boldsymbol{\Theta}}^{(k)}\right)^\top\right)_{j,j}.$$

where recall we define $\boldsymbol{\Sigma}_{\boldsymbol{\beta}}^{(k)} := \mathbb{E}\left[(\mathbf{X}_{\boldsymbol{\beta}}^{(k)})^\top(\mathbf{X}_{\boldsymbol{\beta}}^{(k)})/n_S\right]$. Therefore, to prove (A.18), it suffices to bound $c_\Omega/(n_S^2t^2)$ with $t = \sqrt{\frac{\log p}{n_S}} + h\sqrt{\frac{\log p}{n_S}}$. According to Lemma 8 and the conditions outlined in Theorem 2, we have $P_{1n} = O(h^2) + o(1)$ and $P_{2n} = o(1)$ and thus $c_\Omega = \max_{1\le j\le p}\tau_j^2 + O(h^2) + o(1)$. By Assumption 3, $\max_{1\le j\le p}\tau_j^2 = \max_{1\le j\le p}\boldsymbol{\Theta}_{jj}^{(k)}$ is bounded above. Therefore (A.18) holds.

Next, we aim at the second term, $\boldsymbol{\beta}^{(k)}$. Notice that with a slight abuse of notation, we have $\nabla^2\ell(z_i^{(k)};\boldsymbol{\beta}) = \mathbf{X}_i^{(k)}\nabla^2\rho(\boldsymbol{\beta})(\mathbf{X}_i^{(k)})^\top$. Applying Hölder's inequality and triangle inequality, we obtain

$$\begin{aligned}
\left\|\boldsymbol{\beta}^{(k)}\right\|_\infty &= \left\|\left(\boldsymbol{I} - \hat{\boldsymbol{\Theta}}^{(k)}\nabla^2\mathcal{L}^{(k)}(\hat{\boldsymbol{\beta}}_{\mathrm{Int}}^{(k)})\right)\left(\hat{\boldsymbol{\beta}}_L^{(k)} - \boldsymbol{\beta}_*^{(k)}\right)\right\|_\infty \\
&\le \left\|\left(\boldsymbol{I} - \hat{\boldsymbol{\Theta}}^{(k)}\nabla^2\mathcal{L}^{(k)}(\hat{\boldsymbol{\beta}}_L^{(k)})\right)\left(\hat{\boldsymbol{\beta}}_L^{(k)} - \boldsymbol{\beta}_*^{(k)}\right)\right\|_\infty \\
&\quad + \left\|\hat{\boldsymbol{\Theta}}^{(k)}\left(\nabla^2\mathcal{L}^{(k)}(\hat{\boldsymbol{\beta}}_L^{(k)}) - \nabla^2\mathcal{L}^{(k)}(\hat{\boldsymbol{\beta}}_{\mathrm{Int}}^{(k)})\right)\left(\hat{\boldsymbol{\beta}}_L^{(k)} - \boldsymbol{\beta}_*^{(k)}\right)\right\|_\infty \\
&\le \underbrace{\left\|\hat{\boldsymbol{\beta}}_L^{(k)} - \boldsymbol{\beta}_*^{(k)}\right\|_1}_{E_1}\underbrace{\max_{1\le j\le p}\left\|\hat{\boldsymbol{\Theta}}_j^{(k)}\nabla^2\mathcal{L}^{(k)}(\hat{\boldsymbol{\beta}}_L^{(k)}) - \boldsymbol{e}_j^\top\right\|_\infty}_{E_2} \\
&\quad + \underbrace{\frac{1}{n_S}\sum_{i=1}^{n_S}\left|\left(\nabla^2\rho(\hat{\boldsymbol{\beta}}_L^{(k)}) - \nabla^2\rho(\hat{\boldsymbol{\beta}}_{\mathrm{Int}}^{(k)})\right)(\mathbf{X}_i^{(k)})^\top\left(\hat{\boldsymbol{\beta}}_L^{(k)} - \boldsymbol{\beta}_*^{(k)}\right)\right|}_{E_3}\underbrace{\left\|\hat{\boldsymbol{\Theta}}^{(k)}\mathbf{X}_i^{(k)}\right\|_\infty}_{E_4}.
\end{aligned}$$

where recall that $\hat{\boldsymbol{\Theta}}_j^{(k)}$ denotes the jth row of $\hat{\boldsymbol{\Theta}}^{(k)}$. We then proceed term by term.

For E_1, another application of Lemma 7 yields $E_1 \lesssim s\sqrt{\log p/n_S} + h$. As for E_2, from the optimality condition of nodewise regression problem (A.5), we have $E_2 \le \tilde{\lambda}_{kj}/\hat{\tau}_j{}^2$. For details, one may check the derivation of (10) in van de Geer et al. (2014). From Lemma 8, we have $\hat{\tau}_j = \tau_j + o(1)$. By Assumption 3, $\tau_j = \mathbf{\Theta}_{jj}^{(k)}$ is bounded below. Therefore as we choose $\tilde{\lambda}_{kj} \asymp \sqrt{\log p/n_S}$, we have $E_2 \asymp \sqrt{\log p/n_S}$. As for E_3, according to the Lipschitz condition on $\nabla^2\rho(\cdot)$, we have

$$\begin{aligned}
E_3 =& \frac{1}{n_S}\sum_{i=1}^{n_S}\left|\left(\nabla^2\rho(\hat{\boldsymbol{\beta}}_L^{(k)}) - \nabla^2\rho(\hat{\boldsymbol{\beta}}_{\text{Int}}^{(k)})\right)(\mathbf{X}_i^{(k)})^\top\left(\hat{\boldsymbol{\beta}}_L^{(k)} - \boldsymbol{\beta}_*^{(k)}\right)\right| \\
\le& \frac{1}{n_S}\sum_{i=1}^{n_S}\left|(\mathbf{X}_i^{(k)})^\top\hat{\boldsymbol{\beta}}_L^{(k)} - (\mathbf{X}_i^{(k)})^\top\hat{\boldsymbol{\beta}}_{\text{Int}}^{(k)}\right|\left|(\mathbf{X}_i^{(k)})^\top\left(\hat{\boldsymbol{\beta}}_L^{(k)} - \boldsymbol{\beta}_*^{(k)}\right)\right| \\
\le& \frac{1}{n_S}\sum_{i=1}^{n_S}\left|(\mathbf{X}_i^{(k)})^\top\left(\hat{\boldsymbol{\beta}}_L^{(k)} - \boldsymbol{\beta}_*^{(k)}\right)\right|^2 = \left\|\mathbf{X}^{(k)}\left(\hat{\boldsymbol{\beta}}_L^{(k)} - \boldsymbol{\beta}_*^{(k)}\right)\right\|_2^2
\end{aligned}$$

With condition (D1) in Assumption 3, an combination of Lemma 13 in Loh and Wainwright (2011) and Lemma 7 yields

$$\left\|\mathbf{X}^{(k)}\left(\hat{\boldsymbol{\beta}}_L^{(k)} - \boldsymbol{\beta}_*^{(k)}\right)\right\|_2^2 \lesssim \left\|\hat{\boldsymbol{\beta}}_L^{(k)} - \boldsymbol{\beta}_*^{(k)}\right\|_2^2 + \frac{\log p}{n_S}\left\|\hat{\boldsymbol{\beta}}_L^{(k)} - \boldsymbol{\beta}_*^{(k)}\right\|_1^2 \lesssim s\frac{\log p}{n_S} + h\sqrt{\frac{\log p}{n_S}}.$$

where the last inequality is based on the assumption that $s\log p/n_S = o(1)$ and $h\sqrt{\log p/n_S} = o(1)$. Therefore, we have $E_3 \lesssim s\log p/n_S + h\sqrt{\log p/n_S}$.

The analysis of E_4 follows similar arguments to those presented in the proof of Theorem 21 in Lee et al. (2017); for completeness, we restate the key steps here. By applying Lemma 8, we have:

$$\begin{aligned}
\left\|\hat{\mathbf{\Theta}}^{(k)}\mathbf{X}_i^{(k)}\right\|_\infty &\le \max_{1\le j\le p}\left\|\hat{\mathbf{\Theta}}_j^{(k)}(\mathbf{X}^{(k)})^\top\right\|_\infty \lesssim \max_{1\le j\le p}\left\|\hat{\mathbf{\Theta}}_j^{(k)}(\mathbf{X}_{\boldsymbol{\beta}_*^{(k)}})^\top\right\|_\infty \\
&\le \max_{1\le j\le p}\frac{1}{\hat{\tau}_j^2}\left\|\mathbf{X}_{\boldsymbol{\beta}_*^{(k)},j} - \mathbf{X}_{\boldsymbol{\beta}_*^{(k)},-j}\hat{\boldsymbol{\gamma}}_j\right\|_\infty \\
&\lesssim \max_{1\le j\le p}\frac{1}{\tau_j^2}\left\|\mathbf{X}_{\boldsymbol{\beta}_*^{(k)},j} - \mathbf{X}_{\boldsymbol{\beta}_*^{(k)},-j}\hat{\boldsymbol{\gamma}}_j\right\|_\infty \\
&\lesssim \max_{1\le j\le p}\left[\frac{1}{\tau_j^2}\left\|\mathbf{X}_{\boldsymbol{\beta}_*^{(k)},j} - \mathbf{X}_{\boldsymbol{\beta}_*^{(k)},-j}\boldsymbol{\gamma}_j\right\|_\infty + \frac{1}{\tau_j^2}\left\|\mathbf{X}_{\boldsymbol{\beta}_*^{(k)},-j}\right\|_\infty\left\|\hat{\boldsymbol{\gamma}}_j - \boldsymbol{\gamma}_j\right\|_1\right] \\
&\lesssim 1 + \frac{\max_{1\le j\le p}s_j\log p}{n_S}.
\end{aligned}$$

where for the last inequality we use condition (D1) in Assumption 3. Therefore, since $\max_{1\le j\le p}s_j\log p/n_S = o(1)$, we have E_4 bounded above. Integrating the above arguments leads to $\|\boldsymbol{\beta}^{(k)}\|_\infty \lesssim \frac{s\log p}{n_S} + h\sqrt{\frac{\log p}{n_S}}$ with probability larger than $1 - c_1\exp(-c_2\log p)$. This finishes the proof.

Proof of Lemma 6 Notice that $\nabla\tilde{\mathcal{L}}^{(k)}(\boldsymbol{\beta}) = \boldsymbol{\beta} - \tilde{\boldsymbol{\beta}}^{(k)}$. Therefore, following the proof of Lemma 2, by applying Hölder's inequality, we obtain

$$\begin{aligned}
|\langle \nabla\mathcal{L}(\boldsymbol{\theta}_*), \boldsymbol{\Delta}\rangle| &= \sum_{k=1}^{K}\left|\left\langle \frac{n_S}{N}(\boldsymbol{\beta}_*^{(k)} - \tilde{\boldsymbol{\beta}}^{(k)}), \boldsymbol{\Delta}^{(k)}\right\rangle\right| + \left|\left\langle \sum_{k=1}^{K}\frac{n_S}{N}(\boldsymbol{\beta}_*^{(k)} - \tilde{\boldsymbol{\beta}}^{(k)}) + \frac{n_T}{N}\nabla\mathcal{L}^{(0)}(\boldsymbol{\beta}_*^{(0)}), \boldsymbol{\Delta}^{(0)}\right\rangle\right| \\
&\le \sum_{k=1}^{K}\left\|\frac{n_S}{N}(\boldsymbol{\beta}_*^{(k)} - \tilde{\boldsymbol{\beta}}^{(k)})\right\|_\infty \left\|\boldsymbol{\Delta}^{(k)}\right\|_1 + \left\|\sum_{k=1}^{K}\frac{n_S}{N}(\boldsymbol{\beta}_*^{(k)} - \tilde{\boldsymbol{\beta}}^{(k)}) + \frac{n_T}{N}\nabla\mathcal{L}^{(0)}(\boldsymbol{\beta}_*^{(0)})\right\|_\infty \left\|\boldsymbol{\Delta}^{(0)}\right\|_1.
\end{aligned}$$

Notice that we have

$$\tilde{\boldsymbol{\beta}}^{(k)} - \boldsymbol{\beta}_*^{(k)} = -\hat{\boldsymbol{\Theta}}^{(k)}\nabla\mathcal{L}^{(k)}(\boldsymbol{\beta}_*^{(k)}) + \underbrace{\left(\boldsymbol{I} - \hat{\boldsymbol{\Theta}}^{(k)}\nabla^2\mathcal{L}^{(k)}(\hat{\boldsymbol{\beta}}_{\text{Int}}^{(k)})\right)\left(\hat{\boldsymbol{\beta}}_L^{(k)} - \boldsymbol{\beta}_*^{(k)}\right)}_{\boldsymbol{b}^{(k)}}. \quad \text{(A.35)}$$

In Lemma 5 we have shown that with probability larger than $1 - c_1\exp(-c_2\log p)$,

$$\left\|\hat{\boldsymbol{\Theta}}^{(k)}\nabla\mathcal{L}^{(k)}(\boldsymbol{\beta}_*^{(k)})\right\|_\infty \lesssim \sqrt{\frac{\log p}{n_S}} + h\sqrt{\frac{\log p}{n_S}} \quad \text{and} \quad \left\|\boldsymbol{\beta}^{(k)}\right\|_\infty \lesssim \frac{s\log p}{n_S} + h\sqrt{\frac{\log p}{n_S}}. \quad \text{(A.36)}$$

Thus in order to guarantee

$$\lambda_1 \ge \left\|-\frac{n_S}{N}\hat{\boldsymbol{\Theta}}^{(k)}\nabla\mathcal{L}^{(k)}(\boldsymbol{\beta}_*^{(k)}) + \frac{n_S}{N}\boldsymbol{\beta}^{(k)}\right\|_\infty,$$

it suffices to choose $\lambda_1 = c_k\left(\sqrt{\frac{n_S}{N}\frac{\log p}{N}} + \frac{s\log p}{N} + \frac{n_S}{N}\sqrt{\frac{\log p}{n_S}}h\right)$ for some sufficiently large constant c_k.

Next, we shift our focus to the second term. Similar to the arguments in Lemma 2 and Lemma 5 with $\left(\boldsymbol{a}_j^{(k)}\right)^\top := \boldsymbol{e}_j^\top\hat{\boldsymbol{\Theta}}^{(k)}$ for $k = 1, \ldots, K$ and $\left(\boldsymbol{a}_j^{(0)}\right)^\top := \boldsymbol{e}_j^\top$, we have

$$P\left(\max_{1\le j\le p}\left|\frac{1}{2N}\sum_{k=0}^{K}\sum_{i=1}^{n_k}\left(\boldsymbol{a}_j^{(k)}\right)^\top\nabla\ell^{(k)}(\boldsymbol{\beta}_*^{(k)}; z_i^{(k)})\right| > t\ \middle|\ \{\hat{\boldsymbol{\Theta}}^{(k)}\}_{k=1}^K\right) \le p\exp\left(-\frac{cN^2t^2}{c_{\Omega'}}\right).$$

With $t = \sqrt{\frac{\log p}{N}} + h\sqrt{\frac{\log p}{N}}$, we similarly have $c_{\Omega'}/(N^2t^2)$ bounded above with probability larger than $1 - c_1\exp(-c_2\log p)$. This result together with (A.36) indicates that

$$\left\|\sum_{k=1}^{K}\frac{n_S}{N}(\boldsymbol{\beta}_*^{(k)} - \tilde{\boldsymbol{\beta}}^{(k)}) + \frac{n_T}{N}\nabla\mathcal{L}^{(0)}(\boldsymbol{\beta}_*^{(0)})\right\|_\infty \lesssim \sqrt{\frac{\log p}{N}} + \frac{Ks\log p}{N} + \sum_{k=1}^{K}\frac{n_S}{N}\sqrt{\frac{\log p}{n_S}}h,$$

so it suffices to choose $\lambda_0 = c_0\left(\sqrt{\frac{\log p}{N}} + \frac{Ks\log p}{N} + \sum_{k=1}^{K}\frac{n_S}{N}\sqrt{\frac{\log p}{n_S}}h\right)$ for some sufficiently large constant c_0. The proof is then finished.

Appendix C. Additional Simulation and Real Data Analysis

C.1 Scalability with Respect to Dimension p and Number of Source Tasks K

We next examine the scalability of *TransMission* and its distributed variant (*D-TransMission*) with respect to the feature dimension p and the number of source tasks K. Table A.3 reports the prediction and estimation performance across different feature dimensions $p \in \{500, 1000, 1500, 2000\}$, under both homogeneous and heterogeneous settings. Consistent with the observations in the main text, *TransMission* maintains comparable accuracy in homogeneous settings and demonstrates superior robustness under distribution shifts. The results show no significant degradation in performance even when p quadruples from 500 to 2000.

The computational complexity of the proposed joint optimization framework depends on the choice of optimization algorithm. With first-order type methods, the iteration complexity is dimension-independent. Within each iteration, the solution involves performing matrix-vector multiplications, leading to a computational complexity of $O(Kp)$, which indeed increases with both p and K. In *D-TransMission*, if node-wise Lasso is used for constructing the de-biased estimator, the computational cost would increase by a factor p, leading to a more expensive computational cost $O(Kp^2)$. However, in practice, the de-biased estimator can be replaced by other asymptotically unbiased estimators such as the SCAD estimator (Fan and Li, 2001), which reduces the computational complexity of *D-TransMission* back to $O(Kp)$.

We would like to note that in practice, one could first cluster the original datasets into K^* homogeneous groups, then perform *(D-)TransMission* on these $K^* \ll K$ clusters to account for inter-cluster distribution shifts. Such a clustering approach can alleviate the growing computational cost with K of the proposed method, but at the same time benefit from the robustness property.

C.2 Robustness under Heavy-Tailed Noise Distributions

To further evaluate the robustness of *TransMission* against conditional shifts arising from heterogeneous noise distributions, we conduct additional experiments where the target samples retain Gaussian noise while the source samples are drawn from heavy-tailed distributions. Specifically, the source task errors are generated from a Student-t distribution with degrees of freedom $t_{\text{df}} \in \{3, 5\}$, while the target task follows the standard normal noise distribution. Table A.2 summarizes the results across these designs.

We observe that *TransMission* consistently attains the lowest prediction error and outperforms competing baselines even under substantial departures from normality. Interestingly, its performance advantage becomes apparent even under small h (i.e., mildly heterogeneous settings), indicating that the estimator effectively identifies and leverages transferable information early on, rather than requiring strong cross-task similarity. These findings corroborate the theoretical robustness of the proposed fused-regularization framework to heavy-tailed conditional shifts.

Table A.2: Performance comparison across methods under heavy-tailed or highly correlated designs. The column t_{df} denotes the degrees of freedom of the Student-t distribution used in the experiment design. The column ρ controls the correlation strength in the source covariate covariance matrices, where larger ρ corresponds to stronger correlations.

h	ρ	t_{df}	Agg-Lasso	Single-Lasso	Trans-GLM	TransHDGLM	Trans-Lasso	TransMission
5	0.5	3	0.345	0.891	0.384	0.377	0.360	**0.333**
5	0.5	5	0.261	0.899	0.279	0.261	0.273	**0.237**
5	0.6	3	0.400	0.889	0.449	0.445	0.405	**0.370**
5	0.6	5	0.267	0.896	0.288	0.272	0.276	**0.256**
5	0.7	3	0.443	0.897	0.492	0.512	0.442	**0.424**
5	0.7	5	0.321	0.891	0.337	0.328	0.316	**0.302**
5	0.8	3	0.521	0.896	0.548	0.637	0.518	**0.494**
5	0.8	5	0.415	0.893	0.431	0.436	0.416	**0.359**
10	0.5	3	0.514	0.893	0.519	0.517	0.512	**0.415**
10	0.5	5	0.488	0.895	0.439	0.424	0.491	**0.328**
10	0.6	3	0.595	0.894	0.598	0.556	0.584	**0.449**
10	0.6	5	0.461	0.895	0.439	0.402	0.465	**0.337**
10	0.7	3	0.628	0.898	0.620	0.618	0.618	**0.499**
10	0.7	5	0.508	0.897	0.501	0.447	0.500	**0.385**
10	0.8	3	0.676	0.897	0.678	0.704	0.669	**0.554**
10	0.8	5	0.629	0.894	0.609	0.533	0.621	**0.427**
15	0.5	3	0.805	0.894	0.676	0.654	0.772	**0.529**
15	0.5	5	0.856	0.886	0.624	0.617	0.827	**0.416**
15	0.6	3	0.898	0.894	0.778	0.691	0.862	**0.542**
15	0.6	5	0.793	0.895	0.627	0.573	0.772	**0.451**
15	0.7	3	0.896	0.894	0.782	0.742	0.854	**0.606**
15	0.7	5	0.798	0.894	0.694	0.591	0.770	**0.479**
15	0.8	3	0.912	0.893	0.832	0.795	0.899	**0.637**
15	0.8	5	0.889	0.893	0.782	0.645	0.846	**0.514**
20	0.5	3	1.174	0.896	0.861	0.784	1.104	**0.623**
20	0.5	5	1.290	0.894	0.826	0.817	1.235	**0.504**
20	0.6	3	1.263	0.898	0.927	0.815	1.215	**0.617**
20	0.6	5	1.217	0.894	0.820	0.740	1.177	**0.531**
20	0.7	3	1.218	0.895	0.899	0.833	1.122	**0.684**
20	0.7	5	1.154	0.895	0.871	0.730	1.104	**0.580**
20	0.8	3	1.190	0.894	0.955	0.872	1.162	**0.726**
20	0.8	5	1.164	0.893	0.918	0.762	1.081	**0.604**

Table A.3: Performance comparison across methods under different feature dimension p.

h	ρ	p	Agg-Lasso	Single-Lasso	Trans-GLM	TransHDGLM	Trans-Lasso	TransMission
10	0.100	1000	0.349	1.060	0.373	0.257	0.361	**0.236**
10	0.100	1500	0.343	1.158	0.358	0.267	0.362	**0.262**
10	0.100	2000	0.359	1.246	0.386	**0.300**	0.376	0.301
10	0.200	1000	0.378	1.055	0.383	0.288	0.396	**0.247**
10	0.200	1500	0.367	1.147	0.361	0.284	0.370	**0.266**
10	0.200	2000	0.357	1.241	0.360	0.275	0.387	**0.268**
10	0.300	1000	0.399	1.053	0.389	0.312	0.413	**0.266**
10	0.300	1500	0.407	1.142	0.408	0.321	0.405	**0.295**
10	0.300	2000	0.427	1.243	0.425	0.342	0.431	**0.319**
10	0.400	1000	0.409	1.060	0.406	0.310	0.411	**0.265**
10	0.400	1500	0.411	1.156	0.420	0.305	0.427	**0.285**
10	0.400	2000	0.437	1.239	0.434	0.345	0.460	**0.318**
15	0.100	1000	0.672	1.052	0.609	0.475	0.675	**0.308**
15	0.100	1500	0.658	1.149	0.608	0.507	0.674	**0.361**
15	0.100	2000	0.684	1.240	0.670	0.556	0.669	**0.427**
15	0.200	1000	0.764	1.052	0.633	0.526	0.773	**0.328**
15	0.200	1500	0.690	1.150	0.627	0.512	0.688	**0.375**
15	0.200	2000	0.686	1.238	0.630	0.496	0.707	**0.386**
15	0.300	1000	0.773	1.059	0.657	0.556	0.786	**0.363**
15	0.300	1500	0.747	1.154	0.675	0.561	0.750	**0.396**
15	0.300	2000	0.756	1.249	0.714	0.597	0.746	**0.426**
15	0.400	1000	0.764	1.061	0.677	0.542	0.744	**0.365**
15	0.400	1500	0.776	1.158	0.676	0.520	0.762	**0.409**
15	0.400	2000	0.756	1.239	0.715	0.565	0.777	**0.424**
20	0.100	1000	1.072	1.055	0.969	0.716	1.053	**0.381**
20	0.100	1500	1.050	1.150	0.961	0.755	1.032	**0.456**
20	0.100	2000	1.066	1.233	1.007	0.844	1.066	**0.555**
20	0.200	1000	1.274	1.061	0.997	0.786	1.229	**0.410**
20	0.200	1500	1.116	1.152	0.933	0.770	1.088	**0.480**
20	0.200	2000	1.087	1.255	0.991	0.767	1.083	**0.495**
20	0.300	1000	1.213	1.069	0.945	0.794	1.204	**0.455**
20	0.300	1500	1.151	1.154	0.987	0.808	1.116	**0.504**
20	0.300	2000	1.115	1.236	1.028	0.848	1.098	**0.531**
20	0.400	1000	1.180	1.050	0.967	0.784	1.120	**0.459**
20	0.400	1500	1.156	1.149	0.953	0.762	1.138	**0.497**
20	0.400	2000	1.110	1.233	1.000	0.818	1.131	**0.531**

Table A.4: Performance comparison under log-normal dense shift patterns. The parameter σ controls the concentration of shift energy: $\sigma \in \{0.5, 1.0, 1.5\}$ correspond to approximately 60%, 20%, and 6% effective sparsity, respectively. Bold denotes the best-performing method per row.

h	ρ	σ	Agg-Lasso	Single-Lasso	Trans-GLM	TransHDGLM	TransMission
10	0.1	0.5	0.305	0.902	0.293	0.274	**0.250**
10	0.1	1.0	0.307	0.895	0.306	0.251	**0.211**
10	0.1	1.5	0.301	0.896	0.269	0.181	**0.150**
10	0.2	0.5	0.364	0.886	0.314	0.327	**0.265**
10	0.2	1.0	0.353	0.897	0.300	0.285	**0.229**
10	0.2	1.5	0.318	0.895	0.263	0.205	**0.167**
10	0.3	0.5	0.413	0.887	0.320	0.369	**0.285**
10	0.3	1.0	0.411	0.894	0.319	0.347	**0.252**
10	0.3	1.5	0.372	0.887	0.284	0.230	**0.173**
10	0.4	0.5	0.388	0.895	0.328	0.361	**0.280**
10	0.4	1.0	0.395	0.894	0.330	0.319	**0.249**
10	0.4	1.5	0.365	0.893	0.298	0.237	**0.186**
15	0.1	0.5	0.614	0.894	0.482	0.534	**0.421**
15	0.1	1.0	0.610	0.894	0.486	0.478	**0.329**
15	0.1	1.5	0.602	0.892	0.422	0.329	**0.201**
15	0.2	0.5	0.763	0.896	0.525	0.616	**0.436**
15	0.2	1.0	0.750	0.895	0.503	0.522	**0.355**
15	0.2	1.5	0.658	0.894	0.417	0.360	**0.221**
15	0.3	0.5	0.858	0.893	0.547	0.671	**0.466**
15	0.3	1.0	0.839	0.884	0.543	0.596	**0.380**
15	0.3	1.5	0.745	0.881	0.430	0.404	**0.230**
15	0.4	0.5	0.805	0.897	0.541	0.636	**0.458**
15	0.4	1.0	0.806	0.895	0.536	0.564	**0.380**
15	0.4	1.5	0.729	0.892	0.464	0.392	**0.242**
20	0.1	0.5	1.007	0.895	0.692	0.780	**0.645**
20	0.1	1.0	1.032	0.891	0.727	0.702	**0.482**
20	0.1	1.5	1.008	0.892	0.608	0.518	**0.257**
20	0.2	0.5	1.292	0.892	0.806	0.842	**0.661**
20	0.2	1.0	1.293	0.893	0.770	0.756	**0.511**
20	0.2	1.5	1.155	0.892	0.616	0.544	**0.280**
20	0.3	0.5	1.420	0.895	0.809	0.883	**0.664**
20	0.3	1.0	1.409	0.882	0.823	0.816	**0.523**
20	0.3	1.5	1.239	0.885	0.630	0.598	**0.298**
20	0.4	0.5	1.353	0.894	0.765	0.852	**0.644**
20	0.4	1.0	1.357	0.885	0.812	0.786	**0.514**
20	0.4	1.5	1.220	0.892	0.639	0.579	**0.309**

Table A.5: Performance comparison under hierarchical shift structures across different correlation strengths. The column "Structure" denotes the hierarchical configuration used in the shift design.

h	ρ	Structure	Agg-Lasso	Single-Lasso	Trans-GLM	TransHDGLM	TransMission
10	0.1	10_40	9.460	0.912	4.536	1.219	**0.159**
10	0.1	20_80	4.615	0.918	3.560	1.106	**0.181**
10	0.1	5_100	18.921	0.918	5.052	1.293	**0.114**
10	0.2	10_40	11.017	0.911	7.597	1.223	**0.188**
10	0.2	20_80	6.251	0.918	5.406	1.148	**0.218**
10	0.2	5_100	26.453	0.917	8.880	1.260	**0.127**
10	0.3	10_40	11.377	0.915	7.104	1.238	**0.221**
10	0.3	20_80	6.266	0.896	5.553	1.133	**0.262**
10	0.3	5_100	23.759	0.915	9.646	1.247	**0.132**
10	0.4	10_40	9.496	0.915	6.844	1.223	**0.272**
10	0.4	20_80	6.609	0.913	5.575	1.147	**0.286**
10	0.4	5_100	23.982	0.900	10.707	1.268	**0.154**
15	0.1	10_40	19.650	0.915	6.951	1.277	**0.197**
15	0.1	20_80	9.024	0.912	5.721	1.237	**0.212**
15	0.1	5_100	40.037	0.905	7.851	1.288	**0.143**
15	0.2	10_40	22.254	0.921	13.244	1.261	**0.245**
15	0.2	20_80	12.587	0.923	9.513	1.250	**0.264**
15	0.2	5_100	56.353	0.914	15.694	1.285	**0.168**
15	0.3	10_40	23.992	0.920	13.307	1.262	**0.278**
15	0.3	20_80	13.105	0.902	10.546	1.229	**0.293**
15	0.3	5_100	50.864	0.915	18.544	1.269	**0.157**
15	0.4	10_40	20.128	0.924	12.731	1.245	**0.310**
15	0.4	20_80	13.772	0.918	10.589	1.233	**0.354**
15	0.4	5_100	51.419	0.903	20.651	1.290	**0.164**
20	0.1	10_40	33.582	0.900	9.792	1.278	**0.253**
20	0.1	20_80	15.051	0.908	8.408	1.256	**0.256**
20	0.1	5_100	69.614	0.896	11.426	1.323	**0.172**
20	0.2	10_40	38.054	0.915	20.796	1.276	**0.297**
20	0.2	20_80	21.175	0.917	15.093	1.246	**0.319**
20	0.2	5_100	98.803	0.896	23.902	1.267	**0.194**
20	0.3	10_40	40.681	0.921	21.166	1.269	**0.344**
20	0.3	20_80	22.027	0.895	17.008	1.242	**0.338**
20	0.3	5_100	89.358	0.896	30.822	1.286	**0.182**
20	0.4	10_40	35.034	0.920	21.655	1.291	**0.365**
20	0.4	20_80	23.315	0.914	17.549	1.240	**0.402**
20	0.4	5_100	89.898	0.899	31.777	1.285	**0.205**

Table A.6: Performance comparison under correlated shift settings across different correlation strengths. The column "Correlation" denotes the correlation level used in constructing the shift design.

h	ρ	Correlation	Agg-Lasso	Single-Lasso	Trans-GLM	TransHDGLM	TransMission
10	0.1	0.1	0.363	0.895	0.317	0.296	**0.234**
10	0.1	0.3	0.496	0.908	0.494	0.424	**0.282**
10	0.1	0.5	0.620	0.915	0.588	0.500	**0.353**
10	0.2	0.1	0.423	0.893	0.327	0.333	**0.239**
10	0.2	0.3	0.495	0.915	0.477	0.437	**0.277**
10	0.2	0.5	0.616	0.915	0.593	0.544	**0.360**
10	0.3	0.1	0.458	0.895	0.343	0.356	**0.242**
10	0.3	0.3	0.544	0.916	0.552	0.438	**0.301**
10	0.3	0.5	0.626	0.915	0.600	0.533	**0.345**
10	0.4	0.1	0.438	0.895	0.356	0.360	**0.259**
10	0.4	0.3	0.482	0.908	0.478	0.417	**0.281**
10	0.4	0.5	0.602	0.911	0.609	0.507	**0.342**
15	0.1	0.1	0.776	0.895	0.581	0.553	**0.351**
15	0.1	0.3	1.085	0.915	0.934	0.713	**0.443**
15	0.1	0.5	1.467	0.915	1.191	0.803	**0.632**
15	0.2	0.1	0.890	0.893	0.589	0.569	**0.335**
15	0.2	0.3	1.123	0.915	1.024	0.726	**0.450**
15	0.2	0.5	1.466	0.915	1.243	0.794	**0.601**
15	0.3	0.1	0.978	0.894	0.619	0.632	**0.360**
15	0.3	0.3	1.154	0.915	1.096	0.704	**0.453**
15	0.3	0.5	1.373	0.915	1.208	0.797	**0.609**
15	0.4	0.1	0.949	0.893	0.634	0.601	**0.381**
15	0.4	0.3	1.059	0.915	0.998	0.700	**0.443**
15	0.4	0.5	1.347	0.915	1.229	0.808	**0.607**
20	0.1	0.1	1.330	0.895	0.905	0.787	**0.474**
20	0.1	0.3	1.919	0.917	1.521	0.936	**0.608**
20	0.1	0.5	2.467	0.915	1.700	0.985	**0.789**
20	0.2	0.1	1.535	0.895	0.974	0.782	**0.463**
20	0.2	0.3	1.963	0.916	1.541	0.923	**0.598**
20	0.2	0.5	2.512	0.915	1.883	0.984	**0.772**
20	0.3	0.1	1.622	0.891	0.960	0.834	**0.486**
20	0.3	0.3	1.983	0.915	1.659	0.935	**0.617**
20	0.3	0.5	2.484	0.917	1.870	0.993	**0.785**
20	0.4	0.1	1.559	0.894	0.932	0.798	**0.489**
20	0.4	0.3	1.800	0.917	1.562	0.904	**0.637**
20	0.4	0.5	2.368	0.917	1.913	0.973	**0.804**

C.3 Robustness under More Realistic Shift Patterns

We further expanded our simulation experiments beyond sparse Gaussian contrasts. Specifically, we consider dense shifts, structured shifts, and correlated parameter contrasts $\boldsymbol{\delta}^{(k)}$. We consider the following three patterns for the parameter shift vector $\boldsymbol{\delta}^{(k)}$.

(i) Log-normal dense shift. For each task k, we set $\boldsymbol{\delta}_j^{(k)} = s_j \exp(Z_j)$ for all $j \in [p]$, where $s_j \sim \text{Uniform}\{-1, +1\}$ and $Z_j \sim \text{N}(\mu_\sigma, \sigma^2)$ i.i.d. The location μ_σ is calibrated so that $\mathbb{E}[\|\boldsymbol{\delta}^{(k)}\|^2] = h^2/50$. Larger σ concentrates energy into fewer coordinates despite all p being nonzero; we report effective sparsity as the fraction of coordinates accounting for 90% of total shift energy.

(ii) Hierarchical shift. For each task k, we sample a set of primary features P_k of size 10 and secondary features S_k of size 40 from the remaining coordinates. We set $\boldsymbol{\delta}_j^{(k)} \sim \text{N}(0, (3h/|P_k|)^2)$ for $j \in P_k$, $\boldsymbol{\delta}_j^{(k)} \sim \text{N}(0, (0.5h/|S_k|)^2)$ for $j \in S_k$, and $\boldsymbol{\delta}_j^{(k)} = 0$ otherwise. This induces a few large shifts and many smaller shifts.

(iii) Correlated shift. We select a common support $H \subset [p]$ with $|H| = 50$. For each $j \in H$, the vector $(\boldsymbol{\delta}_j^{(1)}, \ldots, \boldsymbol{\delta}_j^{(K)})^\top$ is sampled from a mean-zero multivariate normal distribution with covariance $(h/50)^2 \boldsymbol{\Sigma}_\delta$, where $\boldsymbol{\Sigma}_\delta$ has unit diagonal and constant off-diagonal correlation ρ_δ. This yields cross-task correlation in the non-transferable components.

As reported in Table A.4, A.5 and A.6, *TransMission* continues to outperform competing methods in most settings, particularly when the heterogeneity level (as measured by h) is high. These additional results demonstrate that the proposed method remains robust under more general and realistic forms of inter-task heterogeneity.
